# Highly Selective *p*-Xylene Separation from Mixtures of C8 Aromatics by a Nonporous Molecular Apohost

**DOI:** 10.1021/jacs.3c07198

**Published:** 2023-12-06

**Authors:** Maryam Rahmani, Catiúcia R. M. O. Matos, Shi-Qiang Wang, Andrey A. Bezrukov, Alan C. Eaby, Debobroto Sensharma, Yassin Hjiej-Andaloussi, Matthias Vandichel, Michael J. Zaworotko

**Affiliations:** † Bernal Institute, Department of Chemical Sciences, University of Limerick, Limerick V94 T9PX, Republic of Ireland; ‡ Institute of Materials Research and Engineering (IMRE), Agency for Science, Technology and Research (A*STAR), 2 Fusionopolis Way, 138634 Singapore

## Abstract

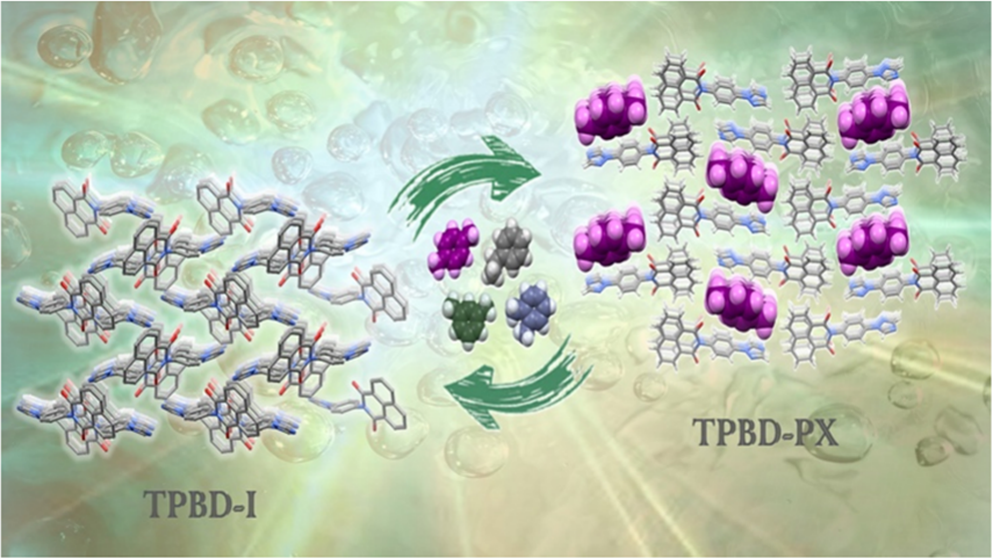

High and increasing production of separation of C8 aromatic isomers demands the development of purification methods that are efficient, scalable, and inexpensive, especially for *p*-xylene, PX, the largest volume C8 commodity. Herein, we report that 4-(1*H*-1,2,4-triazol-1-yl)-phenyl-1*H*-benzo[de]isoquinoline-1,3(2H)-dione (**TPBD**), a molecular compound that can be prepared and scaled up via solid-state synthesis, exhibits exceptional PX selectivity over each of the other C8 isomers, *o*-xylene (OX), *m*-xylene (MX), and ethylbenzene (EB). The apohost or α form of **TPBD** was found to exhibit conformational polymorphism in the solid state enabled by rotation of its triazole and benzene rings. **TPBD-αI** and **TPBD-αII** are nonporous polymorphs that transformed to the same PX inclusion compound, **TPBD-PX**, upon contact with liquid PX. **TPBD** enabled highly selective capture of PX, as established by competitive slurry experiments involving various molar ratios in binary, ternary, and quaternary mixtures of C8 aromatics. Binary selectivity values for PX as determined by ^1^H NMR spectroscopy and gas chromatography ranged from 22.4 to 108.4, setting new benchmarks for both PX/MX (70.3) and PX/EB (59.9) selectivity as well as close to benchmark selectivity for PX/OX (108.4). To our knowledge, **TPBD** is the first material of any class to exhibit such high across-the-board PX selectivity from quaternary mixtures of C8 aromatics under ambient conditions. Crystallographic and computational studies provide structural insight into the PX binding site in **TPBD-PX**, whereas thermal stability and capture kinetics were determined by variable-temperature powder X-ray diffraction and slurry tests, respectively. That **TPBD** offers benchmark PX selectivity and facile recyclability makes it a prototypal molecular compound for PX purification or capture under ambient conditions.

## Introduction

Purification of the C8 aromatic isomers *o*-xylene (OX), *m*-xylene (MX), *p*-xylene (PX), and ethylbenzene (EB) occurs at an industrial scale as each is a high-volume product of the chemical industry.^1−3^ Among these isomers, PX is of note as it is a precursor for terephthalic acid, which in turn is used to produce polyester and polyethylene terephthalate. Demand for PX is increasing at 6–8% per year and accounts for >86% of the global mixed xylenes market in 2021.^4^ The similarity of the C8 aromatic isomers with respect to their physicochemical properties (Table S1)^5^ complicates their separation from each other, and conventional purification methods such as distillation or fractional crystallization require controlled environments with either high or low temperatures, respectively. Therefore, there is interest in the development of novel sorbents or membrane-based technologies that can provide more energy-efficient purification of PX.^6,7^ Whereas selective PX adsorption using FAU-type zeolites has been validated, relatively high temperatures and pressures on simulated moving beds (SMBs) are required and PX selectivity is usually <10.^5^

Physisorbents such as metal–organic materials (MOMs),^8−14^ especially flexible MOMs (FMOMs),^15−19^ have exhibited promising characteristics for C8 separation.^18,20−27^ Notably, two-dimensional (2D) layered coordination networks,^28,29^ one-dimensional (1D) chain coordination polymers,^30,31^ and zero-dimensional (0D) coordination complexes^32−35^ can offer selective binding of C8 guests through C8-induced structural phase transformations from guest-free apohosts (α-phases) to inclusion compounds (β-phases).^36^ Organic molecular solids can also undergo phase transformations induced by guest molecules^37−41^ and offer selective binding of C8 isomers.^32,33,42−46^ In terms of selectivity, a cage host with intrinsic porosity was found to offer across-the-board PX selectivity ranging between 6.60 and 12.10.^36^ In addition, studies suggest that flexibility in molecular compounds can enable selective clathration and accelerated diffusion of PX through porous structures.^43,47,48^ Stimuli-induced phase transformations of 0D apohosts conducted by the Atwood and Barbour groups provided insight into how external stimuli can trigger structural changes and their potential applications.^33,44,49,50^ We have recently coined the term switching adsorbent molecular materials (SAMMs)^32^ for those molecular compounds that switch between nonporous and porous phases when exposed to gas, vapor, or liquid adsorbates.

Organic inclusion compounds, exemplified by urea, thiourea, quinol, phenol, and Dianin’s compound, have been extensively studied with respect to their host–guest chemistry^51−55^ and selective enclathration of guest molecules. However, despite combining low cost and comparative simplicity, this class of materials remains understudied in the context of C8 separations. It has been reported that nonporous molecular solids, e.g., bishydrazones,^56^ can distinguish one isomer from a mixture of C8 aromatics, highlighting the significance of phase transformations and conformational flexibility in adapting host cavities to fit guest molecules.^57^ We herein report the C8 aromatic inclusion properties of a hitherto unstudied molecular compound, 4-(1*H*-1,2,4-triazol-1-yl)-phenyl-1*H*-benzo[de]isoquinoline-1,3(2*H*)-dione, **TPBD**, which was prepared by cocrystal-controlled solid-state synthesis^58−60^ (solvent-drop grinding, SDG,^61−63^ followed by heating). TPBD’s selection is rooted in its inherent structural features and the aforementioned studies on molecular compounds such as Werner complexes.^32−34^ The studies of Werner clathrates highlight that molecular compounds comprising aromatic rings and conformational diversity can form host–guest complexes with C8 isomers. TPBD is also attractive because it can be prepared in high yield using mechanochemistry.^64^ Single-crystal X-ray diffraction (SCXRD), powder X-ray diffraction (PXRD), and solubility studies provide insight into phase switching of **TPBD** upon exposure to liquid C8 aromatic mixtures at room temperature.

## Results and Discussion

### Crystal Structure of TPBD and its Polymorphism

**Figure 1:**
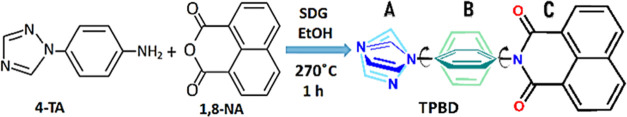
Synthesis of TPBD-αI via solid-state synthesis.

**Figure 2:**
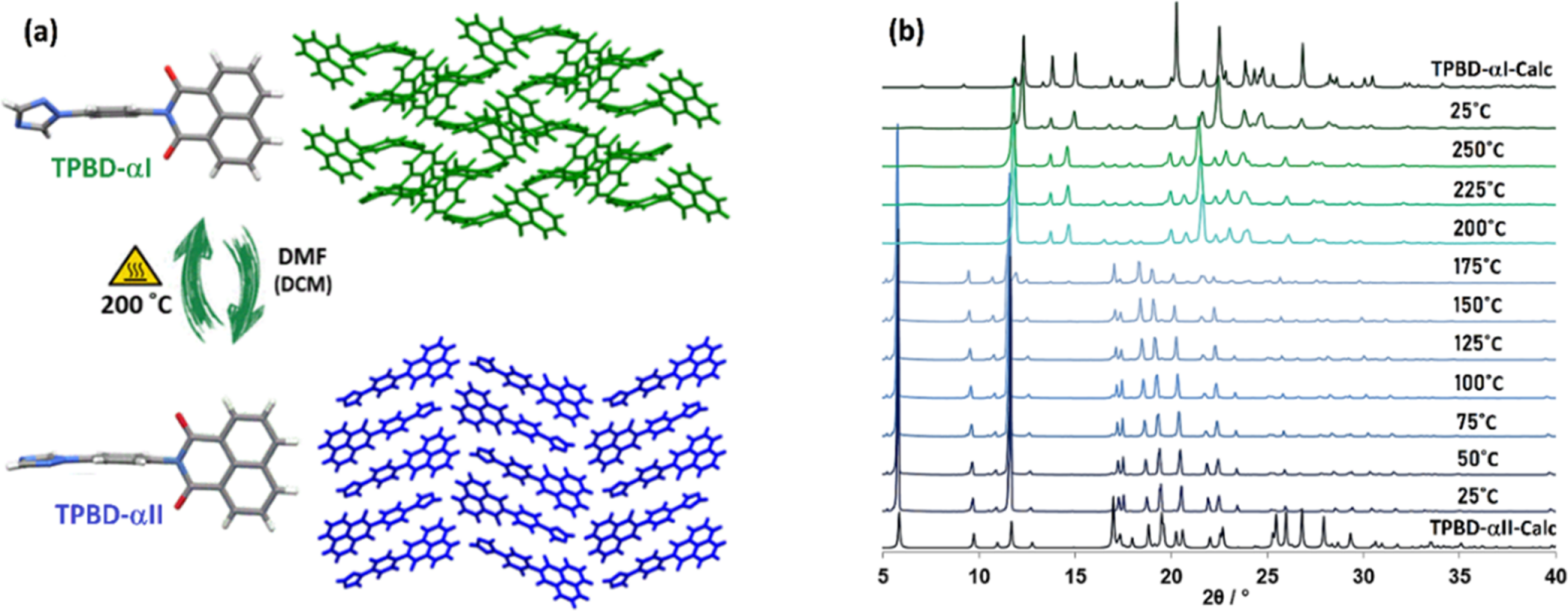
(a) Solvent and temperature-mediated conformational polymorphism of **TPBD**. (a) Illustration of the formation of **TPBD-αII** from the recrystallization of **TPBD-αI** in DMF or DCM and subsequent recovery of **TPBD-αI** by heating. (b) VT-PXRD indicates that conversion of **TPBD-αII** to **TPBD-αI** occurs after heating to temperatures ≥200 °C and that this structure is maintained when cooled to 25 °C.

A 1:1 mixture of 1,8-naphthalic anhydride (1,8-NA) and 4-(1,2,4-triazol-1-yl) aniline (4-TA) was wet ground with EtOH before heating at 270 °C for 1 h to form **TPBD** (Figures 1 and S1a). SCXRD data revealed that the colorless blocks, grown by crystallizing **TPBD** from EtOH, belong to the monoclinic space group *P*2_1_/*c* with one **TPBD** in the asymmetric unit (Tables S2 and S3). This crystal form, **TPBD-αI**, was also crystallized from water, methanol, ethanol, acetone, acetonitrile, *n*-hexane, isopropanol, and n-propanol (Figure S2). Recrystallization of **TPBD-αI** from dimethylformamide and dichloromethane afforded colorless needle-like crystals of a second polymorph, **TPBD-αII**, which adopted orthorhombic space group *P*2_1_2_1_2 (Figures 2a, S1b and Table S3). The main difference between **TPBD-αI** and **TPBD-αII** is the orientation of the triazole and benzene moieties, resulting in different unit cell parameters and a slightly reduced unit cell volume (Table S3). During a study of reaction conditions to optimize the synthesis of **TPBD** (Figures S3a,b), two additional polymorphs were found. **TPBD-αIII** and **TPBD-αIV** were isolated as minor crystalline impurities from the bulk reaction product but could not be reproduced with bulk phase purity using solvent-mediated methods, which consistently afforded **TPBD-αI** or **TPBD-αII**. **TPBD-αIII** crystallized in the orthorhombic space group *P*2_1_2_1_2, whereas **TPBD-αIV** crystallized in the triclinic space group *P*1̅ (Table S3). The relative stability of these polymorphs is suggested by their respective densities^65,66^ of 1.495, 1.533, 1.423, and 1.506 g/cm^3^.

To simplify the analysis of **TPBD**’s conformational features, we label the 1,2,4-triazole ring A, the benzene ring B, and the rest of the structure C (Figure 1). **TPBD**’s structural flexibility is evident from rotation of the triazole and benzene rings as depicted in Table S4 and Figure S4. The magnitude of the torsion angles formed between the planes of A and B are 33.6, 4.9, 14.6, and 32.4° in **TPBD-αI-αIV**, respectively. Figure S4 shows that **TPBD-αI** and **TPBD-αII** have opposite orientations for rings A and B compared to **TPBD-αIII** and **TPBD-αIV**. **TPBD-αI** and **TPBD-αII** have similar orientations, but the triazole ring in **TPBD-αI** is twisted more significantly, while **TPBD-αIII** and **TPBD-αIV** have similar conformations. The conformational variability of the A/B rings and B/C moieties (Figure S4) can explain the conformational polymorphism seen herein. CH···π and π···π stacking interactions play a key role in the crystal packing in **TPBD-αI-αIV** (Figure S5).

The four TPBD polymorphs were optimized with density functional theory (DFT) to determine their relative stability in kilojoules per mol_TPBD_ at 0 K. The cell parameters for structures before and after cell optimization are displayed in Tables S5 and S6. In decreasing order of stability, we found **TPBD-αII** (0.0) > **TPBD-αI** (1.3) > **TPBD-αIII** (3.4) > **TPBD-αIV** (4.4). The relative energy differences between these four cell-optimized **TPBD** polymorphs are thus small, and with the cell-optimized structures, qualitative stability trends at different temperatures can be predicted (Table S7). Furthermore, other synthesis parameters, such as temperature, pressure, and solvation, will also contribute to the relative stability, as observed experimentally.

Following unsuccessful attempts to reproduce **TPBD-αIII** and **TPBD-αIV**, our focus shifted to preparing bulk samples of **TPBD-αI** and **TPBD-αII** and an analysis of their properties. Thermogravimetric analysis (TGA) revealed that **TPBD-αI** and **TPBD-αII** did not exhibit weight loss below 300 °C (Figure S6a). Differential scanning calorimetry (DSC) (closed pan) revealed one endothermic event in the DSC curve of **TPBD-αI** (Figure S6b), which we attribute to melting at 328 °C. The DSC curve of **TPBD-αII** showed two endotherms, the first consistent with a phase transition at 185 °C, followed by a sharp endothermic peak at 334 °C consistent with melting. Interestingly, the free energies of formation for both **TPBD-αI** and **TPBD-αII** polymorphs become endergonic at approximately 330 °C, which agrees with the experimentally observed melting temperatures (Figure S7). Variable-temperature powder X-ray diffraction (VT-PXRD) revealed that **TPBD-αII** converted to **TPBD-αI** at 175–200 °C (Figure 2b). This experimentally observed stability reversal is also observed computationally: the Helmholtz free energy differences between **TPBD-αI** and **TPBD-αII** are negligible at 50 °C; however, at higher temperatures, **TPBD-αI** becomes the thermodynamically stable polymorph (Table S7). Furthermore, accelerated stability testing^67,68^ in a humidity chamber (75% RH, 40 °C) revealed that **TPBD-αI** and **TPBD-αII** are hydrolytically stable, as confirmed by PXRD patterns remaining unchanged (Figure S8).

### C8 Aromatic Inclusion and Separation Studies

#### Single-Component Enclathration of C8 Aromatic Isomers

**Figure 3:**
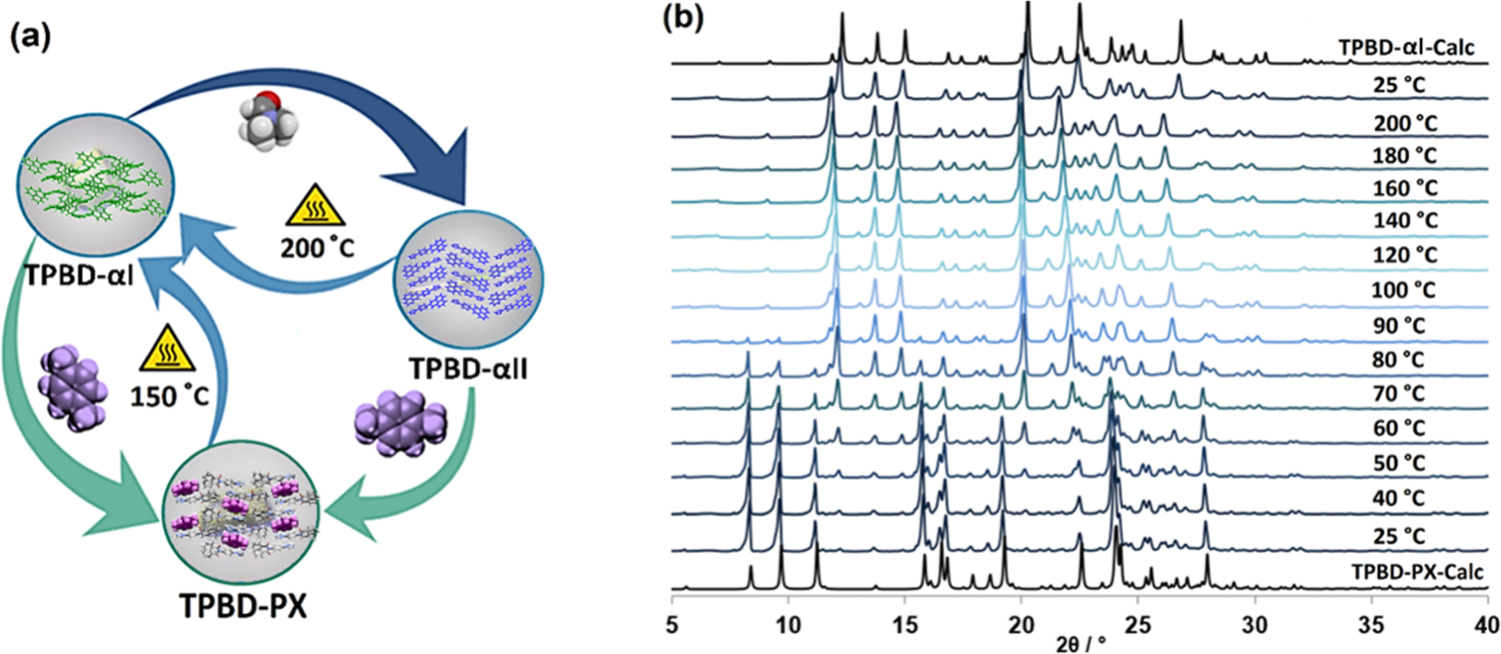
(a) Interconversion of TPBD-αI, TPBD-αII, and TPBD-PX; (b) VT-PXRD study of TPBD-PX reveals transformation to TPBD-αI upon heating.

The flexibility of **TPBD** prompted us to study its potential to serve as a host for C8 aromatic isomers. We first studied the effect of subjecting **TPBD-αI** and **TPBD-αII** to pure C8 isomers at room temperature (25 ± 3 °C) by slurry or liquid immersion in sealed vials. We observed through PXRD, ^1^H NMR, and SCXRD analyses that only PX induced transformation to an inclusion compound, **TPBD-PX**, despite prolonged exposure to the C8 isomers (ambient conditions for 4 days, Figures 3a and S9–S15): **TPBD-PX** crystallized in the space group *P*2_1_/*n* with two **TPBD** molecules and one PX in the asymmetric unit (Table S3). Saturation was attained in 20 min for **TPBD-αI** and within 25 min for **TPBD-αII** (Figure S16). Slurry experiments revealed that whereas both polymorphs are practically insoluble after several hours, 3.7 and 8.6 ng/mL for **TPBD-αI** and **TPBD-αII**, respectively, the dissolution profiles revealed a rapid initial increase in solubility (*C*_max_ = 66.8 ± 28.1 ng/mL for **TPBD-αI**; *C*_max_ = 82.1 ± 17.5 ng/mL for **TPBD-αII**). This “spring-and-parachute” effect (Figure S24 and Table S8) is well-known in pharmaceutical science and is consistent with dissolution followed by recrystallization of a less soluble phase, e.g., a hydrate, solvate, or more stable polymorph.^69^ In this case, it indicates dissolution of **TPBD** followed by the crystallization of **TPBD-PX**. PXRD analysis (Figure S16) of aliquots removed from **TPBD** slurries indicates that conversion from **TPBD-αI** or **TPBD-αII** to **TPBD-PX** had occurred. To investigate the mechanism of phase change, time-lapse photomicroscopy experiments were conducted (Movies S1, S2, S3, S4 and Figures S17–S22). These PX immersion experiments revealed that smaller particles (<100 μm) of **TPBD-αI** and all particles of **TPBD-αII** dissolved during PX exposure (1300 min) before recrystallizing as **TPBD-PX**. Larger particles of **TPBD-αI** did not dissolve, and SCXRD analysis (Figure S23) of a single crystal after PX exposure (15 h) revealed that transformation to **TPBD-PX** had occurred in the solid state (see SI for details). These data are consistent with concomitant recrystallization and adsorption. PX desorption from **TPBD-PX** was studied by using VT-PXRD under N_2_ flow. Peaks corresponding to **TPBD-αI** appeared after heating to ca. 60–90 °C, and phase transformation was complete by *ca*. 100 °C (Figure 3b). Upon cooling the sample to 25 °C, **TPBD-αI** was maintained (Figure 3b). TGA analysis revealed that loss of PX occurred at 120 °C with a weight loss of 13.5% and that the apohost remained stable up to 297 °C (Figure S25a). The DSC curve of **TPBD-PX** (closed pan) revealed a first endothermic event at approximately 185 °C, corresponding to loss of PX, and a second endotherm at ca. 328 °C that we attribute to melting (Figure S25b). Conducting PX dynamic vapor sorption tests on **TPBD-αI** did not result in a phase transformation (Figure S26). However, exposing **TPBD-αI** to pure PX vapor or the vapor formed from an equimolar mixture of C8 isomers at 25 °C (30 days) and 50 °C (14 days) resulted in phase transformation to **TPBD-PX** (Figure S27).

### Multicomponent Enclathration of C8 Aromatic Isomers

Since C8 aromatics are usually separated in the liquid phase at an industrial scale, we evaluated the specificity of the apohost by immersing **TPBD-αI** in binary liquid mixtures of different ratios (1:1, 1:4, 1:9, 1:19, 1:49, and 1:99 mol/mol, Figures S28–S30). ^1^H NMR was employed to determine selectivity after slurry-assisted immersion of *ca*. 34 mg of **TPBD-αI** in *ca*. 1 mL of liquid mixture for 4 days at ambient temperature. For PX-containing mixtures, the apohost displayed strong preference toward PX. Equimolar binary separations revealed selectivity of 76.1, 22.1, and 49.2 for PX/OX, PX/MX, and PX/EB, respectively (Table 1 and Figures S31–S33). High selectivity of **TPBD-αI** toward PX was corroborated by exposing it to binary mixtures with lower PX concentration. It was determined that **TPBD-αI** exhibited PX/OX selectivity of 108.4, 105.6, and 97.1 for PX/OX mixtures with ratios of 1:4, 1:9, and 1:19, respectively (Table 1 and Figures S34–S38). Additionally, PX/MX selectivity values of 45.9, 65.8, and 70.3 for mixtures with ratios of 1:4, 1:9, and 1:19 were observed (Table 1 and Figures S39–S43).

**Table 1:** Selectivity Coefficients for Liquid Mixtures of C8 Isomers at 25 °C

composition	ratio in liquid	ratio absorbed	selectivity coefficient
TPBD-αI			
PX/MX	1:1	59.7:1.2	50.6^G^
	1:1	22.1	22.1^N^
	1:4	11.6	45.9^N^
	1:9	7.3	65.8^N^
	1:19	3.7	70.3^N^
	1:49	No C8 uptake	No C8 uptake
	1:99	No C8 uptake	No C8 uptake
PX/OX	1:1	49.6:0.9	54.1^G^
	1:1	76.1	76.1^N^
	1:4	27.5	108.4^N^
	1:9	10.8	105.6^N^
	1:19	5.0	97.1^N^
	1:49	No C8 uptake	No C8 uptake
	1:99	No C8 uptake	No C8 uptake
PX/EB	1:1	52.0:1.1	47.3^G^
	1:1	49.2	49.2^N^
	1:4	14.6	59.9^N^
	1:9	6.5	58.4^N^
	1:19	3.0	57.1^N^
	1:49	0.9	46.0^N^
	1:99	No C8 uptake	No C8 uptake
PX/OX/MX	1:1:1	49.4:0.9:0.8^4d^	60.4^G^
		66.3:1.0:2.4^lh^	39.4^N^
		104.2:1.0:2.7^4d^	56.3^N^
PX/OX/MX/EB	1:1:1:1	87.1:1.0:1.1:0.9^4d^	85.7^G^
		89.6:1.0:3.1:1.5^4d^	48.5^N^
		50.7:1.2:1.9:1.0^a^	37.5^N^
TPBD-αII			
PX/OX/MX	1:1:1	78.7:1.0:2.6^1h^	43.7^N^
		82.4:1.0:2.8^4d^	43.6^N^
PX/OX/MX/EB	1:1:1:1	84.6:1.0:2.5:1.8^4d^	47.9^N^

a Selectivity after 10 cycles in 2 h′ immersion. *N* = ^1^H NMR G = GC.

Overlapping of PX and MX ^1^H NMR peaks impact the accuracy of values at higher amounts of PX. For PX/EB; selectivities were determined to be 59.9, 58.4, 57.1, and 46.0 for 1:4, 1:9, 1:19, and 1:49 mixtures, respectively (Table 1 and Figures S44–S49).

No PX uptake was observed for PX/MX or PX/OX in 1:49 and 1:99 binary mixtures or with PX/EB in 1:99 binary mixtures. In the absence of PX, **TPBD** does not form inclusion compounds with the other C8 isomers, further emphasizing the high selectivity of **TPBD** for PX. For binary C8 mixtures, PX selectivity increased for PX/MX mixtures as a function of MX concentration (Figure S50a), while for other mixture compositions (PX/OX, PX/EB), no clear trend was observed. TGA showed PX uptake from the binary mixtures consistent with that of single-component PX (14.8 wt % loss), indicating preferential PX enclathration from binary mixtures (Table 1 and Figure S50a). **TPBD-αI** displayed selectivities, ranging from 76 to 108, that exceed the current benchmarks for PX/OX binary mixtures: Previously, the highest selectivities were reported for a 0D **Cu-metallocycle**,^44^ a 1D coordination polymer, **Mn-dhbq**,^31^ and three-dimensional (3D) MOFs **ZIF-67** and **ZIF-8**,^22^ with PX/OX selectivities of 51.6, 66.8, 98.9, and 81.2, respectively. **MAF-89**, comprised of 3D-connected quasi-discrete pores, shows PX/OX selectivity of 221 that surpasses **TPBD-αI** (Figure S50b and Table S9).

**Figure 4:**
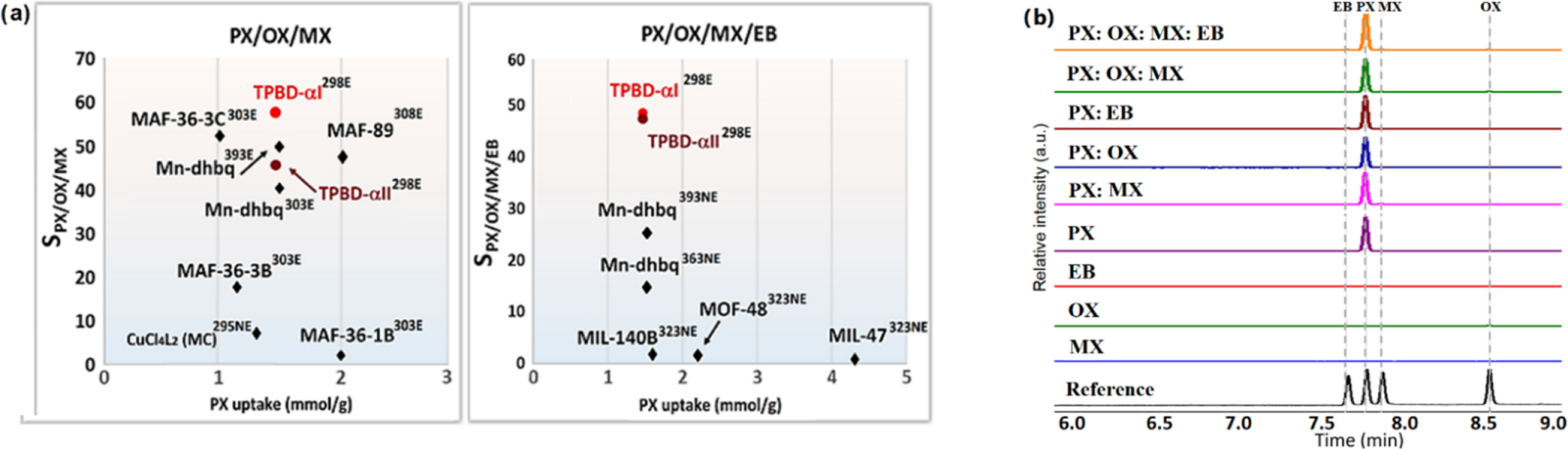
(a) Comparison of nonporous (●) and porous (⧫) absorbents for PX separation from ternary and quaternary mixtures. The experimental conditions may vary, like temperature and composition (E: equimolar, NE: nonequimolar mixture). (b) Gas chromatograms used to quantify the composition of TPBD crystals exposed to pure C8 isomers or approximately equimolar mixtures of C8 isomers at RT.

The separation performances of **TPBD-αI** and **TPBD-αII** for ternary and quaternary mixtures (Figure S51) were also investigated by using slurries at 25 °C (Figure S52). **TPBD-αI** and **TPBD-αII** were immersed in equimolar PX/MX/OX and PX/MX/OX/EB mixtures, filtered, and air-dried under ambient conditions. TGA curves of **TPBD-αI** after exposure to ternary and quaternary mixtures resulted in weight losses consistent with the PX loading obtained from binary mixtures and pure PX (14.9 wt %, Figure S53). **TPBD-αI** exhibited a selectivity of 56.3 (104.2/2.7/1.0) for equimolar ternary PX/MX/OX mixtures (Table 1 and Figure S54). These values surpass the selectivity of current benchmark sorbents **MAF-36–3C** (51.3 at 25 °C),^19^**Mn-dhbq** (48.3 at 120 °C),^31^ and **MAF-89** (46.4 at 35 °C).^20^ Furthermore, while **TPBD-αII** (*S*_PX/OX/MX_ = 43.6) displayed a slightly lower selectivity than previous benchmarks, its performance remains high (Figures 4a and S55).

**Figure 5:**
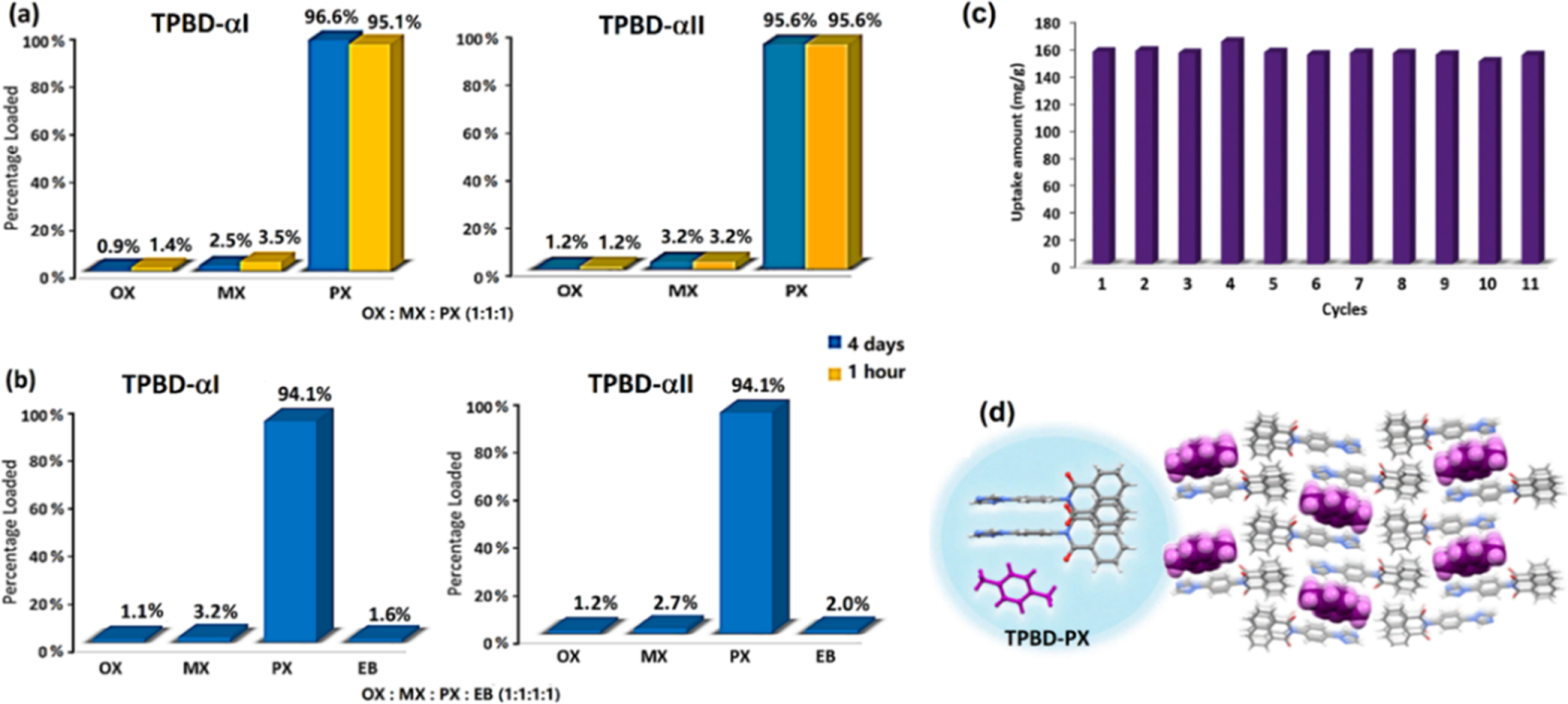
(a, b) Separation performance of TPBD-αI and TPBD-αII upon exposure to equimolar ternary and quaternary mixture of C8 isomers at 293 K. (c) TPBD-αI recyclability after 11 consecutive cycles of PX enclathration/release. (d) Asymmetric unit and crystal packing of TPBD-PX.

Concerning purification of PX from EB, literature reports are limited, likely reflecting that sorbents tend to exhibit relatively low PX/EB selectivity, making them unsuitable for purifying PX when EB is present.^70^**TPBD** retained its high PX selectivity in the presence of EB in binary, ternary, and even quaternary mixtures (Table 1 and S9). According to our findings, **TPBD** sets a new benchmark for the extraction of PX from EB despite their similar kinetic diameters, not only in equimolar binary mixtures but also at low PX concentrations (2%). At ambient conditions, **TPBD-αI** and **TPBD-αII** yielded PX/OX/MX/EB selectivities of 48.5 and 47.9 (Figures S56 and S57), respectively, higher than the value of 25.1 for **Mn-dhbq** (Figure 4a, Tables 1 and S9). Owing to the consistent presence of EB in industrial feed mixtures, the affinity of **TPBD** toward PX compared to EB enables both forms of **TPBD** to outperform existing sorbents. Our data reveal that one cycle of uptake/release involving **TPBD-αI** afforded PX with purity levels of 96.6 and 94.1% from equimolar mixtures of PX/OX/MX and PX/OX/MX/EB, respectively. Values of 95.6 and 94.1% were obtained for **TPBD-αII** (Figure 5a). Notably, molecular solids, as exemplified by **EtP6β**, can exhibit higher PX purity in equimolar ternary mixtures, but this purity decreases in the presence of EB in quaternary mixtures.^41,43,45,71^ However, due to a lack of data on selectivities of current benchmark sorbents under ambient conditions, it is challenging to compare their overall performance with **TPBD**.

Enclathration of PX from equimolar ternary mixtures after 1 or 4 days of slurrying revealed almost identical outcomes (Figures 5a, S54, and S55). This indicates that the PX inclusion compound is both the kinetic and thermodynamic product. Table 1 reveals that PX selectivity improved only slightly with time, corresponding to PXRD (Figure S16) and solubility test (Figure S24 and Table S8) analyses of slurry-assisted immersions, indicating that PX exposure for 1 h is sufficient for **TPBD-αI** to reach its capacity. PXRD data revealed that **TPBD-αI** had converted to **TPBD-PX** upon exposure to an equimolar quaternary mixture of C8 aromatic isomers within 35 min (Figure S58), indicating relatively fast kinetics under slurry-assisted immersion at room temperature. This sorption time is comparable to MOFs, PCPs, and PMCs with high selectivities.^20,31,44,72^ In the liquid phase, **MAF-36**-**3C** (*S*_PX/OX/MX_ = 51.3) and **3B** (*S*_PX/OX/MX_ = 16) exhibited varying adsorption rates, taking 24 and 3 h, respectively, whereas form **1B** (*S*_PX/OX/MX_ = 15) exhibited fast uptake in 2 min attributed to smaller crystal size.^19^**MAF-89** achieved over 90% capacity of its equilibrium amount within 15 min upon exposure to an equimolar ternary xylene mixture.^20^ We note that comparison to kinetics reported in the literature is only qualitative/indicative, as uptake kinetics depend on multiple factors such as sample mass, particle shape/size, and grain size. Carefully controlled experimental conditions are needed for quantitative comparison of sorption kinetics.^73,74^

For practical applications, stability over multiple sorption cycles is required, so recyclability of **TPBD-αI** was studied. **TPBD-αI** retained its working capacity (approximately 13.5 wt % or 156.5 mg/g uptake) after 11 consecutive cycles of static immersion in pure PX and enclathration, followed by PX removal by heating to 150 °C (Figure 5c and S59). **TPBD-αI** could be regenerated after 11 cycles as indicated by its unchanged PXRD pattern (Figure S60). **TPBD-αI** also maintained selectivity of *S*_PX/OX/MX/EB_ = 37.5 from an equimolar mixture of PX/OX/MX/EB, confirming sustained enclathration performance after 11 cycles (Figure S61). The dynamic column separation performance of **TPBD-αI** toward liquid equimolar quaternary C8 mixtures at ambient conditions (25 ± 3 °C) was investigated and revealed selectivity of 27.39 (Figure S62). Despite the energy-efficient advantage of separation by enclathration over distillation, subjecting **TPBD-αI** to higher temperatures in contact with pure or mixed C8 isomers offers insights into its selectivity behavior under various conditions. Gas chromatography was also used to analyze the PX selectivity of **TPBD-αI** exposed to C8 mixtures at 25 and 80 °C (Table S10, Figure 4b and S63). Selectivity coefficients at 25 °C for binary (PX/MX, PX/OX, PX/EB), ternary (PX/OX/MX), and quaternary mixtures were determined to be 50.6, 54.1, 47.3, 60.4, and 85.7, respectively. At 80 °C, selectivity coefficients decreased compared to room temperature, with corresponding values of 32.6, 36.0, 25.4, 31.9, and 36.7, respectively. As revealed in Table 1, the values obtained by GC are consistent with those obtained by ^1^H NMR.

**Figure 6:**
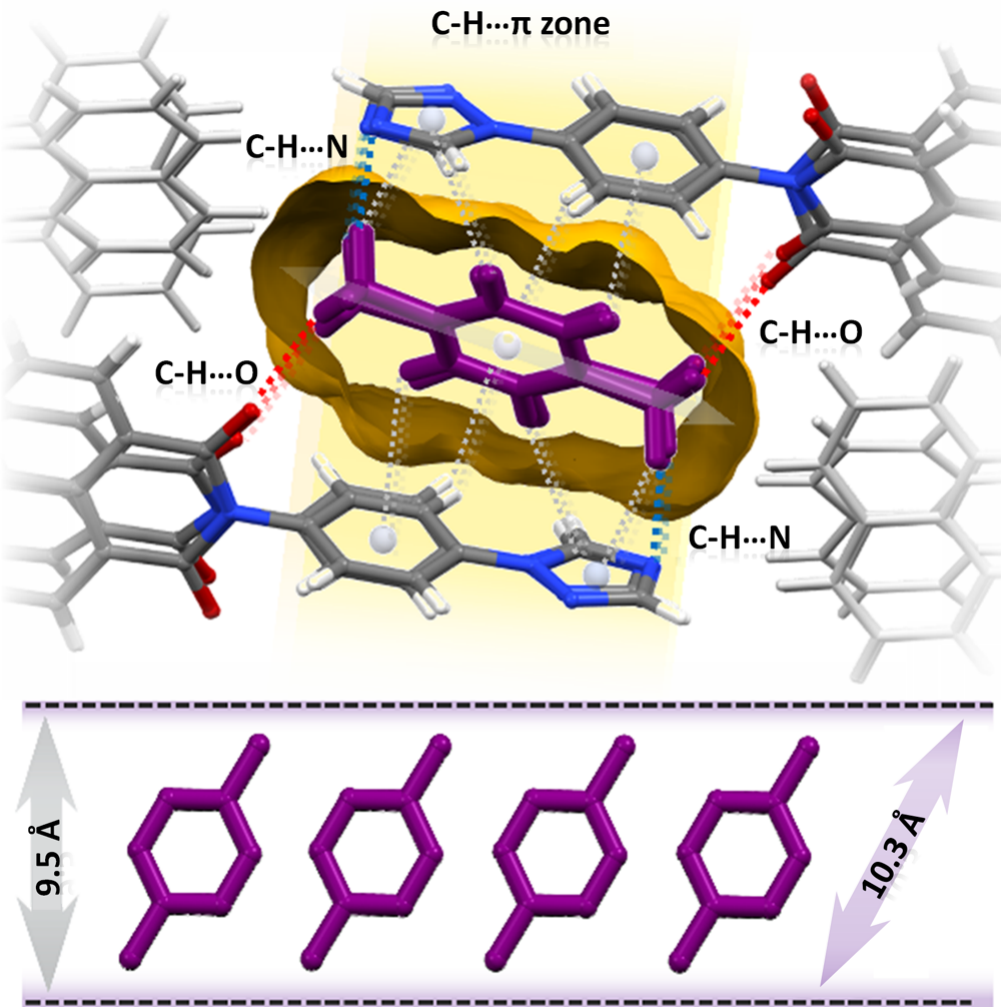
Illustration depicting the arrangement of PX molecules in TPBD-PX, highlighting that multiple C–H···π interactions occur in the yellow area, along with CH···O interactions (PX guest molecules are purple).

To gain insight into the PX selectivity of **TPBD**, we recrystallized **TPBD** from PX and determined the single-crystal structure of **TPBD-PX** (Figure 5d). The conformation of the two **TPBD** molecules differed due to slight rotation around the bond between the A and B rings (Table S4). **TPBD** dimers formed through CH···N interactions (C···N distances of 3.503 Å) between adjacent triazole rings (Figure S64a). **TPBD** molecules arrange to form 1D rectangular channels (5.44 Å × 9.07 Å) during PX uptake that propagate along [100], with void spaces of 20.1% that were sufficiently large to accommodate PX molecules (Figure S65). An analysis of the interactions in **TPBD-PX** revealed no significant π···π interactions in terms of guest–guest or host–guest binding. As shown in Figure S61b, the PX molecules do not interact with one another. Instead, PX molecules form several C–H···π interactions (Figure S64b) and CH···O hydrogen bonds (C···O distances of 3.503(3) and 3.529(3) Å with associated angles of 163.0(8) and 153.0(7)°), and CH···N (C···N distances of 3.785(3) and 3.859(3) Å with associated angles of 135.9(6) and 140.3(6)°) with **TPBD** molecules (Figures 6 and S64a).

Usually, enhanced binding of guest molecules occurs when pores exhibit a size and shape match for a specific molecule, allowing for improved selectivity over other guest species. Indeed, the 1D pores in **TPBD-PX** have dimensions of 10.30–9.07 Å at the solvent-accessible surface, comparable to the dimensions of PX (Table S1), making it a suitable match for PX inclusion but not for the other isomers. While simulation studies have previously reported the ideal dimensions of rectangular 1D channels for separating PX from other C8 isomers,^75,76^ it is important to note that the ideal size also depends on supramolecular interactions within the host–guest system and the arrangement of PX molecules.

To corroborate the experimental results showing the preference for capture of PX over other C8 isomers, the relative stability of **TPBD** inclusion compounds was determined via DFT calculations. Since PX-loaded **TPBD** could be refined by XRD, i.e., (TPBD)_8_(PX)_4_, the other guest-loaded structures were built from **TPBD-PX**. These hypothetical C8-loaded structures and **TPBD-PX** were then optimized and subsequently cell-optimized to determine the crystallization energies and Gibbs free energies starting from **TPBD-αI** and the gas phase of C8 isomers (p_C8_ = 1 bar) at 25, 50, 100, and 150 °C (in kJ/mol_C8_). The crystallization energies, i.e., −78.8 (PX), −67.8 (MX), −61.9 (OX), and −65.6 (EB) kJ/mol, are indicative of strongly exothermic crystallization processes and show a strong preference to enclathrate PX over the other C8 aromatic isomers (see Table S11). Analyses of various modeled **TPBD-C8** aromatic structures revealed predominant H···O, H···N, and C···H interactions, which control crystal packing (Figure S66). Notably, in **TPBD-PX**, stronger CH···O hydrogen bonds compared with other models influence crystal packing and selectivity for PX over the other C8 aromatic isomers.

In previous work, sorbents that exhibit discrimination for PX, typically adapt to the size and shape of PX molecules, controlling their pore structure via a gating mechanism, leading to a guest-loaded complex with the lowest binding energy and preference for PX over other C8 aromatic isomers.^19,20,77^ PX clathrate **TPBD-PX** offers precise size/shape matching to accommodate PX molecules, establishing favorable positions for C–H···π interactions along the *a*-axis. CH···O and CH···N interactions formed between the host and guest serve to anchor the PX molecules. The cavity in **TPBD-PX** formed by the triazole and benzene rings of the host is such that the second methyl group in MX and OX and the ethyl moiety of EB are hindered from the formation of favorable C–H···π, C–H···O, and CH···N interactions. We therefore attribute the separation performance of **TPBD** to its ability to adapt to PX molecules through a channel that is an excellent shape and size fit for PX vs the other C8 aromatic isomers. This feature enables **TPBD** to more selectively separate PX from the other C8 aromatic isomers in comparison to existing PX selective sorbents.

## Conclusions

In summary, our results reveal that a nonporous molecular apohost (**TPBD**) enables the highly selective separation of PX from mixtures of C8 aromatics, setting new benchmark selectivity values. PX can thereby be separated by **TPBD** as a sole substrate, even in the presence of low concentrations (5%) of PX. Whereas separation of xylene isomers has tended to focus upon porous frameworks such as zeolites and MOFs, **TPBD** further highlights^7^ that certain nonporous apohost molecules can form inclusion compounds with suitable binding sites that offer across-the-board selectivity for one of the C8 isomers. **TPBD** also offers low cost, facile synthesis, a metal-free composition, high thermal stability, high hydrolytic stability, and recyclability. Further, that the uptake kinetics in slurry are relatively rapid at room temperature means that PX can be isolated from OX, MX, and EB with high purity in only one cycle with a relatively low energy footprint. Our findings emphasize the potential utility of molecular compounds that, although nonporous as apohosts, can be highly selective through structural adaptability and induced fit, thereby enabling phase transformations to generate cavities that offer shape and size matching for a particular guest. Such sorbents are also advantageous as they are inherently facile to recycle, relying only upon weak noncovalent interactions.

## Supporting Information Available

Experimental details; materials and methods; computational studies; summary of reported adsorbents for separation of C8 aromatics; modeling section (PDF)
Speed: 210× (AVI)
Speed: 600× (AVI)
Speed: 1331× (AVI)
Speed: 2650× (AVI)

## Supplementary Material

ja3c07198_si_001.pdf

ja3c07198_si_002.avi

ja3c07198_si_003.avi

ja3c07198_si_004.avi

ja3c07198_si_005.avi
