# Supplementary material for: Highly Selective p-Xylene Separation from Mixtures of C8 Aromatics by a Nonporous Molecular Apohost
Source: J Am Chem Soc. 2023 Dec 6;145(50):27316–24. doi: 10.1021/jacs.3c07198 (PMC10739993; doi:10.1021/jacs.3c07198)
Supplement: Supplementary file 1 — ja3c07198_si_001.pdf [file ja3c07198_si_001.pdf]

Highly Selective p-Xylene Separation from Mixtures of C8 Aromatics by a Nonporous Molecular Apohost

Maryam Rahmani,[a] Catiúcia R. M. O. Matos,[a] Shi-Qiang Wang,[b] Andrey A. Bezrukov,[a] Alan C. Eaby,[a] Debobroto Sensharma,[a] Yassin H. Andaloussi,[a] Matthias Vandichel[a] and Michael J. Zaworotko*[a]

[a] *Bernal Institute, Department of Chemical Sciences, University of Limerick, Limerick V94 T9PX, Republic of Ireland.*

[b] *Institute of Materials Research and Engineering (IMRE), Agency for Science, Technology and Research (A*STAR), 2 Fusionopolis Way 138634, Singapore.*

*Email: xtal@ul.ie

Table of Contents

Experimental section 2

Materials and methods 2

Scale-up synthesis, crystallization and characterization of host-gust systems of TPBD 4

Computational studies 9

Optical microscopy 17

C8 aromatics selectivity studies of TPBD 27

Summary of reported adsorbents for separation of C8 aromatics 44

Recyclability study on TPBD 46

Dynamic separation of PX 48

Modelling section 52

References 53

(**Notice**: In this document **TPBD-I**, **TPBD-II, TPBD-III**, and **TPBD-IV** correspond to **TPBD-αI**, **TPBD-αII, TPBD-αIII**, and **TPBD-αIV**, respectively)

EXPERIMENTAL SECTION

Materials and methods

All chemicals were used as purchased from Sigma Aldrich without further purification. All C8 aromatic solvents (PX, MX, OX and EB) used during this study were of commercial purity, ≥ 99%.

Table S1. Physical Properties of C8 Aromatics

	**OX**	**MX**	**PX**	**EB**
Kinetic diameter (Å)	7.4	7.1	6.7	6.7
Boiling point (K)	417.6	412.3	411.5	409.3
Freezing point (K)	248.0	222.5	286.4	178.2
Dipole moment (D)	0.62	0.36	0	0.59
Polarizability (cm^3)	14.9	14.2	13.7	14.2
Density at 25°C (g cm^{-3})	0.876	0.861	0.858	0.867
Molecular dimensions (Å)	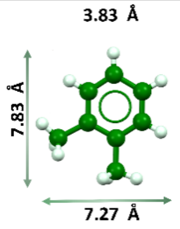	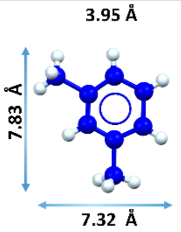	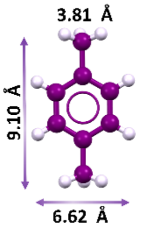	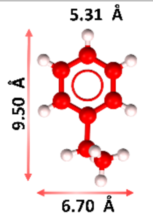

Powder X-ray Diffraction (PXRD) patterns were obtained using a PANalytical Empyrean™ diffractometer equipped with a PIXcel3D detector with the following experimental parameters: Cu K*α* radiation (λ = 1.54056 Å); 40 kV and 40 mA; scan speed 8°/min; step size 0.05°,2θ = 4–40°.

In-situ Variable Temperature Powder X-ray Diffraction (VT-PXRD) at different temperatures were recorded using a PANalytical X'Pert ProMPD diffractometer equipped with a PIXcel3D detector operating in scanning line detector mode with an active length of 4 utilizing 255 channels. Anton Paar TTK 450 stage coupled with the Anton Paar TCU 110 Temperature Control Unit was used to record the variable temperature diffractograms. The diffractometer is outfitted with and Empyrean Cu LEF (long fine-focus) HR (9430 033 7300x) tube operated at 40 kV and 40 mA; and Cu K*α* radiation (λ_α = 1.54056 Å). Continuous scanning mode with the goniometer in the theta-theta orientation was used to collect the data. In experiment, **TPBD-II** and **TPBD-PX** samples were loaded on a sample holder made for Anton Paar TTK 450 chamber. The data was collected from 4° to 40° with a step size of 0.0334225 and a scan time of 30 minutes per step. PXRD was collected under Nitrogen flow. The PXRD data of **TPBD-II** was collected at temperatures ranging 25-250°C, and then cooling to 25°C. This study for **TPBD-PX** was conducted at temperatures ranging from 25 °C to 200 °C, then cooled to 25 °C.

Single-Crystal X-ray Data (SCXRD) for reported structures determined by single crystal X-ray diffraction (SCXRD) with either Mo K*α* (λ= 0.7107 Å) radiation for **TPBD-I, TPBD-II, TPBD-III** and **TPBD-PX** or Cu K*α* (λ=1.5418 Å) for **TPBD-IV**. X-ray measurements were made using APEX 4[1] software, frames were integrated with Bruker SAINT[2] software and absorption corrections were performed using multi-scan methods. Crystal structures were solved by direct methods using OLEX2[3] and aniso-tropic displacement parameters for non-hydrogen atoms were applied. Some hydrogen atoms were placed at calculated positions and treated using a riding model whereas other H-atoms were located in the Fourier difference maps and placed geometrically. The *CrystalExplorer* 17.5[4] program package was used to calculate and analyse the Molecular Hirshfeld surfaces of each crystal structure.

Nuclear magnetic resonance (NMR) spectrum of each sample was collected in a JEOL ECX400 NMR spectrometer at 25 °C, 400 MHz. It should be noted that the NMR spectra exhibit slightly different shift (ca. 0.015 ppm). To make it easier to compare all the spectra, the relevant peaks of C8 aromatics are fixed by setting the residue peak of $CDCl_3$ with a range of 7.260-7.275 ppm.

Differential Scanning Calorimetry (DSC) was conducted using a TA Q2000 heat flux DSC. Samples were sealed in aluminium pans and a ramp rate of 10 °C/min from ambient temperature to 400 °C was applied under a N_2 atm. Measured heat flow was compared with that of empty aluminium pan.

Thermogravimetric Analysis (TGA) for all the compounds were carried out under N2 atmosphere in a TA instruments Q50 thermal analyser between room temperature and 500ºC with a ramp rate of 10 °C/min.

Elemental Analysis of carbon, hydrogen, and nitrogen (CHN) in **TPBD-I** and **TPBD-II** was performed using an Exeter Analytical CE 440 elemental analyser (University College Dublin, Microanalytical Laboratory), with resulting percentages detailed in Table S2.

Table S2. Elemental analysis results for TPBD-I and TPBD-II.

	N (%)	C (%)	H (%)
TPBD Calc.	16.46	70.58	3.55
TPBD-I Exp.	16.40	70.47	3.30
TPBD-II Exp.	16.53	70.39	3.41

High-performance liquid chromatography (HPLC) analysis was performed using a Shimadzu (LC-20A) instrument with a Gemini C18 column (250 x 4.6 x 5 µm). Detection occurred at 210 nm with an injection volume of 5 µl, flowing at 0.6 ml/min. The oven temperature was 37.5°C, and the mobile phase consisted of 100% methanol for 20 minutes. TPBD and PX eluted at 5.330 and 6.185 minutes, respectively (Figure S24a). The calibration curve was generated by plotting the ratio of analyte peak area to **TPBD-I** and **TPBD-II** peaks area against the nominal calibration standard concentrations (0.050, 0.075, 0.100, 0.125, and 0.150 mg/mL). Linear regression analysis yielded best-fit lines with correlation coefficients of 0.9718 for **TPBD-I** (y = 2E+07x) and 0.8492 for **TPBD-II** (y = 9E+06x. Afterward, solutions containing 10 mg/mL of TPBD in PX were prepared and stirred at 100 rpm at 25± 3°C. At specified intervals (0.25, 0.5, 1, 2, 5, 10, 15, 30, 60, 90, 120, and 1440 minutes), samples were withdrawn and immediately filtered through a 0.45 mm Corning syringe filter. Samples were filtered and analyzed through HPLC after dilution in methanol (Figure S24b and Table S8). **TPBD-I** and **TPBD-II** exhibited increased solubility in PX after stirring, reaching maximum solubility (C_{max}) of 66.77+28.12 and 82.10+17.46 ng/mL, respectively, within 15 seconds. Furthermore, both forms exhibited a "spring-and-parachute" effect[5] in the first 30 minutes, showing increased solubility before gradually decreasing. The average solubility of **TPBD-I** and **TPBD-II** in pure PX at room temperature was determined to be 3.74 ng/mL and 8.55 ng/mL, respectively, after 24 hours of stirring. This illustrates that **TPBD-II** is 2.4 times more soluble than **TPBD-I** (Figure S24b and Table S8).

Gas chromatography (GC) analyses were carried out on an Agilent 6890A gas chromatograph fitted with a 7683B ALS (automated liquid sampler) equipped with a flame ionization detector (FID). The column used was an Agilent DBWax column (Length: 30 m, Inner diameter: 320 µm, Film thickness: 0.25 µm). An initial temperature of 40 °C and initial hold time of two minutes were used with a ramp rate of 10 °C/min to a maximum temperature of 180 °C. The injector and detector were kept at 220 °C and nitrogen was used as carrier gas with a flow rate of 1 ml/min. 1 µL of each liquid sample was injected through the GC inlet with a split ratio of 100:1 and a split flow rate of 142.45 ml/min. Dichloromethane HPLC/GC grade 99.9% (Sigma-Aldrich) was used as eluent and solvent for the standard solutions of 500 ppm of all the C8 aromatic isomers. With the above-mentioned setup, we could obtain separate retention times for PX, MX, OX and EB. GC chromatographs for all the C8 aromatic isomers are shown in Figure 4b and S63.

Density Functional Theory calculations were performed using the projected augmented wave (PAW) formalism[6] as implemented in the Vienna Ab Initio Simulation Package (VASP 5.4.4),[7, 8] employing the BEEF-vdW exchange-correlation functional.[9] Atomic positions of 4 different TPBD polymorphs (**TPBD-I, TPBD-II, TPBD-III, TPBD-IV**) and $(TPBD)_8(pX)_4$ were optimized at their experimentally refined cell parameters (Table S5), using the conjugate gradient algorithm with force and electronic convergence criteria of 0.01 eV/Å and 10^{-6} eV, a Gaussian smearing of 0.02 eV, a plane wave energy cut-off of 550 eV. The co-crystals of TPBD and the other xylenes were constructed from $(TPBD)_8(PX)_4$, *i.e.* $(TPBD)_8(OX)_4$, $(TPBD)_8(MX)_4$, and $(TPBD)_8(EB)_4$, and were also optimized maintaining the experimental cell parameters of $(TPBD)_8(pX)_4$. The Monkhorst-Pack[10] k-point meshes were chosen to obtain a minimal k-point density of 4.1 points/$Å^{-1}$, with the k-points summarized in Table S5. The unit cell and atomic positions of all periodic structures were subsequently optimized with the above parameters and forces converged <0.005 eV/Å. Cell optimization caused a slight expansion of the unit cell parameters (compare Table S5 and Table S6). The corroboration of experimental data is most plausible with the data obtained from cell-optimized structures (Table S6), which can predict qualitative trends (see main text). A Hessian vibrational analysis was performed numerically displacing the atomic coordinates of all structures in x, y, and z-directions with ±0.01 Å to verify the structures can be classified as local minima on the potential energy surface. Very small imaginary modes were present and have been reported in Table S5 and Table S6; these modes were replaced with 50 cm^{-1} for the calculation of the crystal and inclusion formation enthalpies, zero-point corrected energies and free energies employing the post-processing toolkit TAMKIN.[11] The relative energies of the 4 different TPBD polymorphs are compared in Table S7. Remark that the enclathration energy for PX does only slightly change before and after cell optimization (Table S11), which could be expected as the interactions were already optimal for PX. For the other C8 isomers, cell optimization leads to higher crystallization energies by about 6 to 11 kJ/mol.

Enclathration kinetics studies involved testing TPBD apohost's enclathration for pure PX or a 1:1:1:1 mixture of C8 isomers at 25±3 °C. Approximately 50 mg of apohosts were immersed in 5 mL of the C8 isomer liquid mixture with stirring at 100 rpm. Samples of 100 µL were collected at 2-minute intervals up to 60 minutes. Each sample was filtered, dried for 5 hours at ambient conditions, and then analyzed for PX sorption using PXRD analysis.

Scale-up synthesis of TPBD-I:

TPBD-I was synthesised via the Cocrystal Controlled Solid-State Synthesis (C^3S^3)[12] technique by grinding a mixture of 995 mg (5mmol) of 1,8-Naphthalic anhydride (1,8-NA) and 801 mg (5mmol) of 4-(1,2,4-Triazol-1-yl) aniline (4-TA) through solvent drop grinding (SDG) in 200 µL of ethanol (recommended by solvent selection guidelines developed by industry) (Figure 1). **TPBD-I** was prepared in high yield (> 94%) by heating the intermediate for 1 hour at 270 °C. Then, the product was collected and washed with EtOH.

Single crystal preparation:

Synthesis of TPBD-I: The block colorless crytals of **TPBD-I** was prepared by mixing 1 ml of EtOH with 30 mg of **TPBD**, heating the mixture for an hour at 80 °C, and then letting it cool to room temperature over two days (Table S3).

Synthesis of TPBD-II: TPBD-II single crystals were made using the heat and cool technique, which included dissolving 100 mg (2.5 mmol) of **TPBD-I** in 5ml of DCM or DMF, heating to 80 °C to obtain a clear solution, then cooling to room temperature, and collecting the coloured needle crystals (Table S3).

Synthesis of TPBD-PX: TPBD-PX peach block crystals were made by mixing 1 ml of PX with 30 mg of **TPBD-I**, heating the mixture for an hour at 80 °C, and then letting it cool to room temperature (Table S3).

Table S3. Crystallographic data and structure refinement details of TPBD polymorphs and TPBD-PX.

	**TPBD-I**	**TPBD-II**	**TPBD-IIRT**	**TPBD-III**	**TPBD-IV**	**TPBD-PX**
Formula	$C_{20}H_{12}N_4O_2$	$C_{20}H_{12}N_4O_2$	$C_{20}H_{12}N_4O_2$	$C_{20}H_{12}N_4O_2$	$C_{20}H_{12}N_4O_2$	$C_{48}H_{34}N_8O_4$
Formula weight	340.34	340.34	340.34	340.34	340.34	786.86
Crystal system	monoclinic	orthorhombic	orthorhombic	orthorhombic	triclinic	monoclinic
Space group	$P\ 2_1/c$	$P2_12_12$	$P2_12_12$	$P2_12_12_1$	$P\bar{1}$	$P2_1/n$
a(Å)	12.7589(4)	9.3900(3)	9.5376(4)	5.4652(2)	7.7712(2)	10.7208(4)
b(Å)	14.8509(4)	30.0460(10)	30.2809(10)	15.9871(8)	9.4957(2)	31.4461(12)
c (Å)	8.0666(2)	5.2260(2)	5.2172(2)	18.1854(9)	11.3201(3)	11.0004(5)
α(˚)	90	90	90	90	100.3790(10)	90
β(˚)	98.498(1)	90	90	90	103.7700(10)	95.5620(10)
γ(˚)	90	90	90	90	106.3830(10)	90
Volume/$Å^3$	1511.69(7)	1474.42(9)	1506.76(10)	1588.91(13)	750.48(3)	3691.1(3)
ρ_{calc} (g cm^{-3})	1.4953	1.5331	1.5002	1.4226	1.5060	1.4159
Z	4	4	4	4	2	4
θ range	2.90-27.33	2.97-26.366	2.94-27.500	2.78-27.56	4.17-72.36	2.72-27.55
N_{ref}/N_{para}	3414-235	3028-236	3462-235	3659-235	2912-236	8468-543
Temperature/K	150.0	150.0	301.0	150.0	100.0	150.0
F(000)	704.3	704.0	704.3	704.3	352.0	1640.7
Radiation	Mo *K*\a	Mo *K*\a	Mo *K*\a	Mo *K*\a	Cu *K*\a	Mo *K*\a
Goodness-of-fit on F^2	1.079	1.061	1.068	1.062	1.094	1.035
Final R indexes[I>=2σ (I)]	$R1$= 0.0362,	$R1$= 0.0349,	$R1$= 0.0397,	$R1$ = 0.0421,	$R1$= 0.0763,	$R1$= 0.0552,
	wR_2 = 0.0936	wR_2 = 0.0728	wR_2 = 0.0851	wR_2 = 0.0801	wR_2 = 0.2119	wR_2 = 0.1081
Final R indexes [all data]	$R1$ = 0.0459,	$R1$ = 0.0476,	$R1$ = 0.0624,	$R1$ = 0.0678,	$R1$ = 0.0860,	$R1$ = 0.1052,
	wR_2 = 0.1026	wR_2 = 0.0783	wR_2 = 0.0980	wR_2 = 0.0915	wR_2 = 0.2205	wR_2 = 0.1324
#CCDC	2243659	2243664	2243663	2243660	2243661	2243662

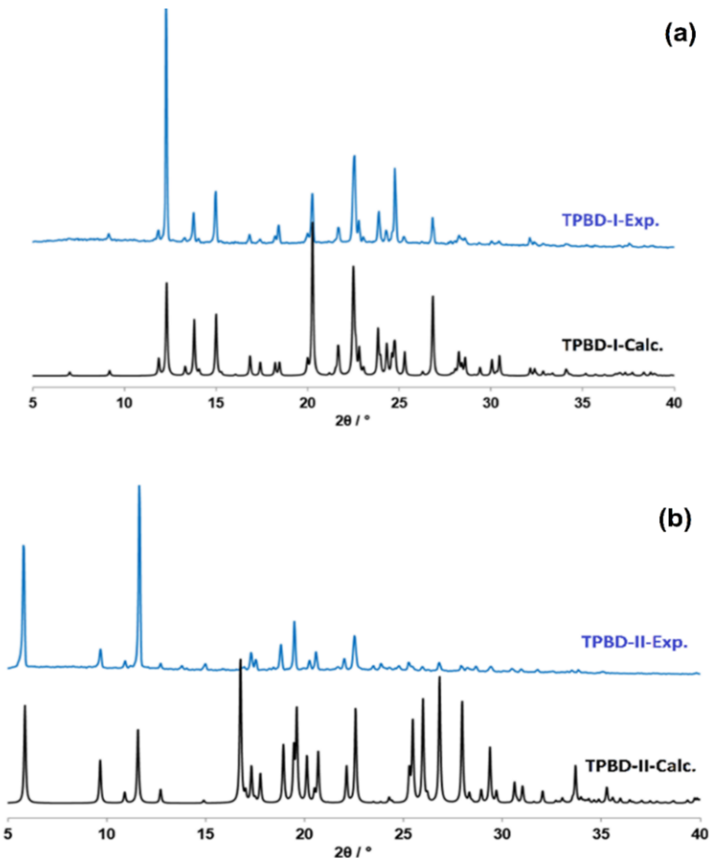

Figure S1: Calculated and experimental PXRD patterns of a) TPBD-I and b) TPBD-II

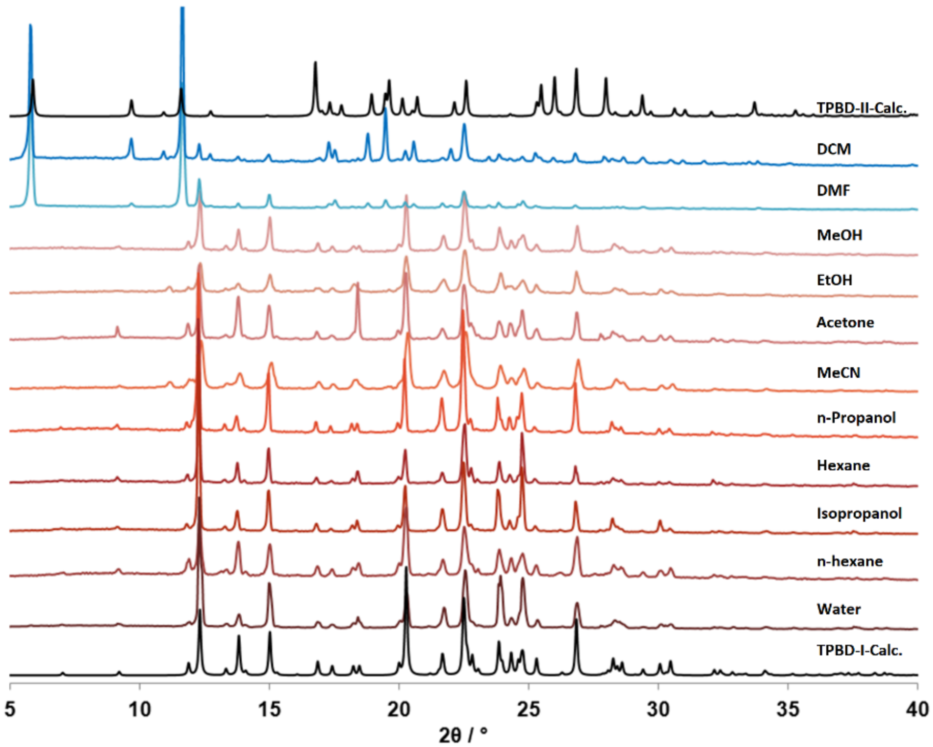

Figure S2. The PXRD patterns of the TPBD-I after soaking in different solvents at 80 °C. This figure illustrates results of the solvothermal experiments using sealed vials, where the temperature of 80 °C surpassed some solvent boiling points.

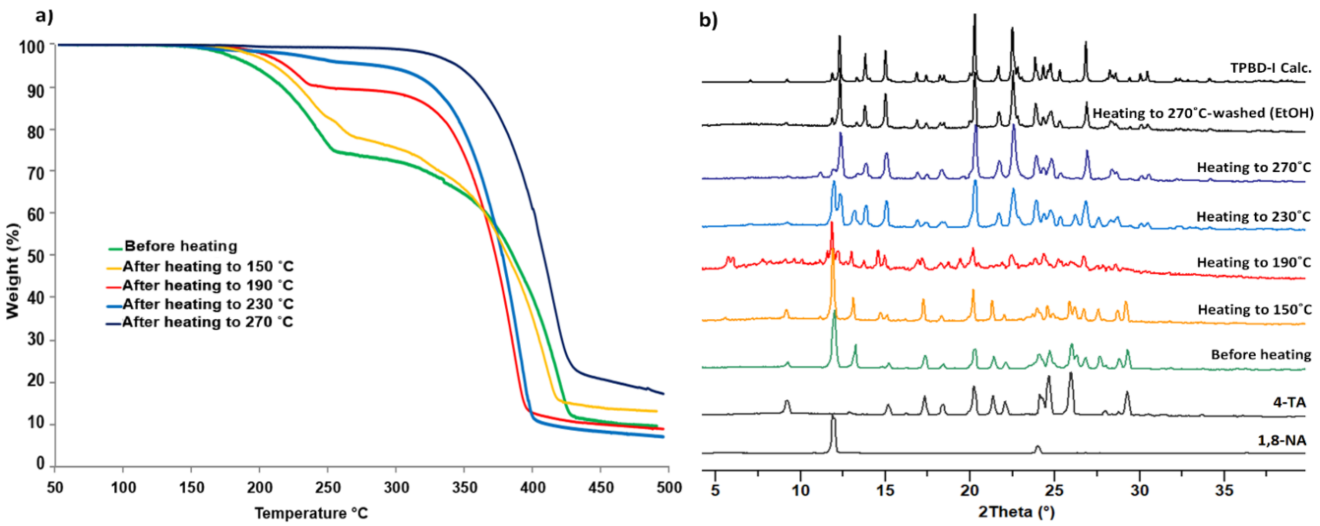

Figure S3. The optimization of the reaction conditions to synthesize TPBD-I, confirmed by (a) TGA, and (b) PXRD.

Table S4. Dihedral angle between the planes of A, B and C regions in different structures of TPBD.

Compounds	Angle of A and C	Angle of B and C	Angle of A and B
**TPBD-I**	30.86	61.98	33.65
**TPBD-II**	67.80	69.82	4.97
**TPBD-IIRT**	71.48	68.34	5.08
**TPBD-III**	51.09	65.24	14.60
**TPBD-IV**	41.34	73.15	32.44
**TPBD-PX**	82.46	87.08	5.20
	54.78	66.60	11.91

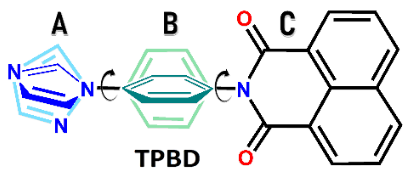

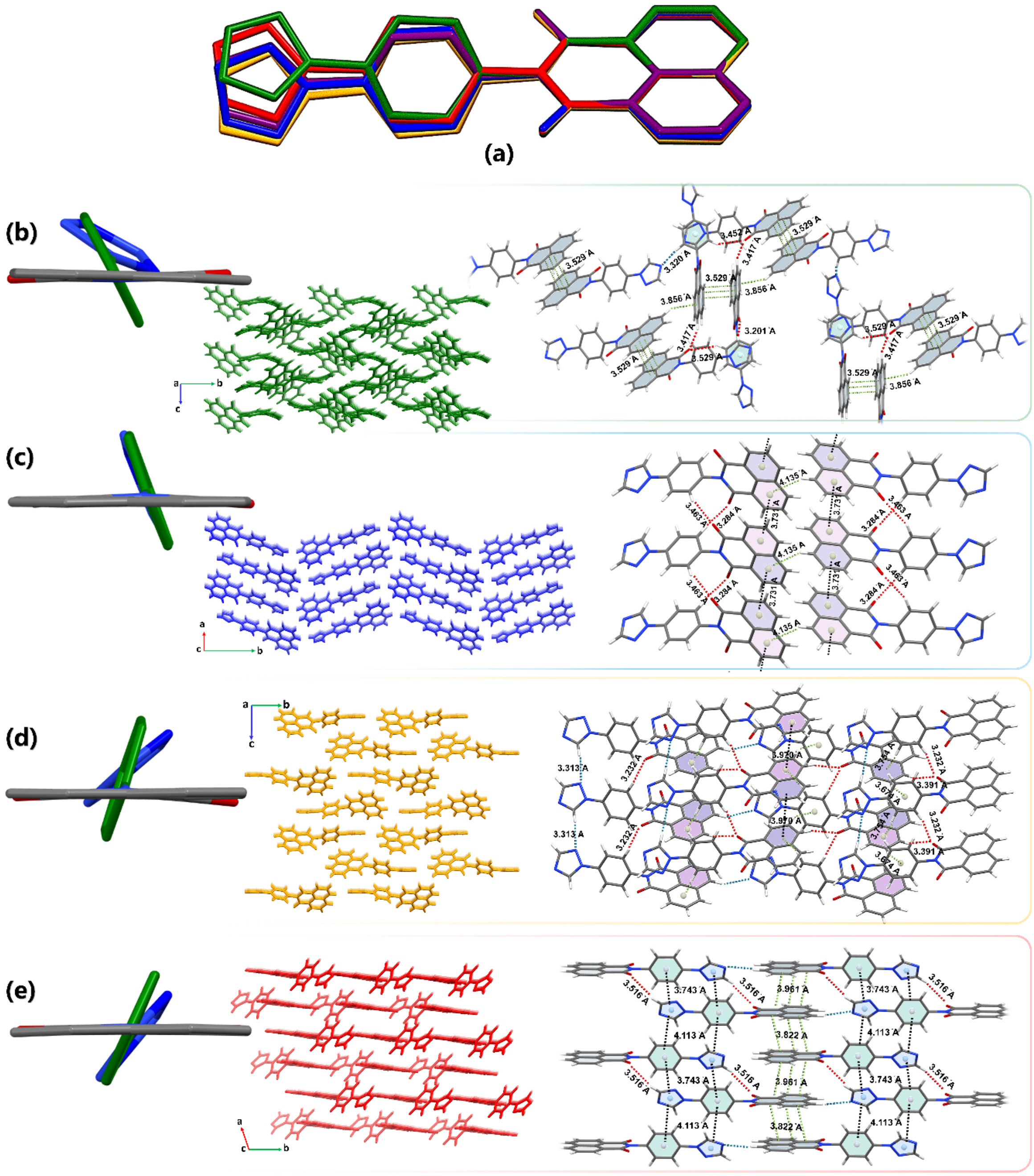

Figure S4. (a) Structural overlay of TPBD for TPBD-I: green, TPBD-II: blue, TPBD-III: orange, TPBD-IV: red, and TPBD-PX: purple, and relevant crystal packing of (b) TPBD-I, (c) TPBD-II, (d) TPBD-III, (e) TPBD-IV.

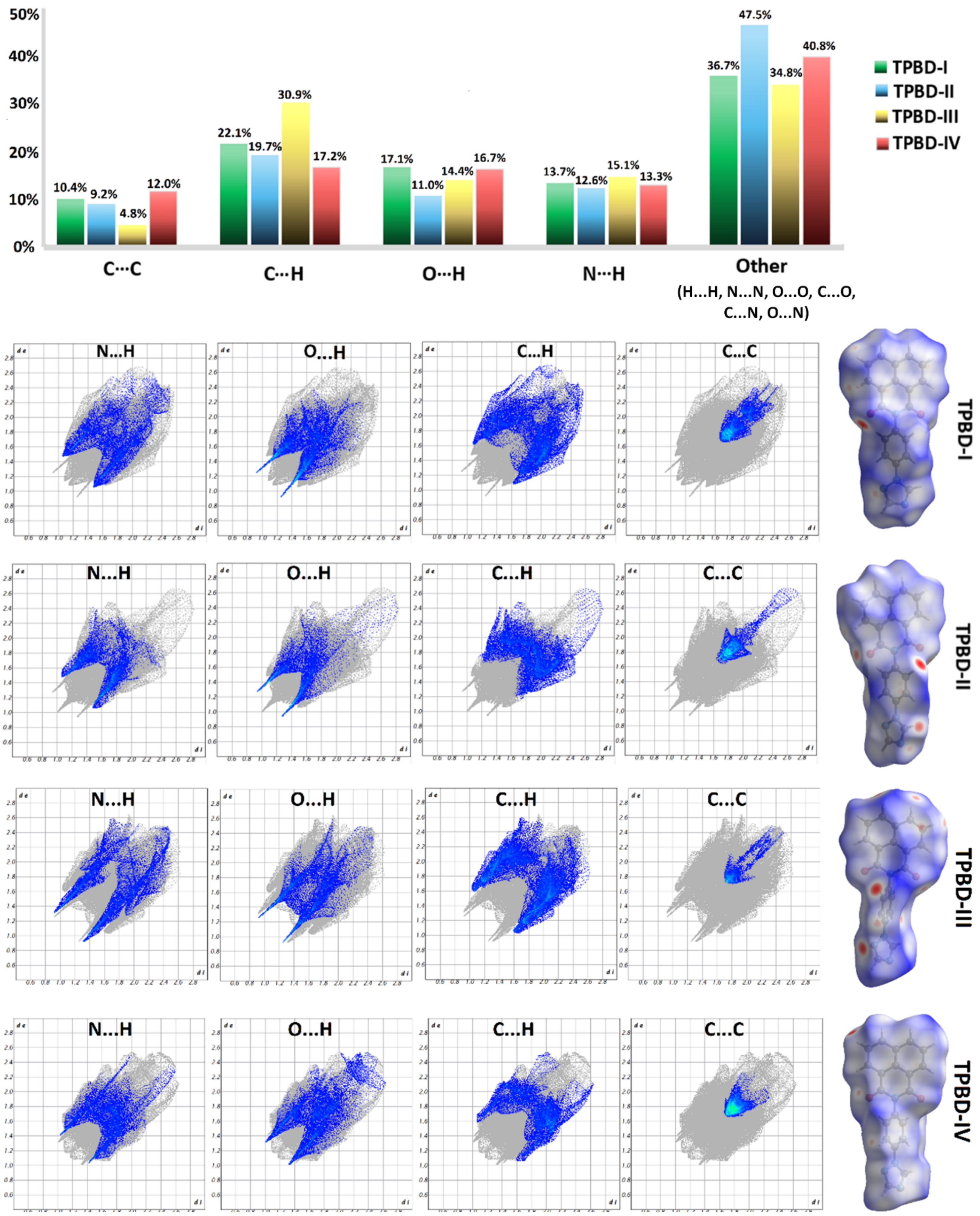

Figure S5. Relative contributions of various interactions to the Hirshfeld surface of TPBD-I-IV.

Computational studies

Table S5. Cell parameters for the investigated structures, total volume and applied k-point density during geometry optimization.

	a (Å)	b (Å)	c (Å)	α (º)	β (º)	γ (º)	Volume (Å^3)	k-point mesh	Small imaginary modes $\|\nu\|$ (replaced by 50 cm^{-1})
TPBD-I	12.7589	14.8509	8.0666	90.0	98.498	90.0	1511.69	3x2x4	-
TPBD-II	9.39	30.046	5.226	90.0	90.0	90.0	1474.42	3x1x8	-
TPBD-III	5.4652	15.9871	18.1854	90.0	90.0	90.0	1588.91	7x2x2	-
TPBD-IV	7.7712	9.4957	11.3201	100.379	103.77	106.383	750.48	4x3x3	-
$TPBD_8(PX)_4$	10.7208	31.4461	11.0004	90.0	95.562	90.0	3691.08	3x1x3	-48, -45, -25
$TPBD_8(MX)_4$	10.7208	31.4461	11.0004	90.0	95.562	90.0	3691.08	3x1x3	-75, -34, -20, -15
$TPBD_8(OX)_4$	10.7208	31.4461	11.0004	90.0	95.562	90.0	3691.08	3x1x3	-42, -22, -10, -7, -6
$TPBD_8(EB)_4$	10.7208	31.4461	11.0004	90.0	95.562	90.0	3691.08	3x1x3	-50, -40, -36, -22, -14, -10

Table S6. Optimized cell parameters (converged atomic forces < 0.005 eV/Å) of the investigated structures, total volume and applied k-point density during cell optimization.

	a (Å)	b (Å)	c (Å)	α (º)	β (º)	γ (º)	Volume (Å^3)	k-point mesh	Small imaginary modes $\|\nu\|$ (re-placed by 50 cm^{-1})
TPBD-I	12.9545	14.9824	8.6128	90.0	100.5572	90.0	1643.36	3x2x4	-
TPBD-II	9.8956	30.4930	5.3224	90.0	90.0	90.0	1606.02	3x1x8	-28, -16, -10
TPBD-III	5.6073	16.0410	18.7417	90.0	90.0	90.0	1685.77	7x2x2	-
TPBD-IV	8.4595	9.6741	11.2937	100.2488	105.1826	106.9283	820.37	4x3x3	-33
$TPBD_8(PX)_4$	10.9465	31.6407	11.5029	90.0	95.5967	90.0	3965.12	3x1x3	-50, -44, -24, -21, -15
$TPBD_8(MX)_4$	11.0400	31.7375	11.4656	90.0668	96.3172	89.9990	3992.94	3x1x3	-28, -23, -14
$TPBD_8(OX)_4$	11.1285	31.3732	11.5448	89.9885	96.8754	90.0004	4001.70	3x1x3	-49, -43, -33, -28, -25, -18
$TPBD_8(EB)_4$	11.0423	31.5497	11.4957	90.0364	95.3374	90.3051	3987.47	3x1x3	-44, -31, -25, -11

Table S7. Relative energy differences and Helmholtz free energy differences ($\Delta(\Delta F_T) = \Delta(\Delta E - T.\Delta S_{vib})$, T in Kelvin) at 25, 50, 100 and 150 °C (in kJ/mol$_{TPBD}$)

	Structures optimized with experimental cell-parameters					Structures with optimized cell-parameters				
	$\Delta(\Delta E)$	$\Delta(\Delta F_{298})$	$\Delta(\Delta F_{323})$	$\Delta(\Delta F_{373})$	$\Delta(\Delta F_{423})$	$\Delta(\Delta E)$	$\Delta(\Delta F_{298})$	$\Delta(\Delta F_{323})$	$\Delta(\Delta F_{373})$	$\Delta(\Delta F_{423})$
TPBD-I	2.6	10.7	11.2	12.2	13.2	1.3	0.2	0.0	0.0	0.0
TPBD-II	4.2	11.0	11.3	12.1	12.8	0.0	0.0	0.0	0.2	0.4
TPBD-III	0.0	0.0	0.0	0.0	0.0	3.4	3.0	3.0	3.0	3.2
TPBD-IV	8.3	16.9	17.7	19.3	21.0	4.4	3.8	3.9	4.5	5.1

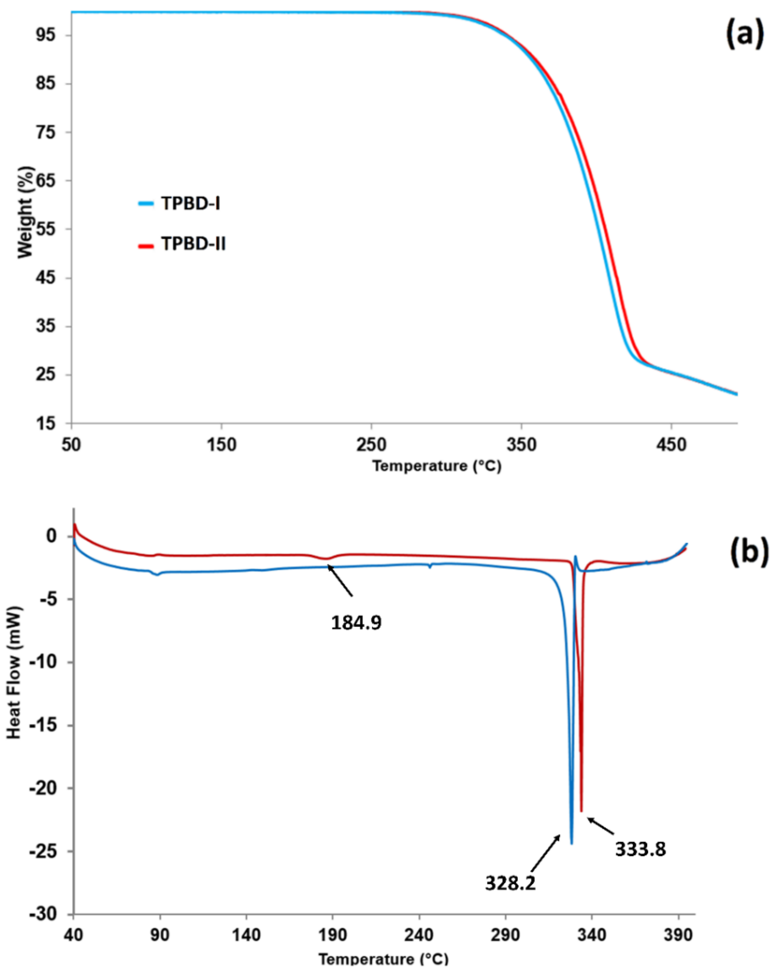

Figure S6. The comparison of (a) TGA and (b) DSC curves of the as-synthesized TPBD-I (blue) samples and TPBD-II (red) with a heat rate of 10 ̊C/min.

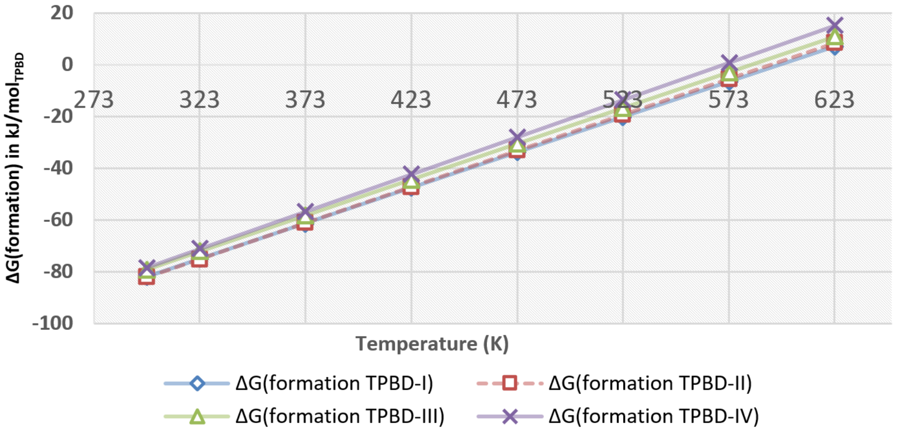

Figure S7. Gibbs free energy of formation from gas phase TPBD (p_{TPBD} = 1 bar) for the different TPBD polymorphs (calculation based on cell-optimized structures). ΔG(formation) becomes endergonic between 295 ºC and 333 ºC for all polymorphs. In this temperature range, the melting of the polymorphs can start as there is no driving force any longer towards crystallization, which agrees with DSC observations (Figure S6b). At higher temperatures >330 ºC, decomposition can occur via evaporation of TPBD molecules, which is in agreement weight loss observed in TGA (Figure S6a). Recrystallization processes (e.g. from one polymorph to other polymorphs during synthesis, melting, etc.) are expected to take place in the temperature range from 275 ºC to 325 ºC.

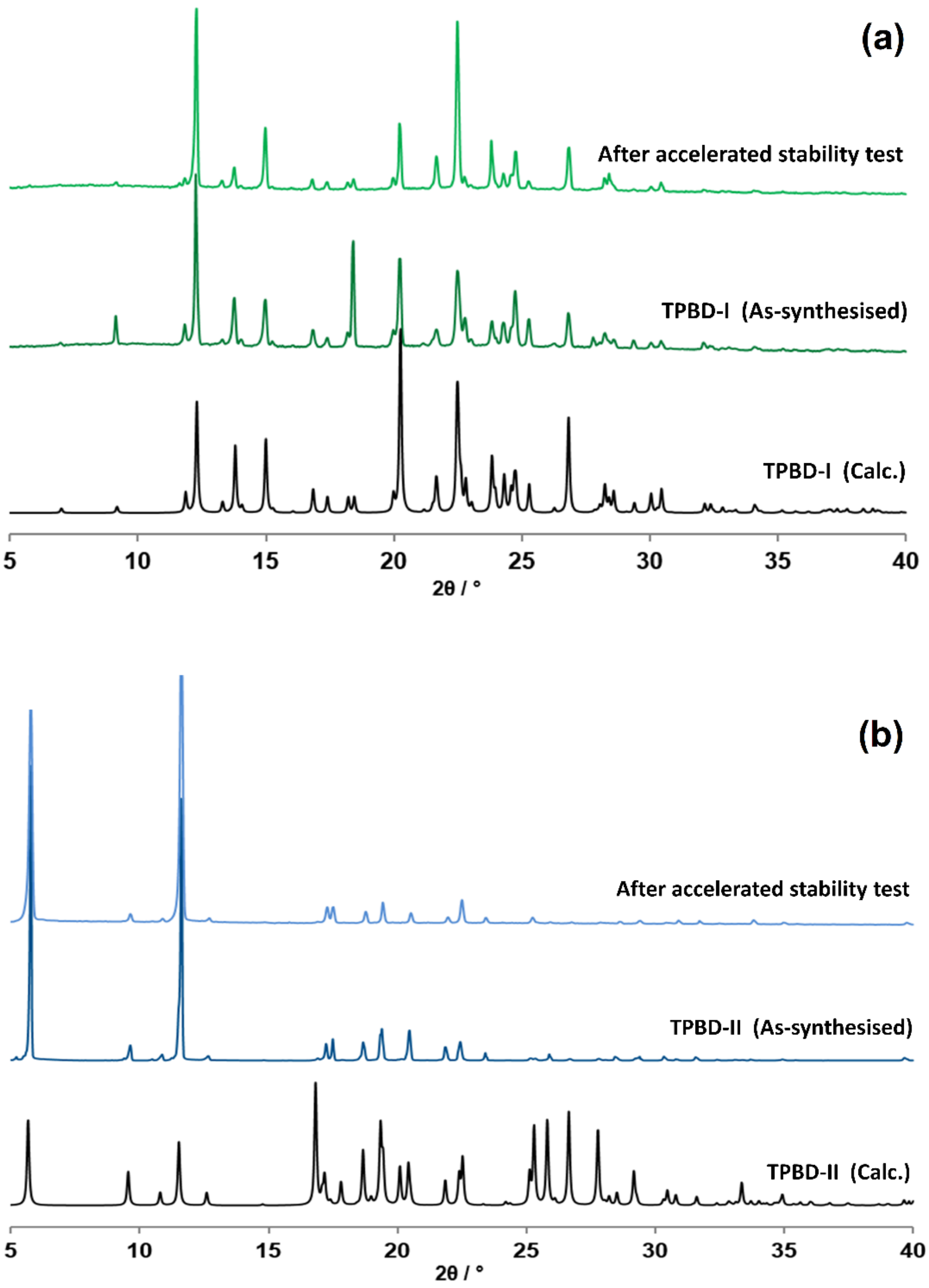

Figure S8: The PXRD patterns of TPBD-I and TPBD-II after 14 days of stability testing with restoration in a controlled humidity (70% RH/ 40 ± 2 °C chamber).

.

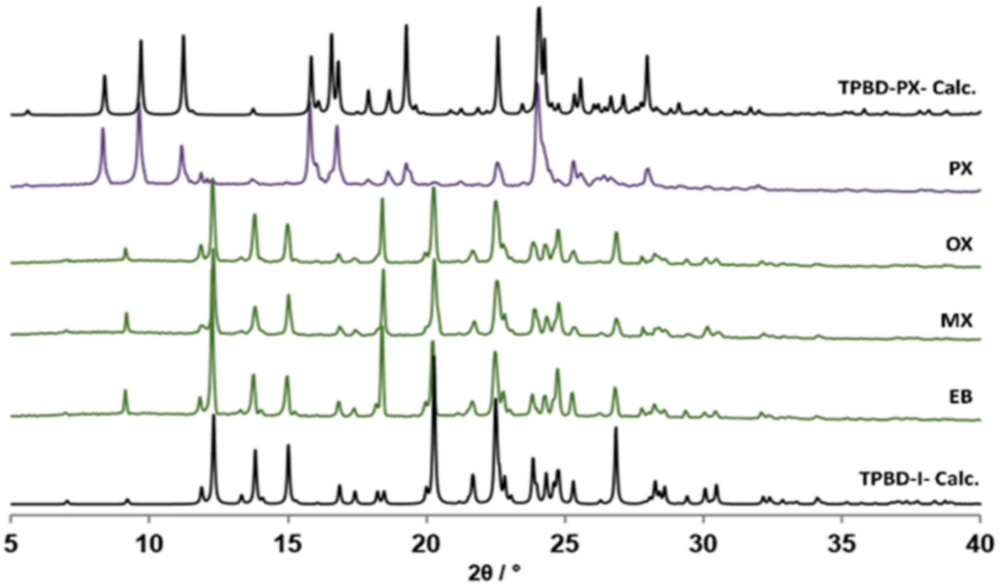

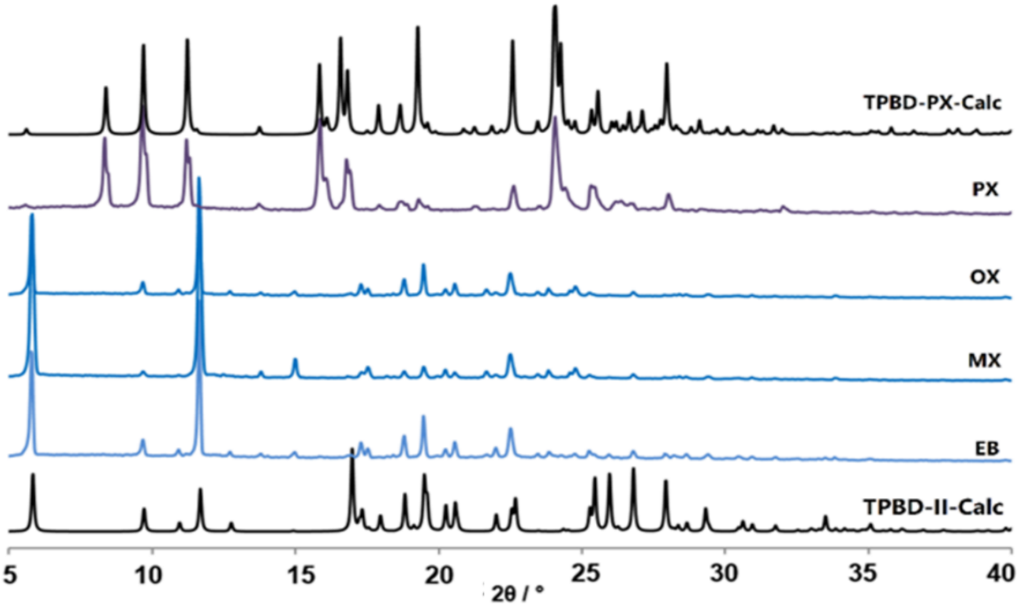

Figure S9: The PXRD patterns of the (Top) TPBD-I and (Bottom) TPBD-II after soaking in pure C8 solvents at 373K.

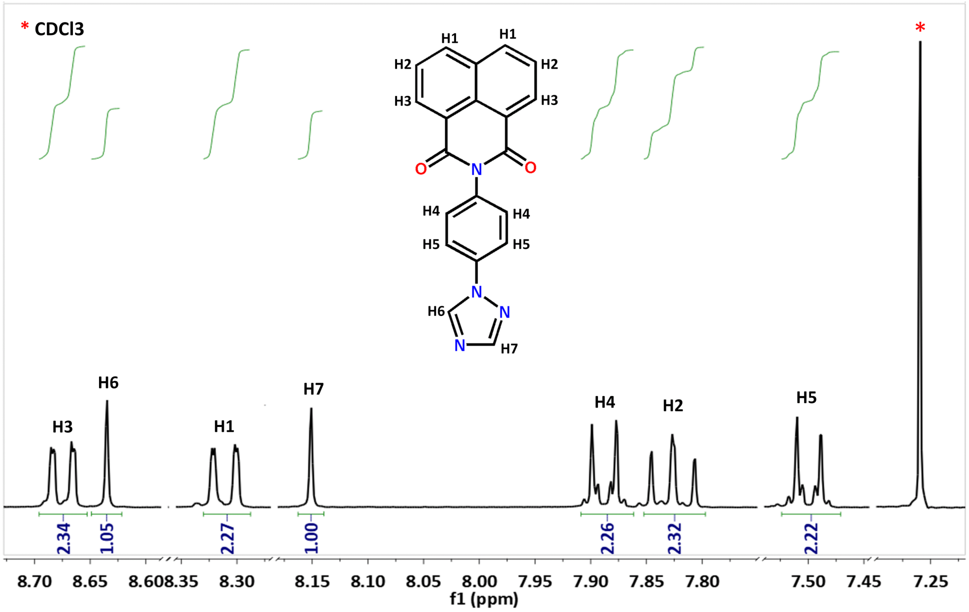

Figure S10.^{1}H NMR spectrum (400 MHz, CDCl3, 293 K) of TPBD-I.

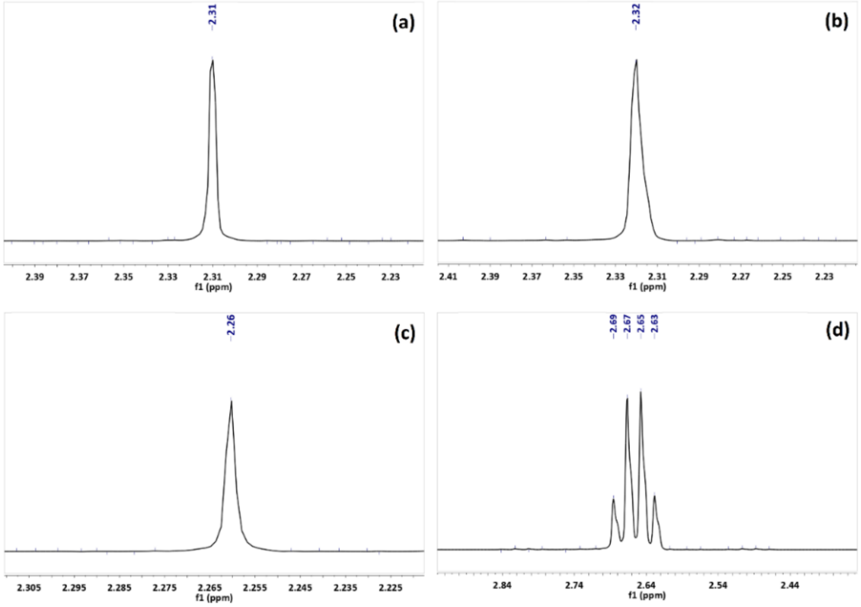

Figure S11. Magnified ^{1}H NMR spectrum for the methyl groups of pure (a) PX, (b) MX, (c) OX and (d) EB.

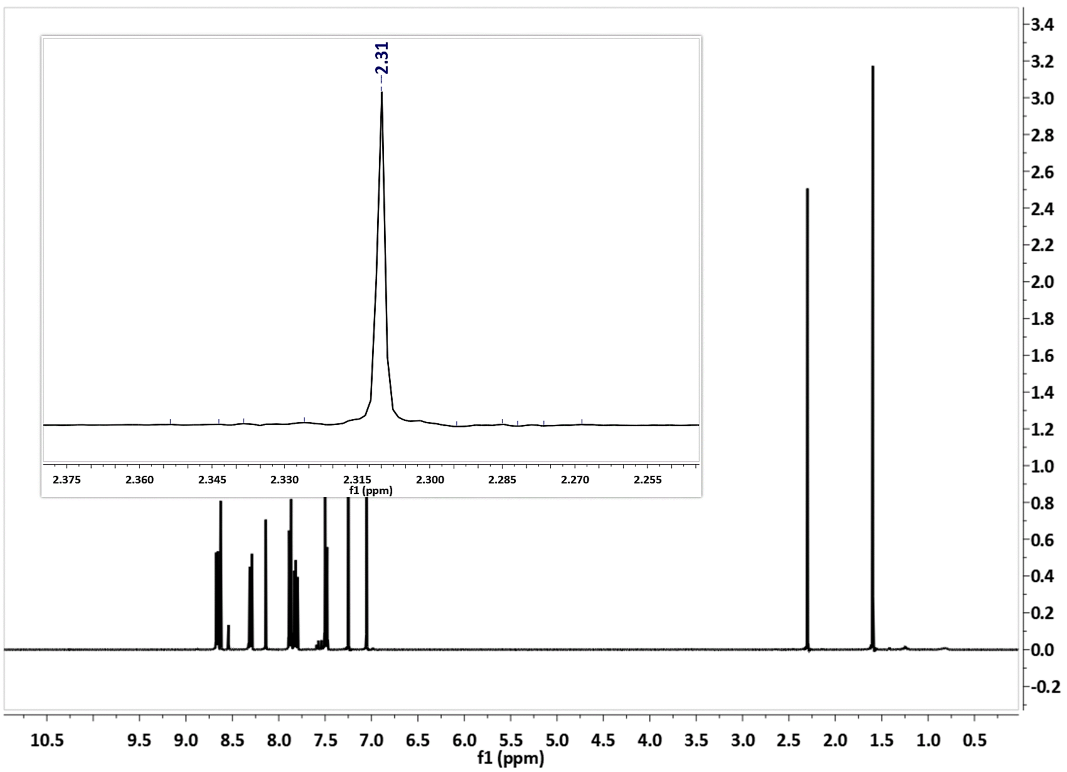

Figure S12. Relative uptake of PX by TPBD-I after being exposed to pure PX for 4 days, determined using ^{1}H NMR.

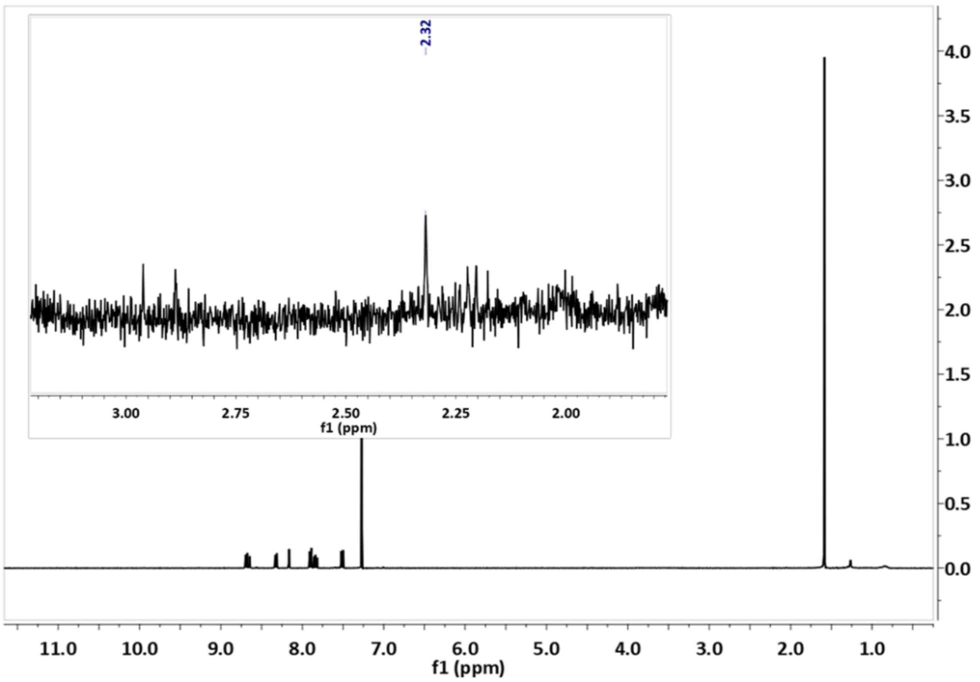

Figure S13. Relative uptake of MX by TPBD-I after being exposed to pure MX for 4 days, determined using ^{1}H NMR.

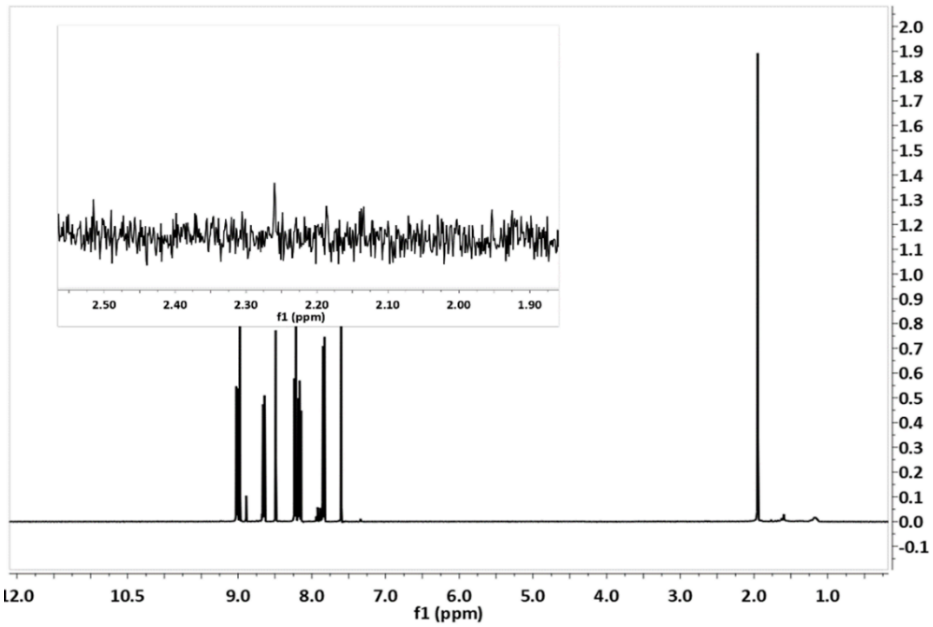

Figure S14. Relative uptake of OX by TPBD-I after being exposed to pure OX for 4 days, determined using ^{1}H NMR.

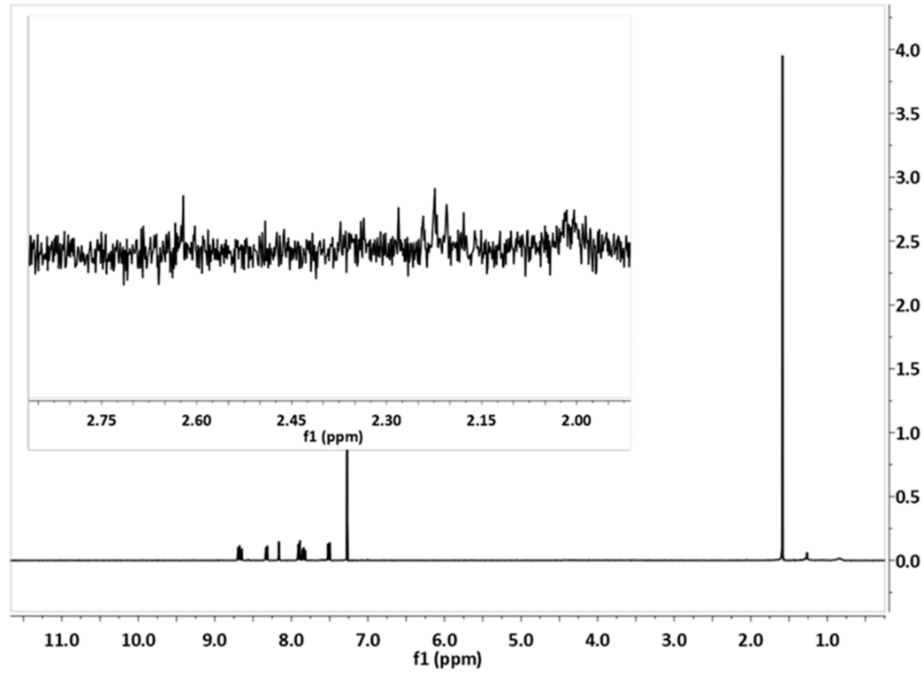

Figure S15. Relative uptake of EB by TPBD-I after being exposed to pure EB for 4 days, determined using ^{1}H NMR.

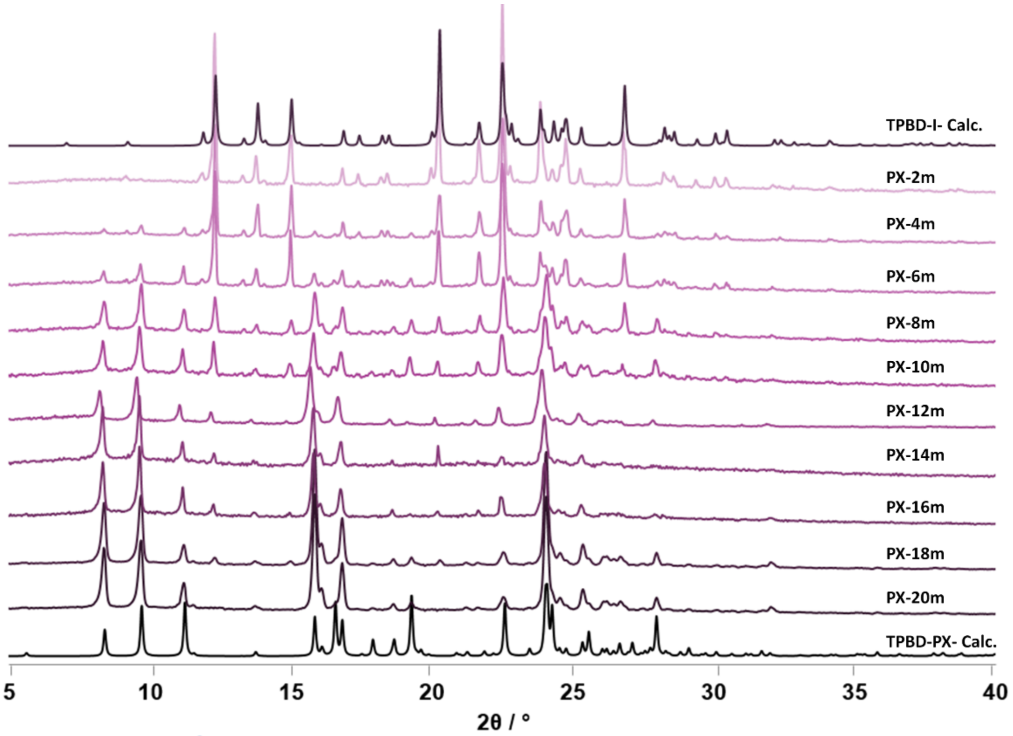

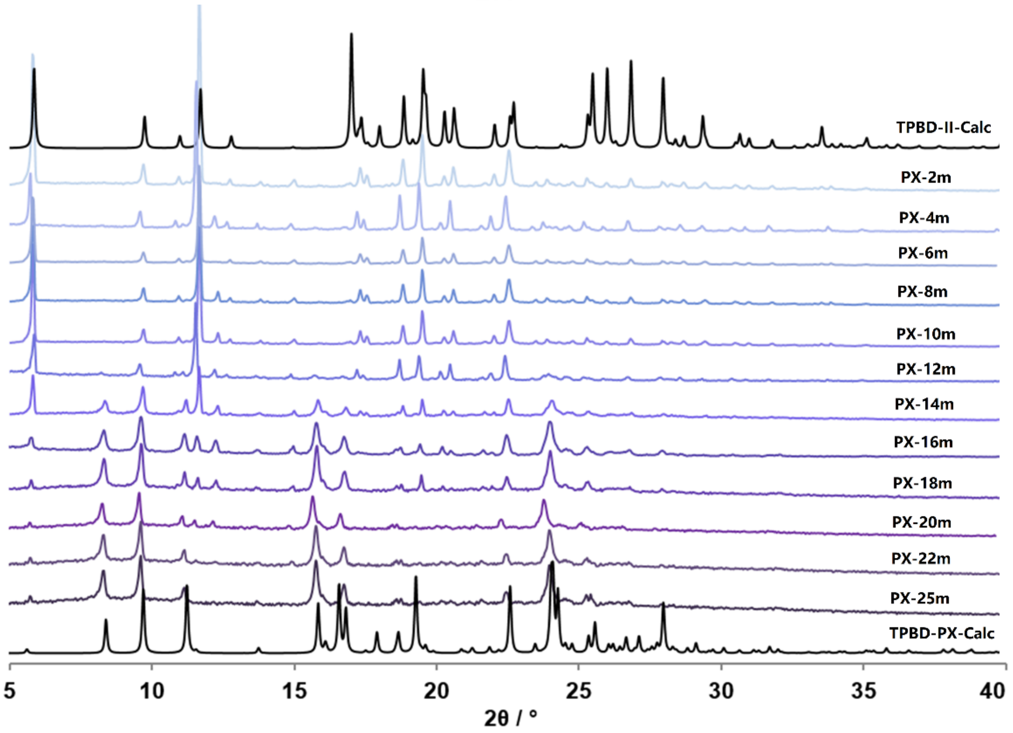

Figure S16. Experimental PXRD patterns of (Top) **TPBD-I** and (Bottom) **TPBD-II** along with the inclusion compounds formed after immersing them for 20 and 25 minutes, respectively, in pure PX isomer using a slurry-assisted immersion technique under ambient conditions.

Optical microscopy

Photomicrographs were recorded for single crystals of **TPBD-I** (Figure S17 and Figure S21) and **TPBD-II** (Figure S18 and Figure S22) during exposure to PX liquid at 25 °C. Two sets of experiments were conducted at two levels of magnification to identify the mechanism of PX inclusion into **TPBD-I** and **TPBD-II**.

For the first set of experiments, single crystals of **TPBD-I** (1.0 mg, Figure S17, Movie S1) and **TPBD-II** (20 mg, Figure S18, Movie S2) were placed in a 10 mL Pyrex beaker on an OPTIKA SZN-4 stereo zoom optical microscope and images were recorded on an Optikam Pro 3 digital camera at regular intervals using the software Optika Vision Pro (Version 2.7). The crystals were submerged in 5 mL of PX and the solution was replenished throughout the duration of the experiment ensuring that the overall volume did not drop substantially. Notably, the crystals changed from translucent to opaque in both experiments indicating the inclusion of PX had occurred. TGA (Figure S19) of the crystals after solvent exchange confirm that the PX was taken up stoichiometrically, and PXRD analysis (Figure S20) of these crystals before and after the optical experiments verify that the conversion to **TPBD-PX** was complete. From these images, it was clear that large crystals did not dissolve, but it was also clear that smaller crystals had grown on the larger crystals. Hence, it was challenging to determine the mechanism of inclusion from these images alone. To gain more clarity, we performed similar measurements on a more powerful microscope.

For the second set of experiments, single crystals of **TPBD-I** (1.5 mg) and **TPBD-II** (1.5 mg) were placed in a borosilicate petri dish with internal diameter 29.0 mm and height 9.0 mm on a Carl Zeiss Axio Imager 2 stereo zoom optical microscope and images were recorded on a Carl Zeiss AxioCam at regular intervals using the software Carl Zeiss AxioVision (version 4.8.2.0). The crystals were submerged in 6 mL of PX and sealed with a watch glass such that the PX liquid was in contact with the watch glass. Rapid crystal growth of **TPBD-PX** onto larger single crystals of **TPBD-I** was observed shortly after PX exposure (Figure S21, Movie S3). The larger **TPBD-I** crystals remained mostly unchanged, but several of the smaller crystals dissolved. For **TPBD-II**, some of the larger crystals did not dissolve, although dissolution striations were observed (Figure S22, Movie S4). Similar to **TPBD-I**, many of the smaller **TPBD-II** crystals dissolved.

X-ray diffraction analysis of single crystals of TPBD-I to **TPBD-PX after PX exposure**

To determine whether larger crystals that did not dissolve in PX retain their apohost form or adsorb PX, diffraction patterns of single crystals were evaluated after PX exposure. A single crystal of **TPBD-I** (148 × 156 × 183 μm^3) was fixed onto a glass fiber using clear epoxy (Figure S23a), such that the epoxy covered the least surface area possible, and submerged in liquid PX for 15 h. The sample remained intact after PX exposure (Figure S23b), however, the emergence of Debye rings in the diffraction frames and the disappearance of well-defined Bragg peaks indicated that the crystal had lost its single crystallinity and that the crystal had possibly converted to the PX included phase (Figure S23c). The absence of well-defined Bragg peaks confirms that the conversion had not only occurred at the surface of the crystal. The d-spacing values of the crystal after PX exposure were determined by integrating the Debye rings in the diffraction frame using the APEX4[1] software. Comparing these with diffractograms calculated from SCXRD data of **TPBD-I** and **TPBD-PX** (Figure S23d) indicated that the crystal had converted to the structure **TPBD-PX** without significant loss of its original morphology: The peaks centered at d = 11.0, 9.5 and 8.2 Å determined for the single crystal correspond to those at d = 10.4, 9.0 and 7.9 Å in the **TPBD-PX** diffractogram. The absence of peaks at ca 7.4 and 7.0 Å, which are present in the **TPBD-I** diffractogram, indicate that conversion from **TPBD-I** to **TPBD-PX** was complete. The shift in peak position, which is observed over the entire d-spacing range, could have resulted from slight misalignment of the crystal with respect to the incident beam and detector. Similar experiments were carried out for single crystals of **TPBD-II**, however, the crystals dissolved in all cases.

Captions for Supplementary Movies

Movie S1: Time-lapse photomicroscopy of single crystals of **TPBD-I** (1.0 mg) following immersion in liquid PX at 25 °C.

Movie S2: Time-lapse photomicroscopy of single crystals of **TPBD-II** (20.0 mg) following immersion in liquid PX at 25 °C.

Movie S3: Time-lapse photomicroscopy of single crystals of **TPBD-I** (1.5 mg) following immersion in liquid PX at 25 °C.

Movie S4: Time-lapse photomicroscopy of single crystals of **TPBD-II** (1.5 mg) following immersion in liquid PX at 25 °C.

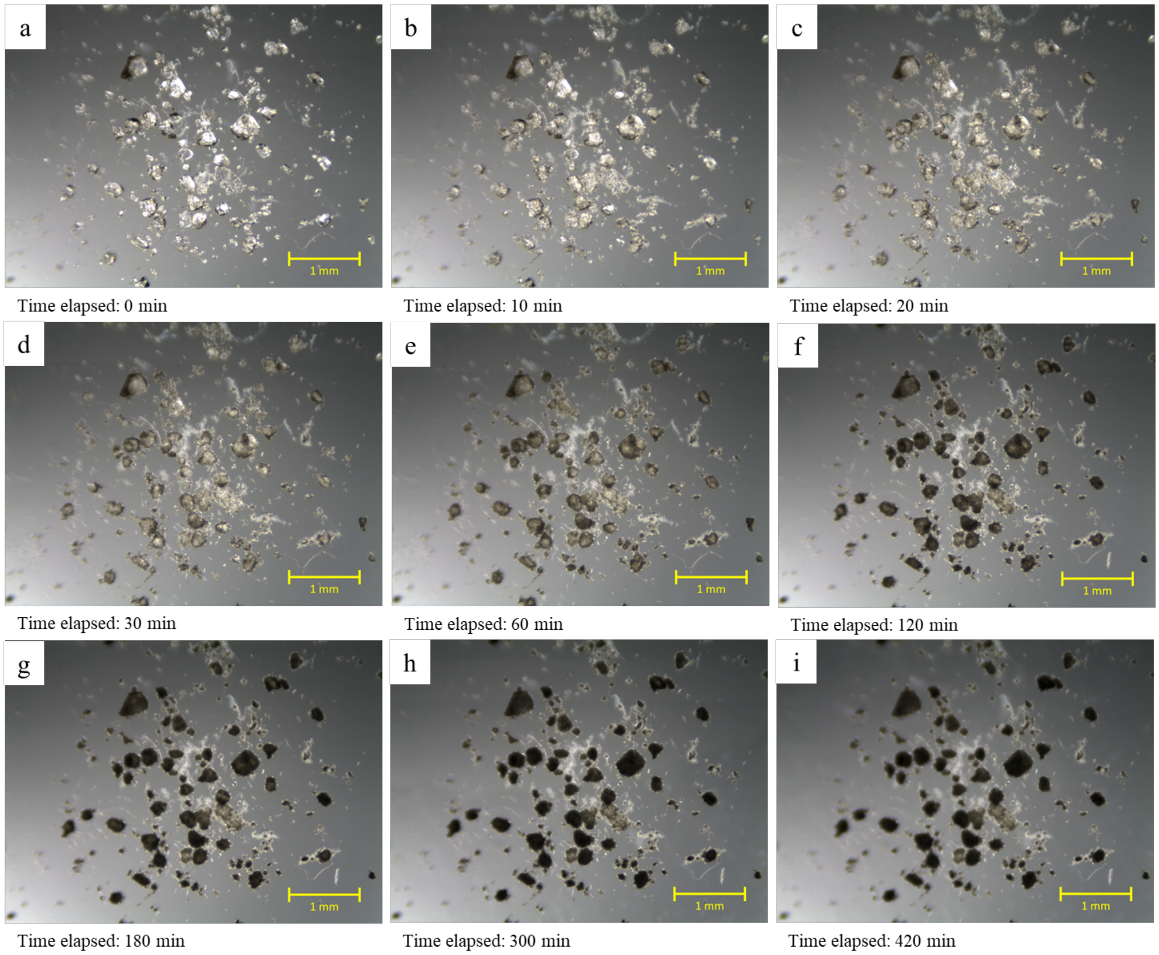

Figure S17. Time-lapse photomicrographs of **TPBD-I** immersed in PX at 25 °C after (a) 0 min, (b) 10 min, (c) 20 min, (d) 30 min, (e) 60 min, (f) 120 min, (g) 180 min, (h) 300 min and (i) 420 min.

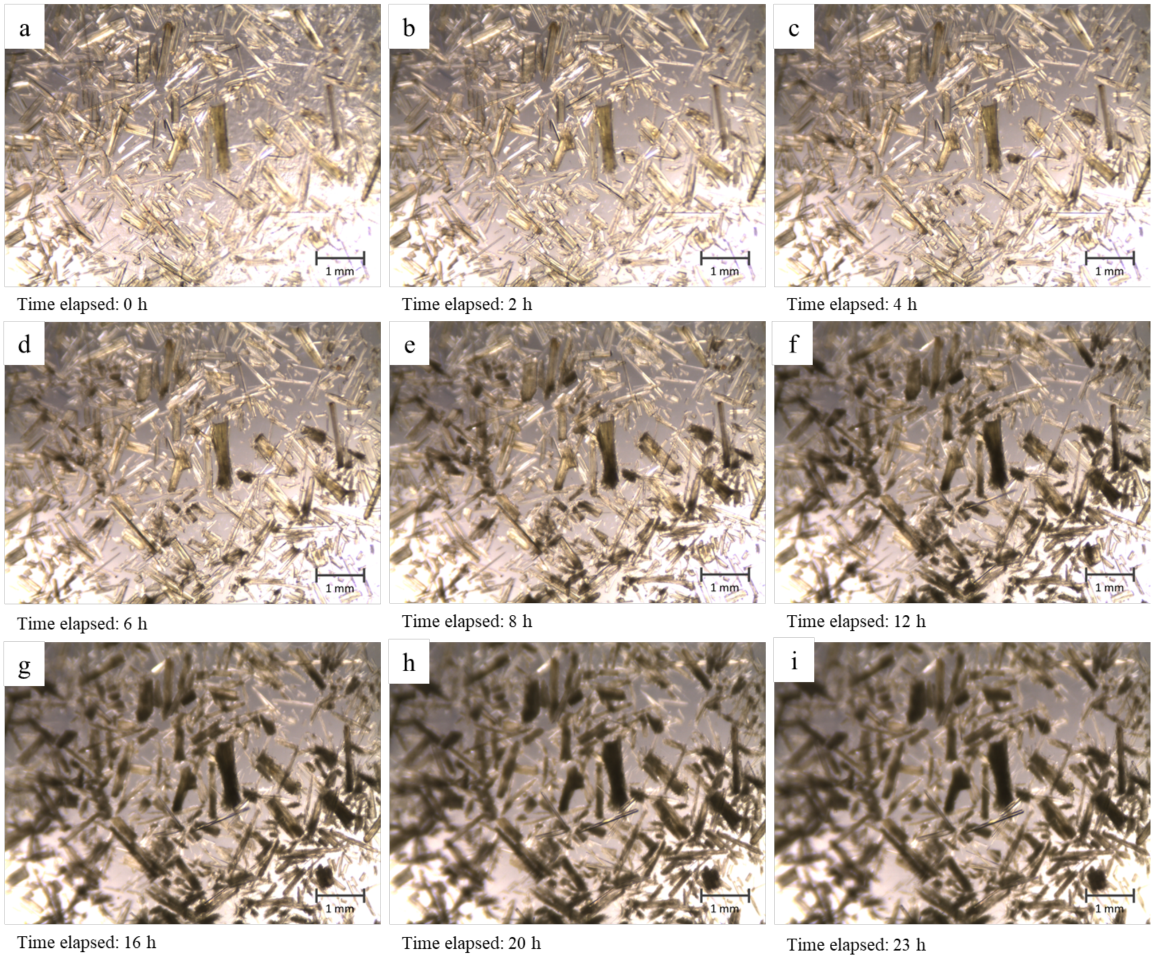

Figure S18. Time-lapse photomicrographs of **TPBD-II** immersed in PX at 25 °C after (a) 0 h, (b) 2 h, (c) 4 h, (d) 6 h, (e) 8 h, (f) 12 h, (g) 16 h, (h) 20 h and (i) 23 h.

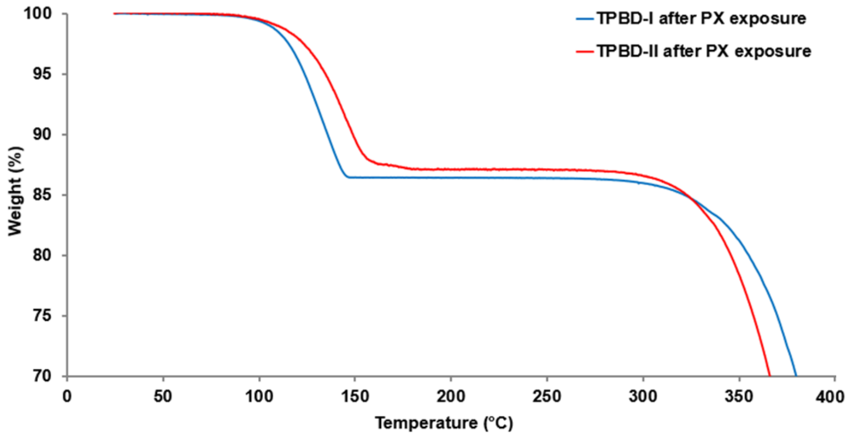

Figure S19. TGA thermograms of **TPBD-I** and **TPBD-II** after PX exposure during photomicroscope experiments.

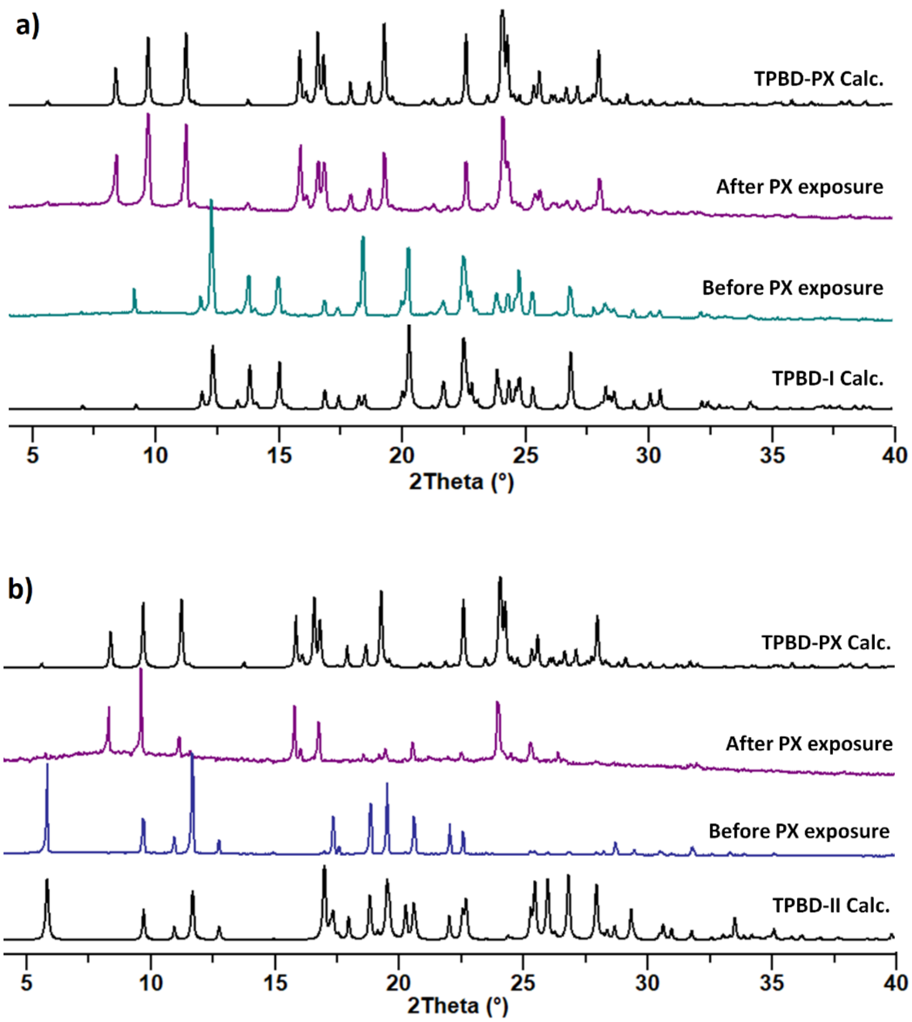

Figure S20. Experimental PXRD patterns of (a) **TPBD-I** and (b) **TPBD-II** before and after PX exposure during photomicroscope experiments.

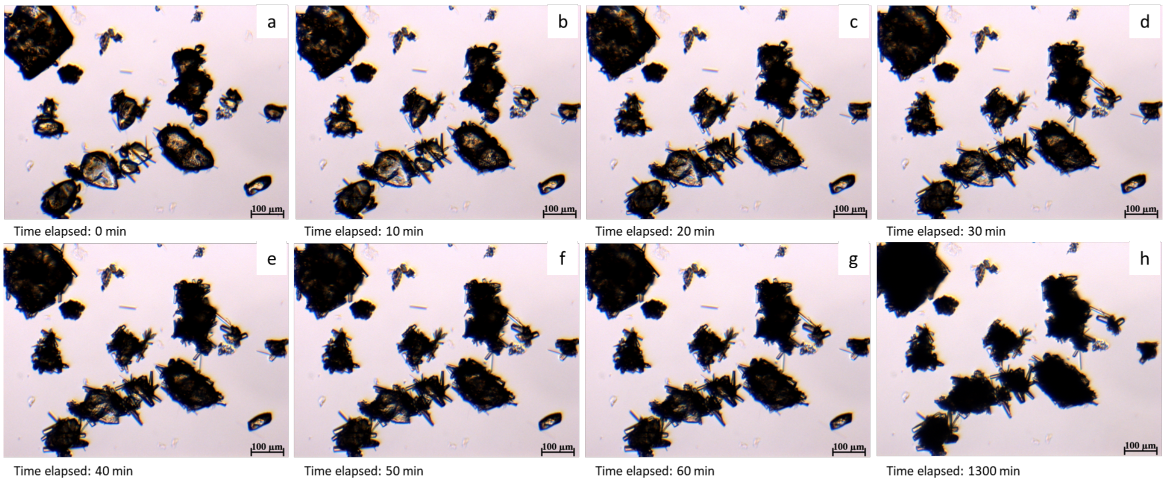

Figure S21. Time-lapse photomicrographs of **TPBD-I** immersed in PX at 25 °C after (a) 0 min, (b) 10 min, (c) 20 min, (d) 30 min, (e) 40 min, (f) 50 min, (g) 60 min, (h) 1300 min.

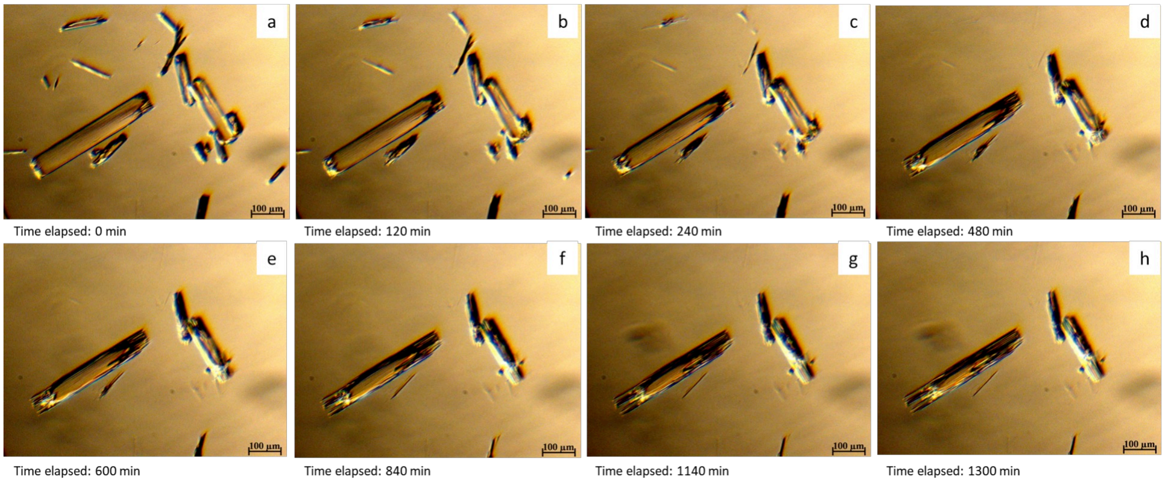

Figure S22. Time-lapse photomicrographs of **TPBD-II** immersed in PX at 25 °C after (a) 0 min, (b) 120 min, (c) 240 min, (d) 480 min, (e) 600 min, (f) 840 min, (g) 1140 min and (h) 1300 min.

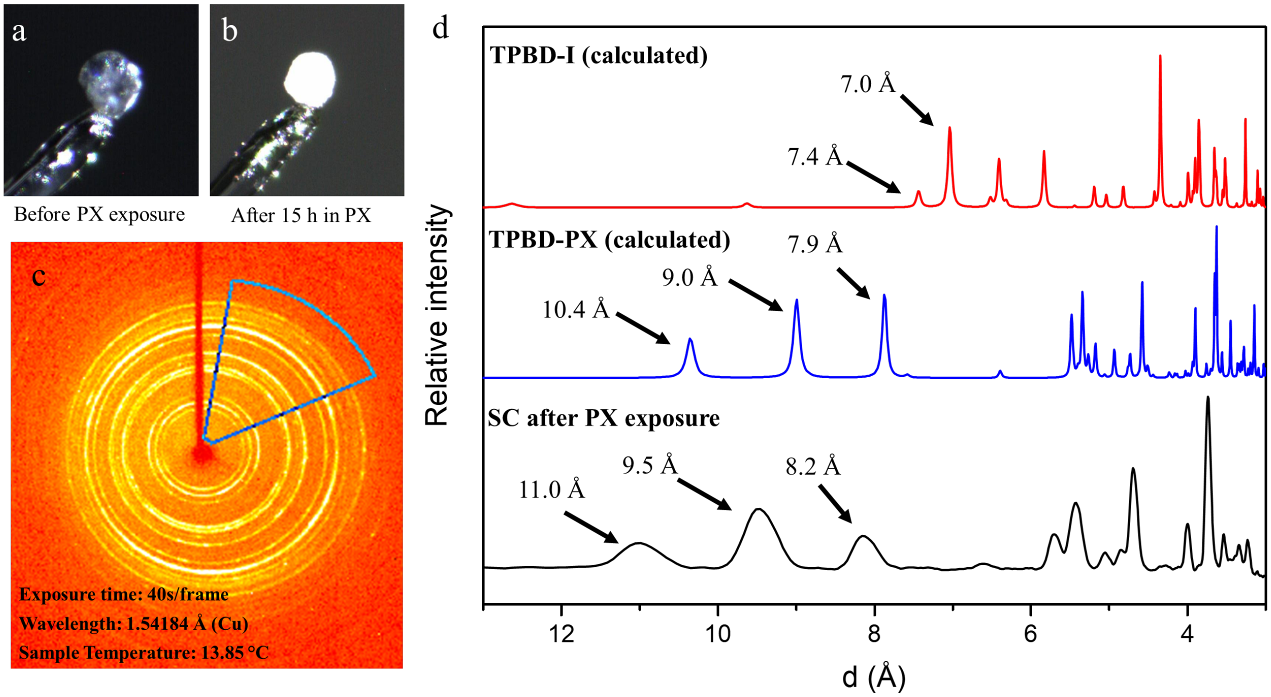

Figure S23. Photomicrographs of a single crystal of **TPBD-I** (148 × 156 × 183 μm^3) glued to a glass fiber (a) before and (b) after 15 h of PX exposure. (c) Diffraction frame of the single crystal after 15 h of PX exposure showing concentric Debye rings that indicate a loss in single crystallinity. (d) Powder X-ray diffractograms of the **TPBD-I** single crystal (SC) after 15 h PX exposure (black) and **TPBD-PX** (blue) and **TPBD-I** (red) calculated from SCXRD data (Table S3). The d-spacing values of key peaks are indicated with arrows.

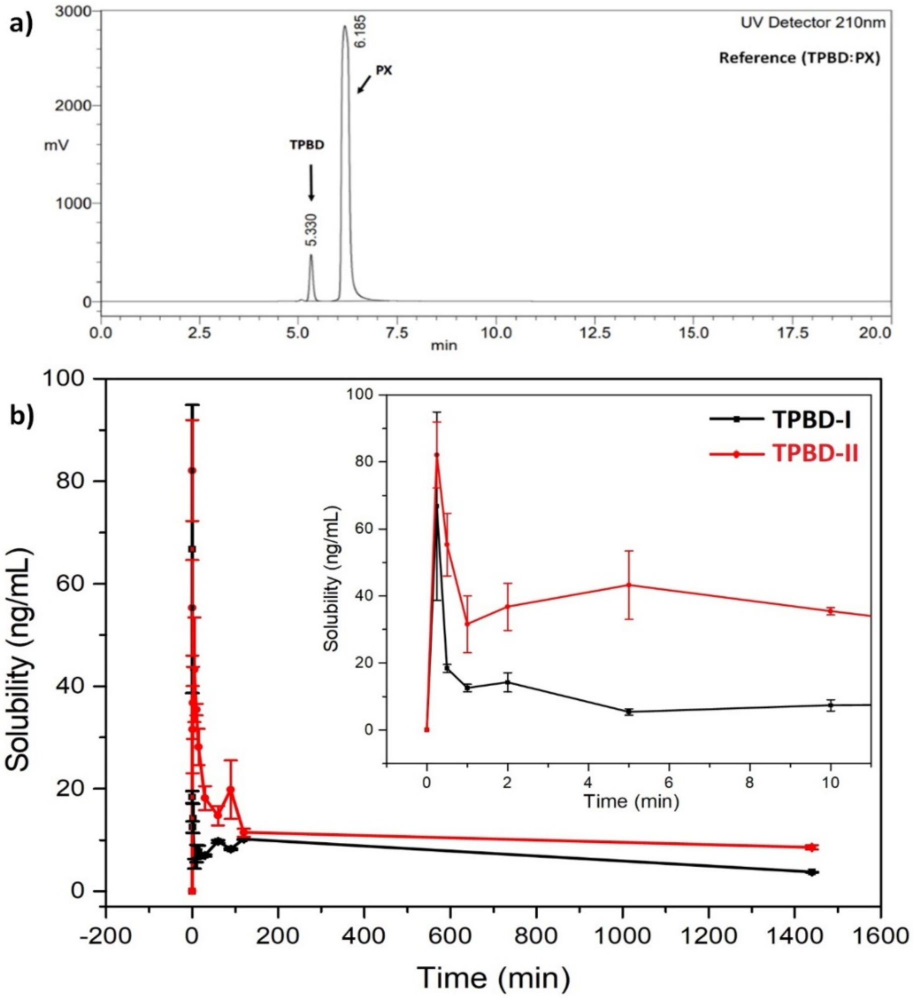

Figure S24. Reversed -phase HPLC chromatograms depicting TPBD: (a) in a reference solution containing TPBD and PX eluting at 5.330 and 6.185 minutes, respectively, and (b) mean TPBD concentrations versus time profiles in PX solution for TPBD-I (black square) and TPBD-II (red circle) stirred at 100 rpm at 25± 3°C. Each point represents the mean ± SD (n = 3).

Table S8. Stability Data for TPBD-I and TPBD-II

Time (minutes)	TPBD-I	TPBD-II
**0.25**	66.77± 28.12	82.10±17.46
**0.5**	18.41±1.19	55.31±16.57
**1**	12.53±1.12	31.57±15.04
**2**	14.19±2.86	36.78±12.46
**5**	5.36±0.92	43.24±18.11
**10**	7.36±1.65	35.45±1.99
**15**	7.68±1.25	28.18±6.31
**30**	6.99±0.18	18.19±4.15
**60**	9.71±0.28	14.76±3.36
**9**	8.22±0.16	19.84±10.08
**120**	10.14±0.10	11.46±1.37
**1440**	3.74±0.05	8.55±0.72

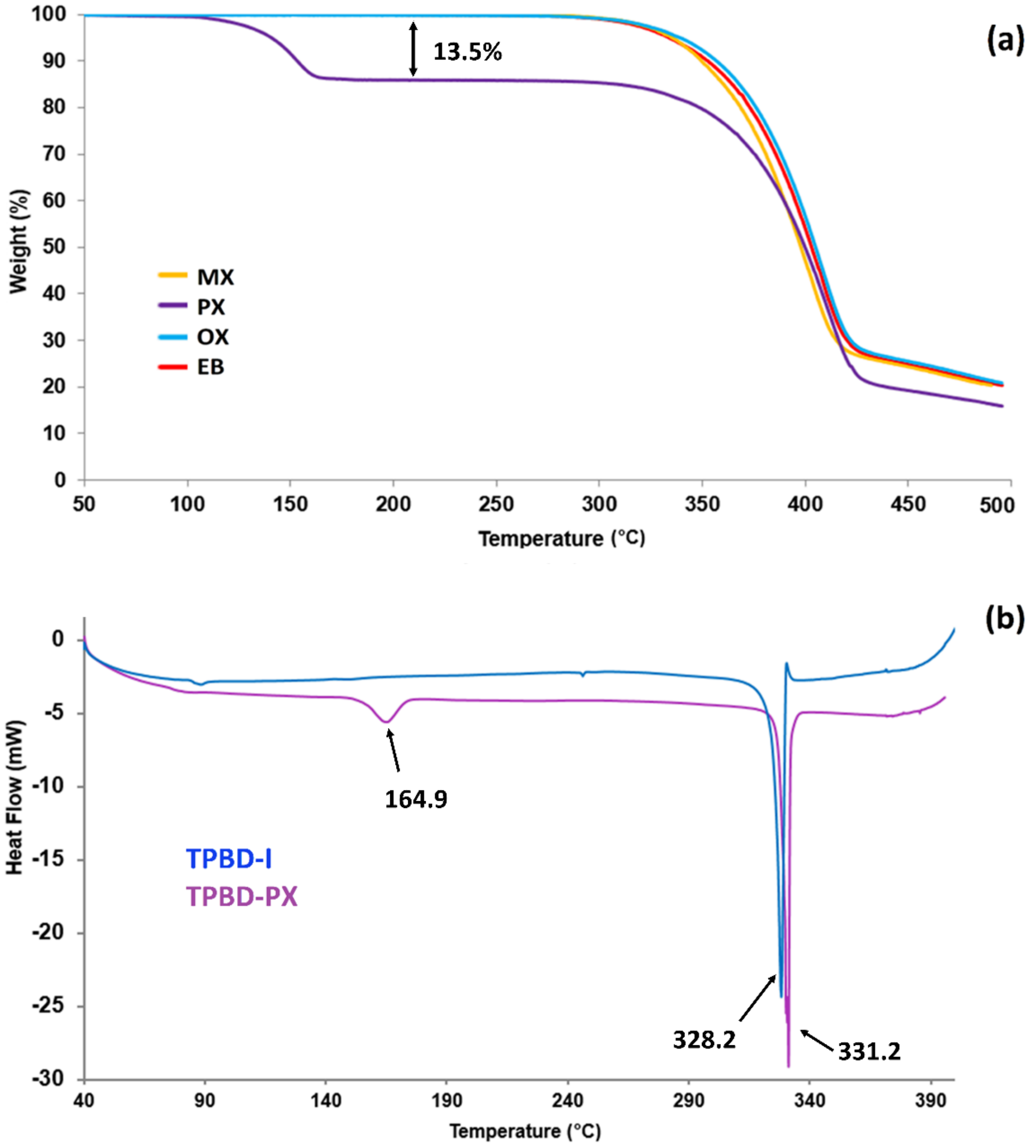

Figure S25. (a) Comparison of thermograms after immersing TPBD-I in pure OX, MX, PX and EB for at least 4 days at RT; (b) DSC curves of the TPBD-I and TPBD-PX.

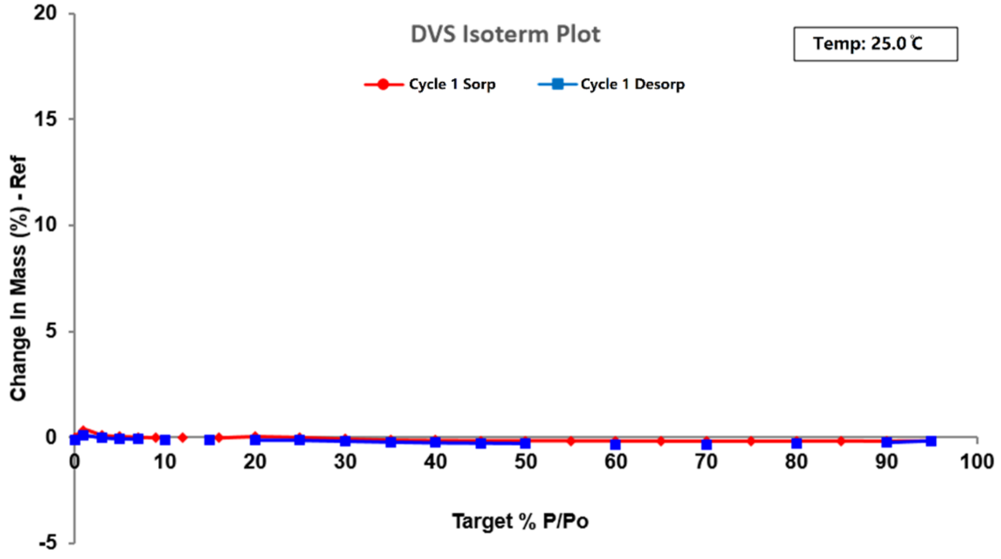

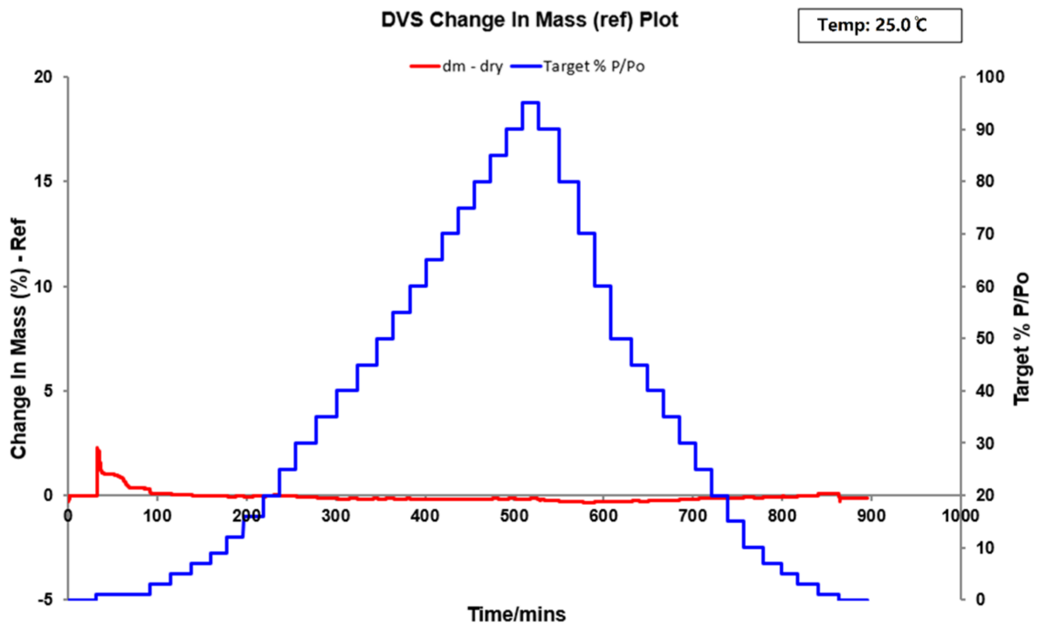

Figure S26. PX vapor sorption on TPBD-I at 25ºC, relative pressure range: 0 – 90%.

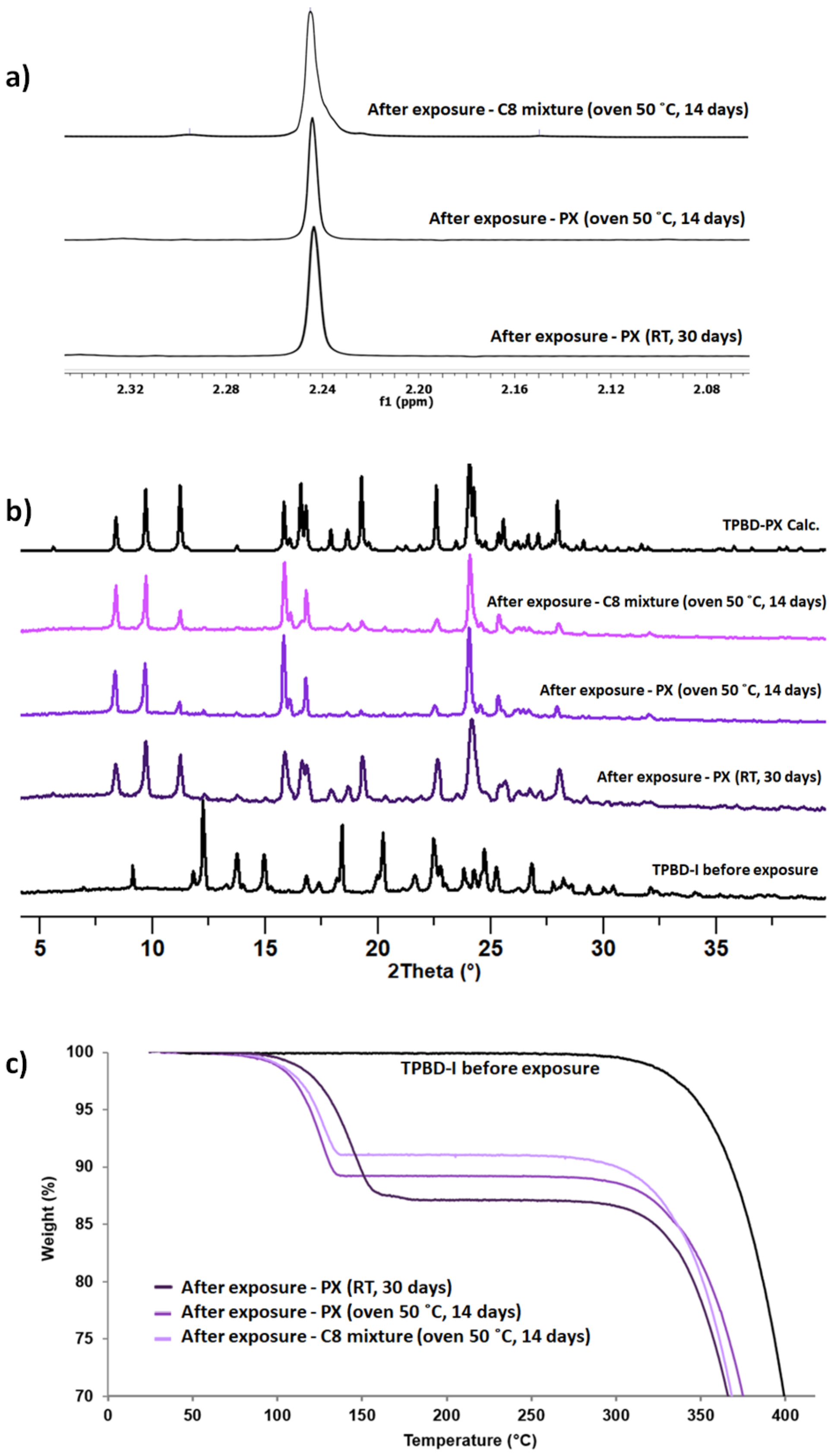

Figure S27. PX vapor sorption on TPBD-I at 25 and 50 ºC, determined using (a) ^{1}H NMR (solvent: DMSO), (b) PXRD, and (c) TGA.

Uptake selectivity calculation for liquid phase:

Approximately 34 mg (0.1 mmol) of **TPBD-I** were immersed and allowed to equilibrate for 4 days at RT in a 20 mL vial containing 1 mL of equimolar mixtures of C8 aromatics. Figure S49 of PXRD and Figure S50 of TGA show that **TPBD-I** completely uptake the PX. In order to remove xylenes that had adhered to the surface of the samples, the saturated samples were filtered and air-dried for approximately one hour at room temperature (roughly 25±3 °C). Mole fractions in guest-loaded phase were determined by ^{1}H NMR spectra.

The selectivity coefficient[13] of component i relative to component j is defined as:

$$S_{ij} = \frac{x_i}{x_j}\frac{y_j}{y_i}$$

where x_i and x_j are the mole fractions of components i and j in the guest-loaded phase, and y_i and y_j are the mole fractions of components i and j in the liquid phase. The following equation was used to obtain the uptake selectivity for quaternary mixtures ($S_{PX/OME}$)

$$S_{PX/OME} = \left(\frac{x_{PX}}{x_{OX}+x_{MX}+x_{EB}} / \frac{y_{PX}}{y_{OX}+y_{MX}+y_{EB}}\right)$$

The selectivity coefficient of **TPBD-I** for PX over various C8 isomers, as determined by ^{1}H NMR spectra, is listed in Tables 1 and S8.

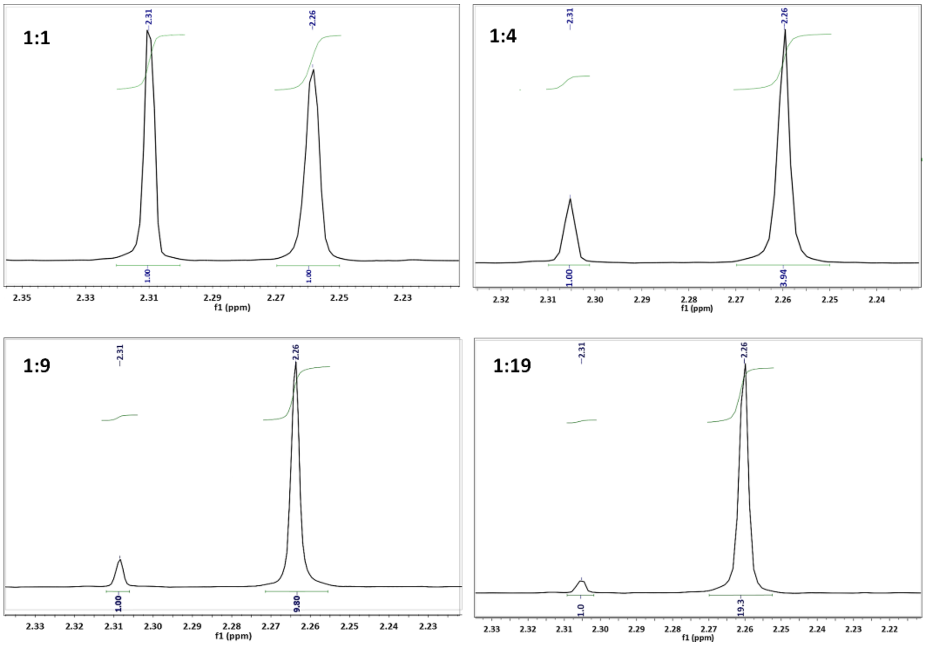

Figure S28. Methyl group region from the ^{1}H NMR spectrum of binary mixture feed of PX/OX with different ratios (1:1, 1:4, 1:9 and 1:19 mol:mol PX and OX).

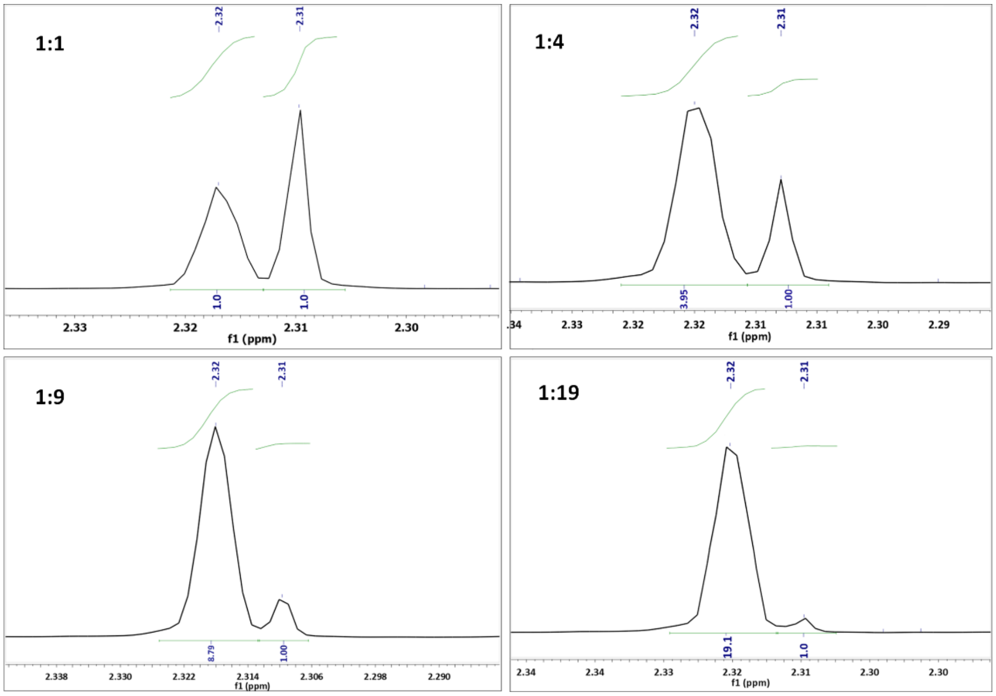

Figure S29. Methyl group region from the ^{1}H NMR spectrum of binary mixture feed of PX/MX with different ratios (1:1, 1:4, 1:9 and 1:19 mol:mol PX and MX).

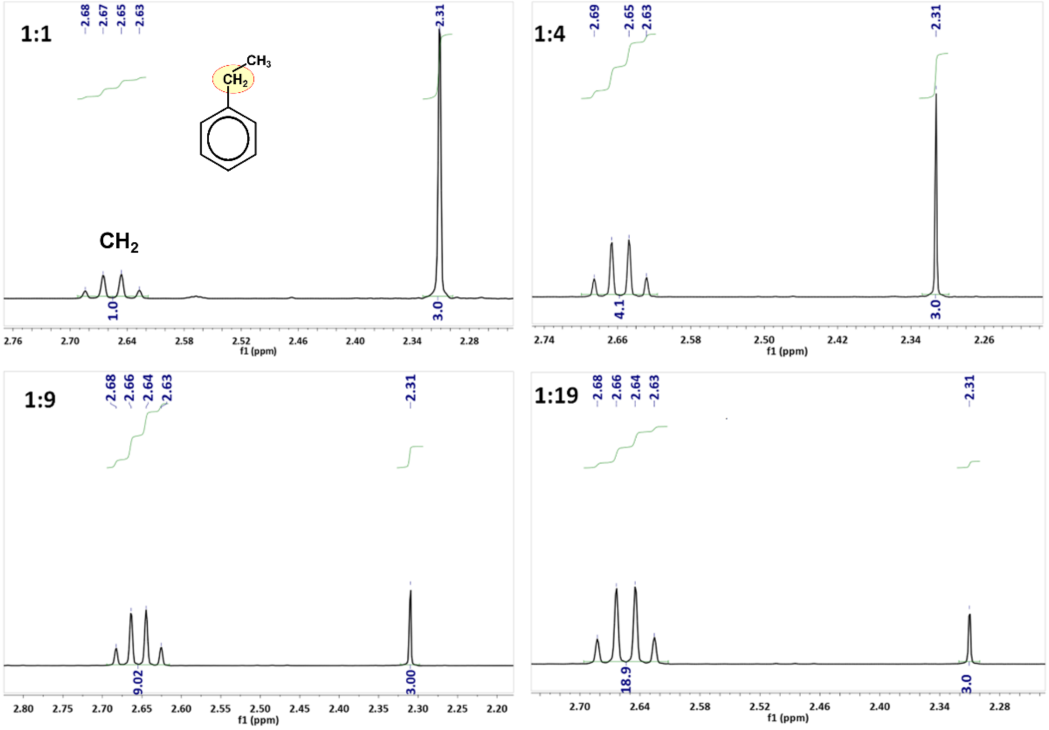

Figure S30. Methyl group region from the ^{1}H NMR spectrum of binary mixture feed of PX/EB with different ratios (1:1, 1:4, 1:9 and 1:19 mol:mol PX and EB).

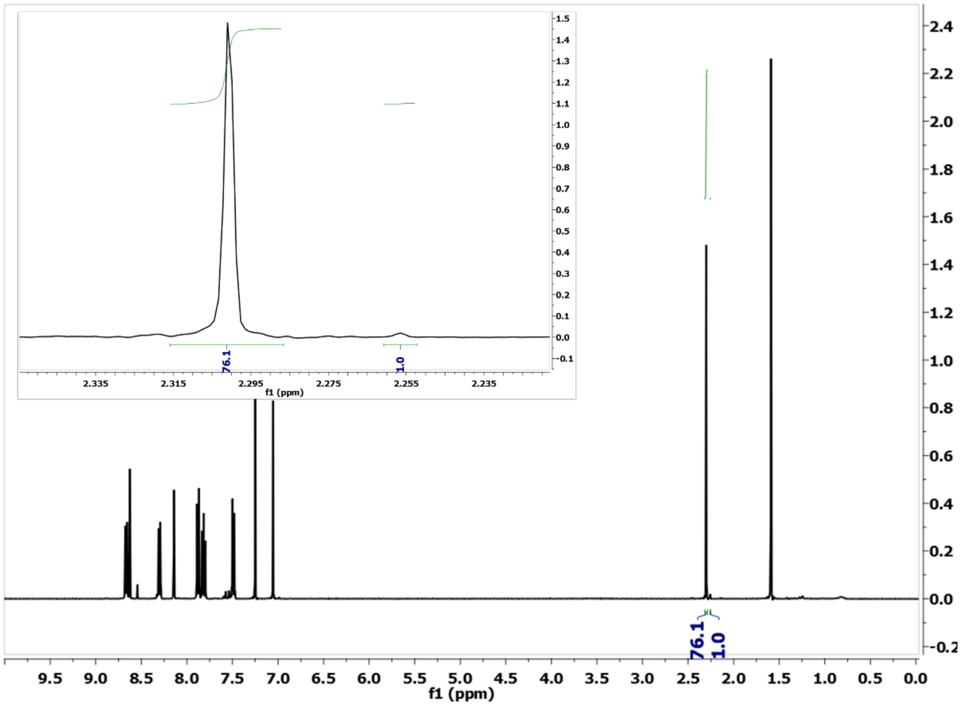

Figure S31. Relative uptake of xylene isomers by TPBD-I after being exposed to a binary mixture of PX:OX (1:1 mol:mol) for 4 days, determined using ^{1}H NMR

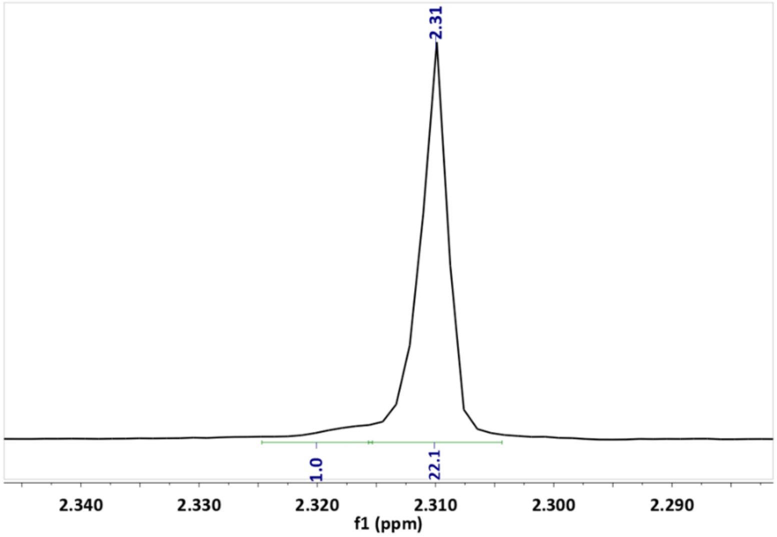

Figure S32. Relative uptake of xylene isomers by TPBD-I after being exposed to a binary mixture of PX: MX (1: 1 mol:mol) for 4 days, determined using ^{1}H NMR

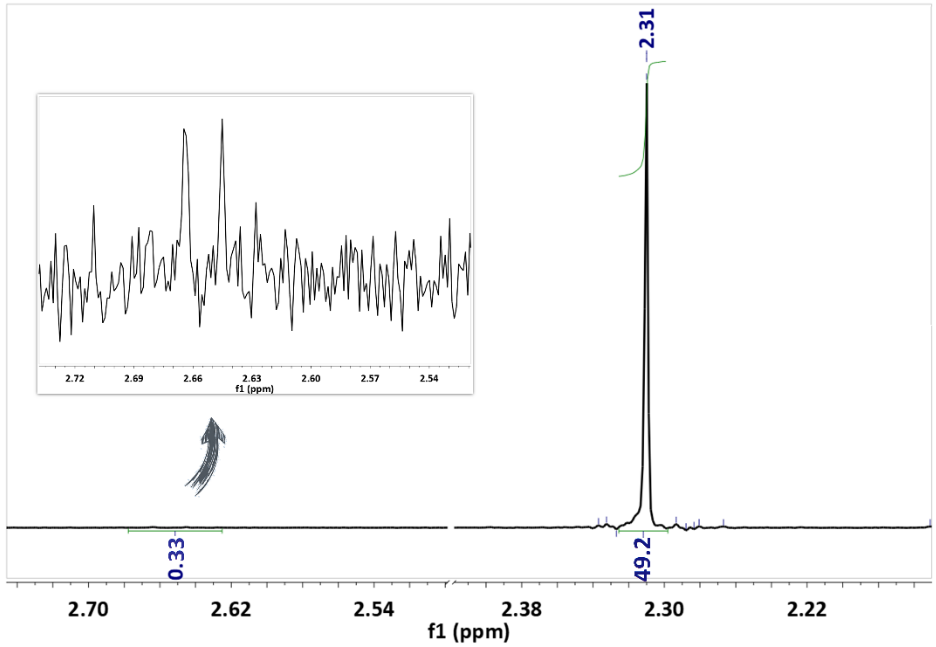

Figure S33. Relative uptake of xylene isomers by TPBD-I after being exposed to a binary mixture of PX: EB (1: 1 mol:mol) for 4 days, determined using ^{1}H NMR

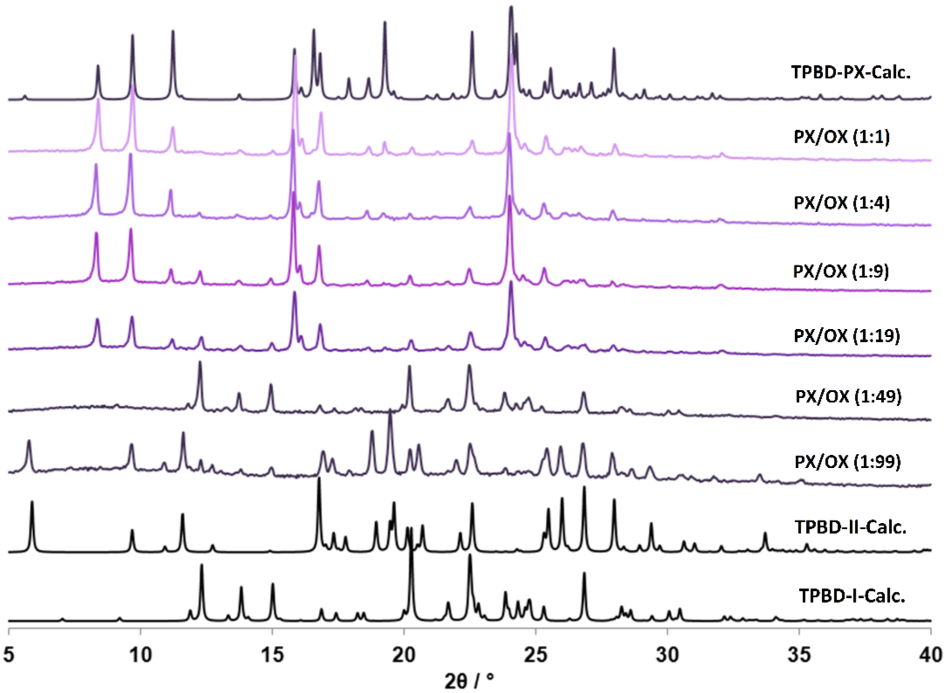

Figure S34. Experimental PXRD patterns for the liquid-exposed TPBD-I phase in binary mixture of PX/OX with difrent ratios (1:1, 1:4, 1:9, 1:19, 1:49 and 1:99 mol:mol PX and OX), comparing with the calculated PXRD patterns of TPBD-I, TPBD-II and TPBD-PX.

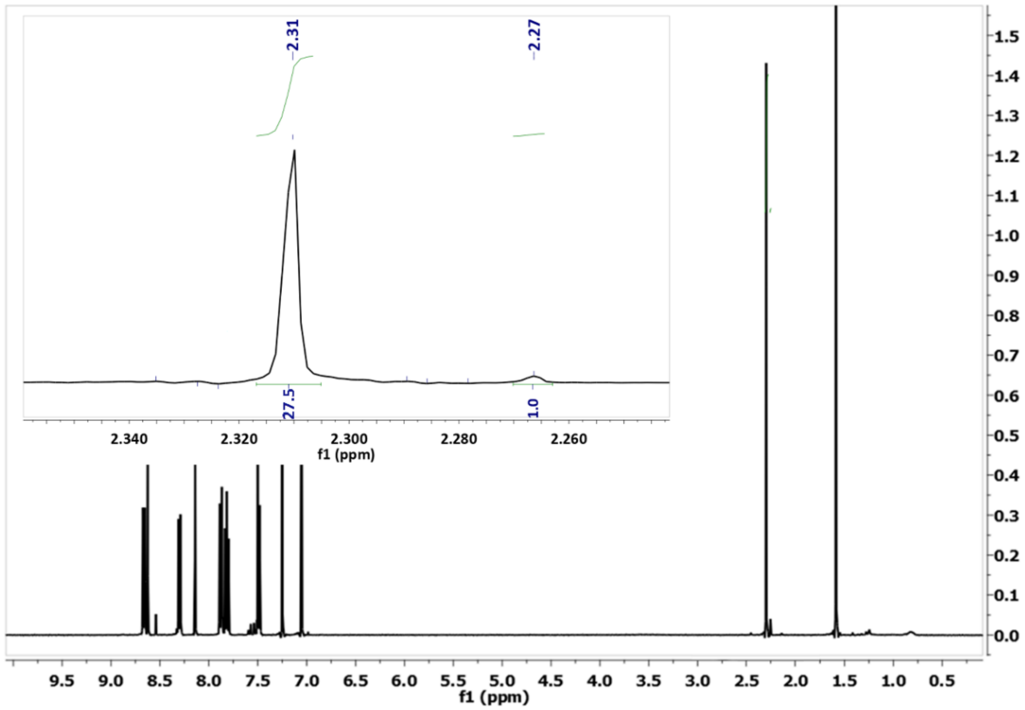

Figure S35. Relative uptake of xylene isomers by TPBD-I after being exposed to a binary mixture of PX: OX (1: 4 mol:mol) for 4 days, determined using ^{1}H NMR.

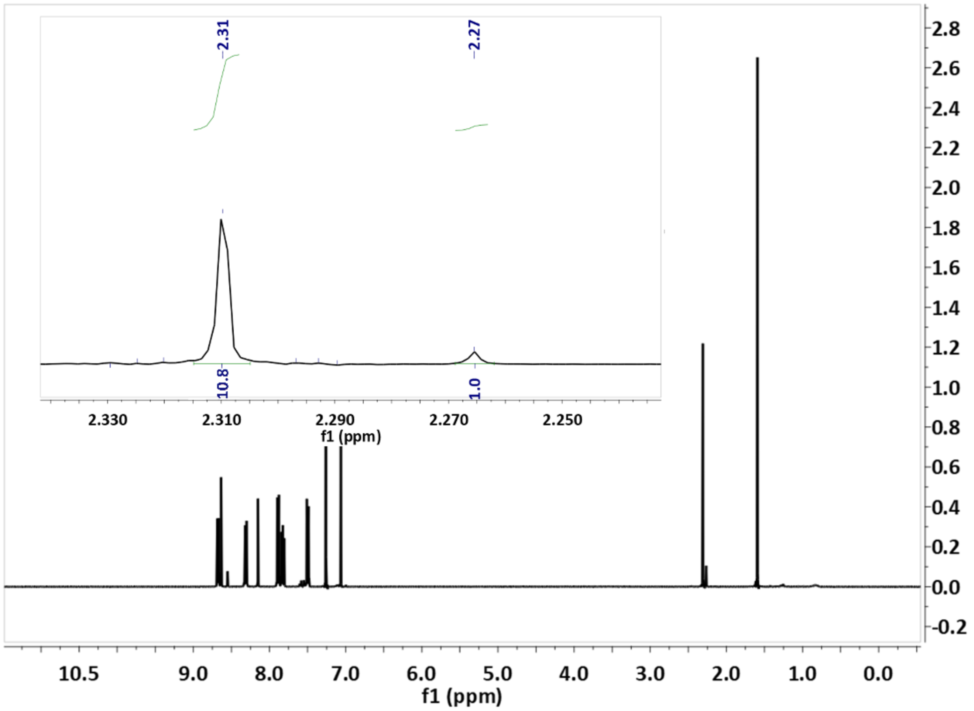

Figure S36. Relative uptake of xylene isomers by TPBD-I after being exposed to a binary mixture of PX: OX (1: 9 mol:mol) for 4 days, determined using ^{1}H NMR

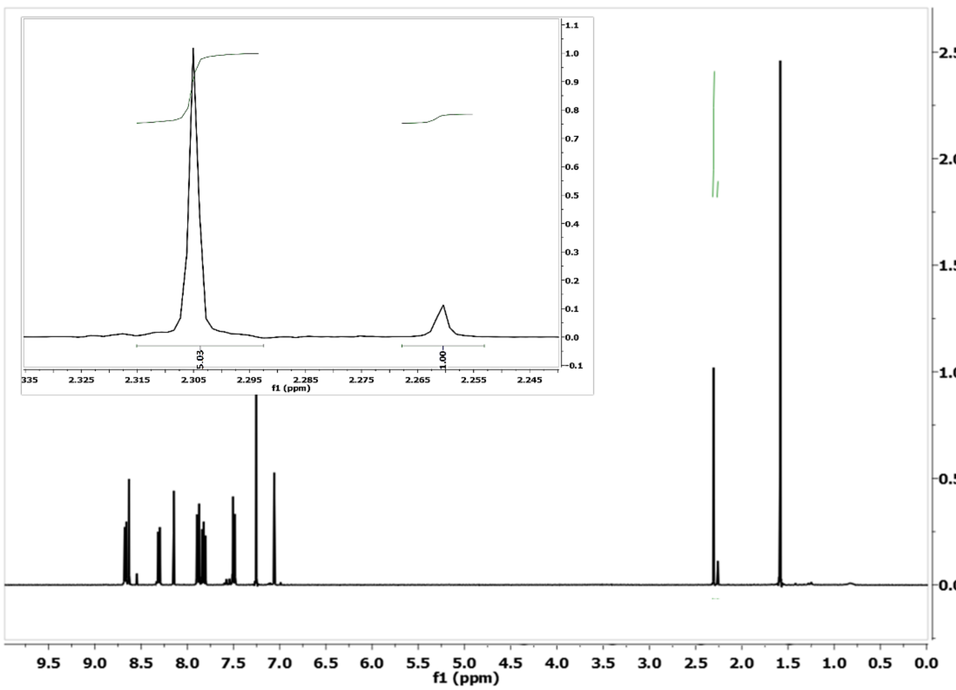

Figure S37. Relative uptake of xylene isomers by TPBD-I after being exposed to a binary mixture of PX: OX (1: 19 mol:mol) for 4 days, determined using ^{1}H NMR

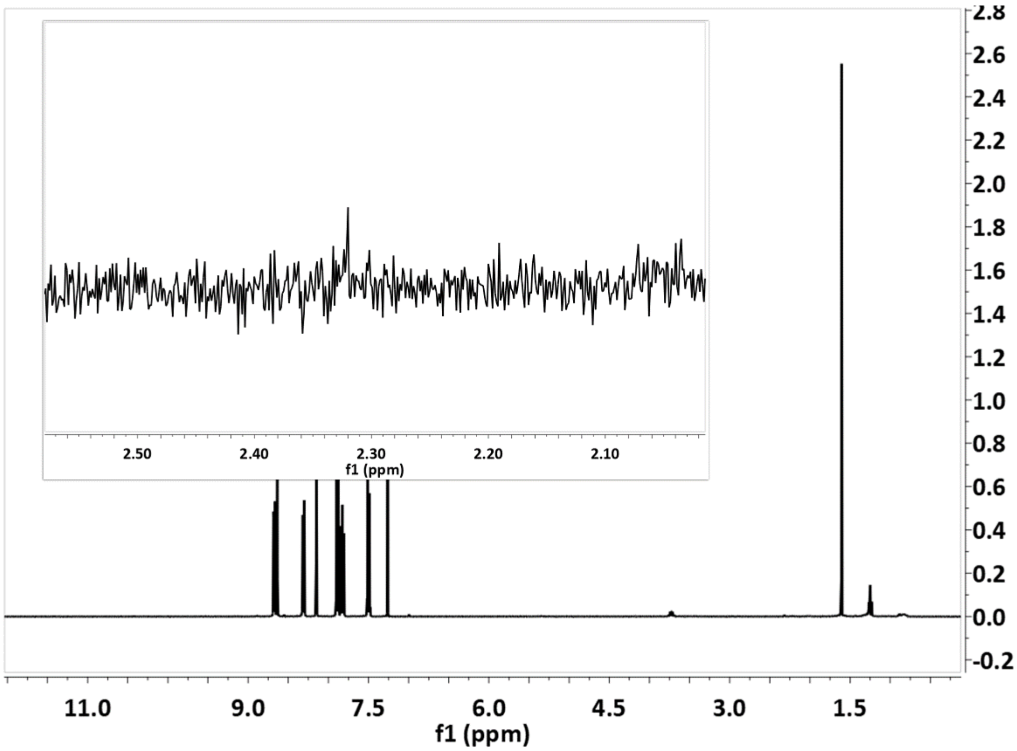

Figure S38. Relative uptake of xylene isomers by TPBD-I after being exposed to a binary mixture of PX: OX (1: 49 mol:mol) for 4 days,

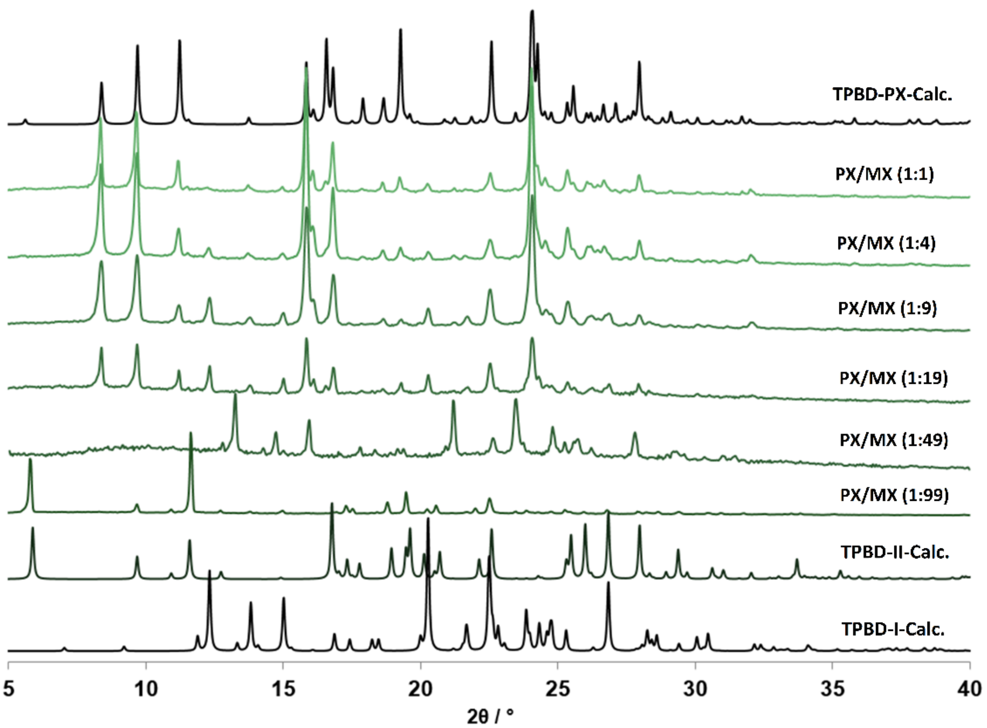

Figure S39. Experimental PXRD patterns for the liquid-exposed TPBD-I phase in binary mixture of PX/MX with difrent ratios (1:1, 1:4, 1:9, 1:19, 1:49 and 1:99 mol:mol PX and MX), comparing with the calculated PXRD patterns of TPBD-I, TPBD-II and TPBD-PX.

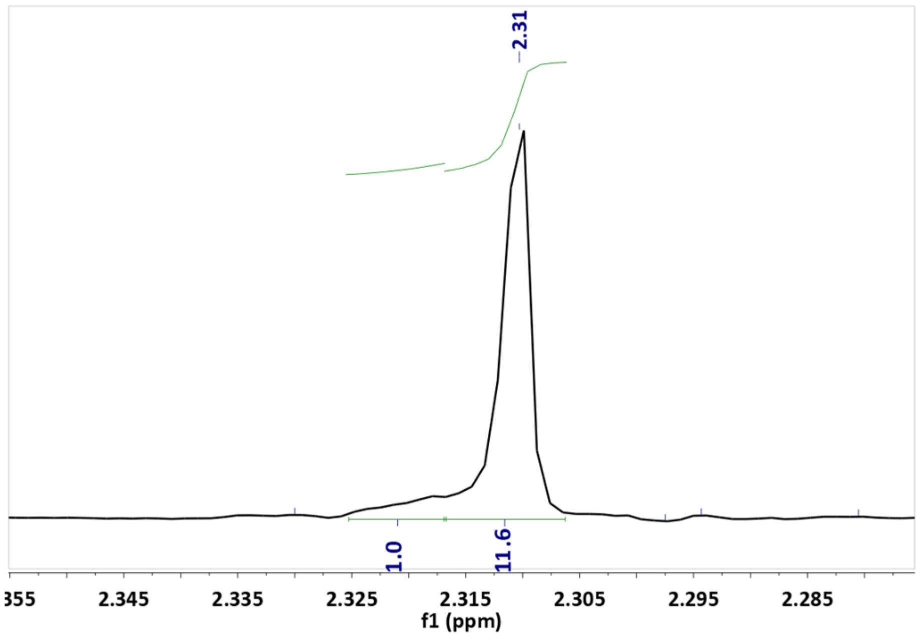

Figure S40. Relative uptake of xylene isomers by TPBD-I after being exposed to a binary mixture of PX: MX (1: 4 mol:mol) for 4 days, determined using ^{1}H NMR

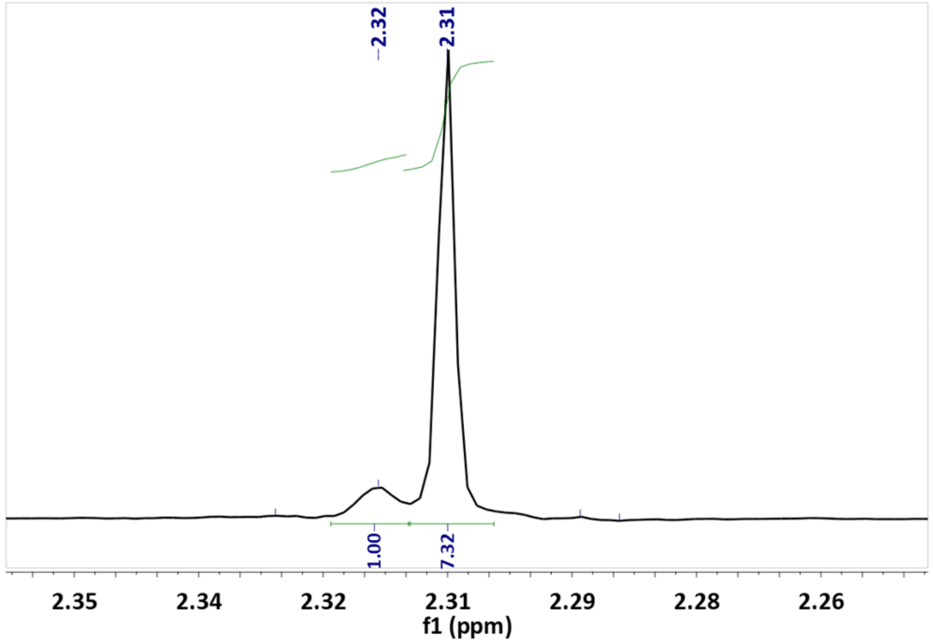

Figure S41. Relative uptake of xylene isomers by TPBD-I after being exposed to a binary mixture of PX: MX (1: 9 mol:mol) for 4 days, determined using ^{1}H NMR

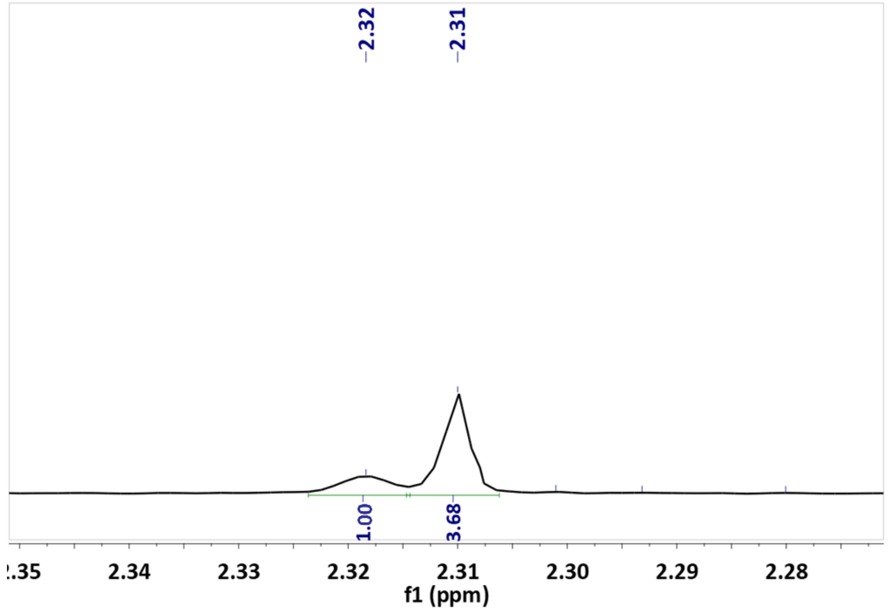

Figure S42. Relative uptake of xylene isomers by TPBD-I after being exposed to a binary mixture of PX: MX (1: 19 mol:mol) for 4 days, determined using ^{1}H NMR

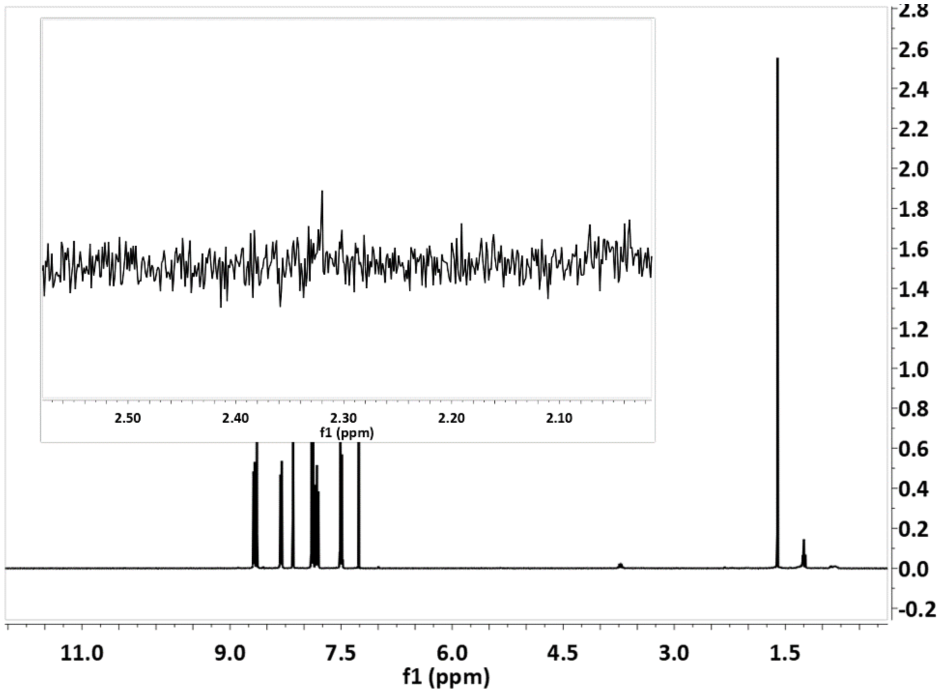

Figure S43. Relative uptake of xylene isomers by TPBD-I after being exposed to a binary mixture of PX: MX (1: 49 mol:mol) for 4 days, determined using ^{1}H NMR.

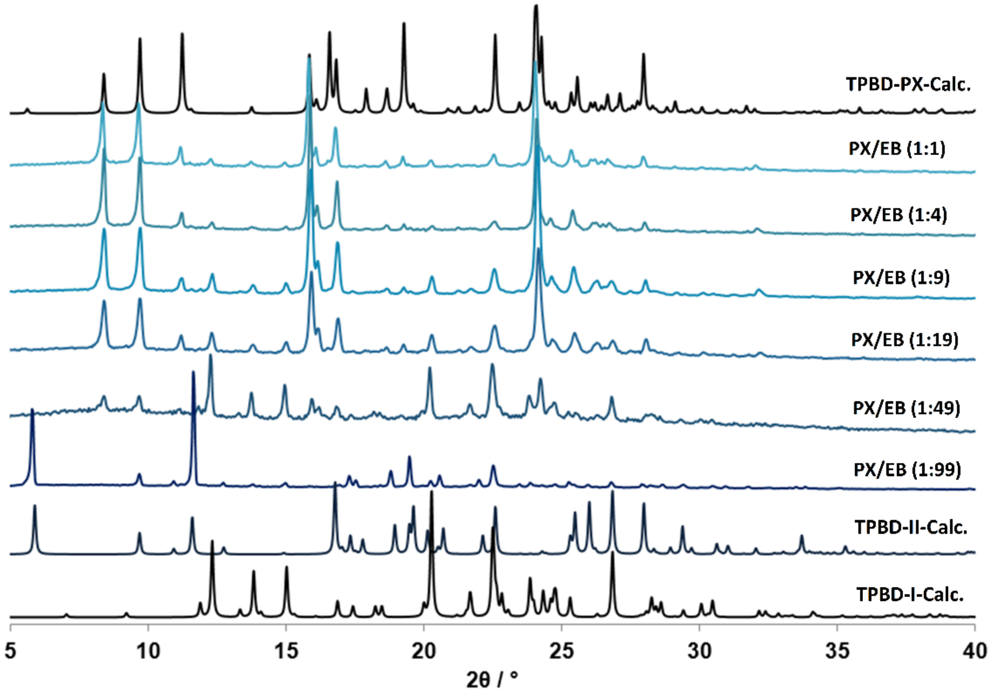

Figure S44. Experimental PXRD patterns for the liquid-exposed TPBD-I phase in binary mixture of PX/EB with difrent ratios (1:1, 1:4, 1:9, 1:19, 1:49 and 1:99 mol:mol PX and EB), comparing with the calculated PXRD patterns of TPBD-I, TPBD-II and TPBD-PX.

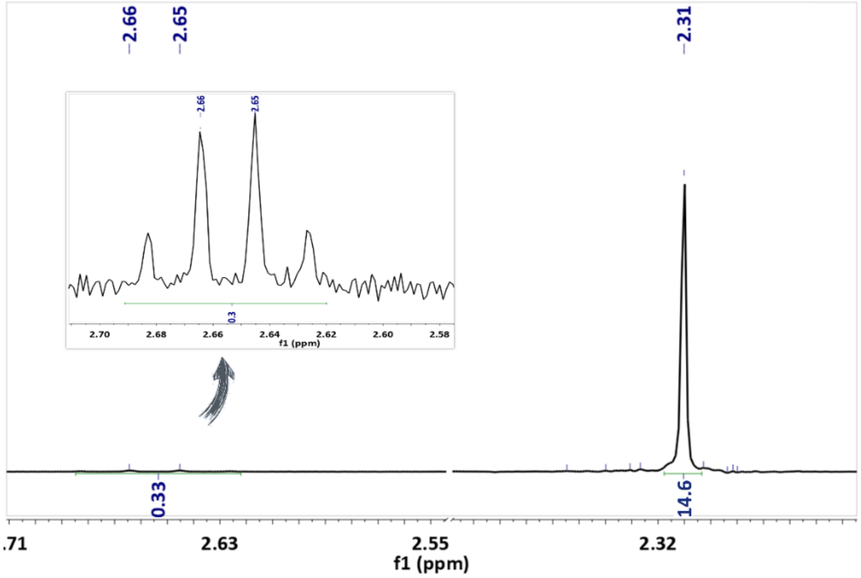

Figure S45. Relative uptake of xylene isomers by TPBD-I after being exposed to a binary mixture of PX: EB (1: 4 mol:mol) for 4 days, determined using ^{1}H NMR.

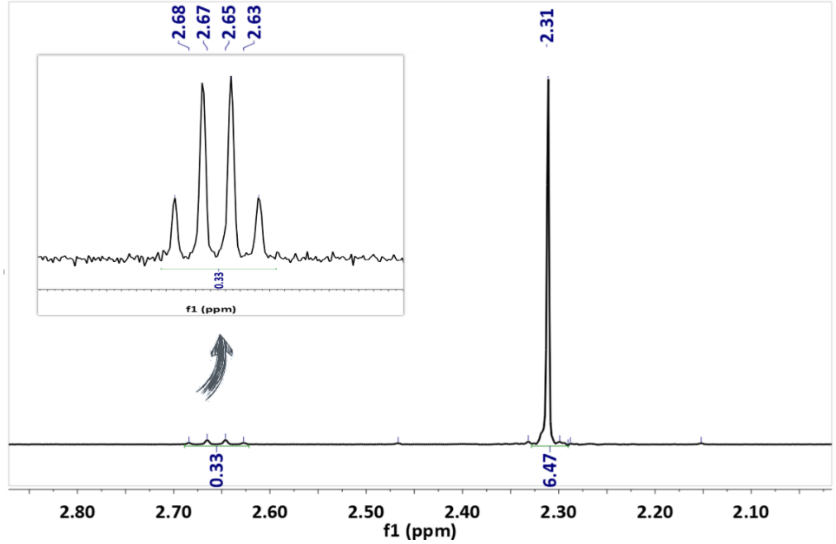

Figure S46. Relative uptake of xylene isomers by **TPBD-I** after being exposed to a binary mixture of PX: EB (1: 9 mol:mol) for 4 days, determined using ^{1}H NMR

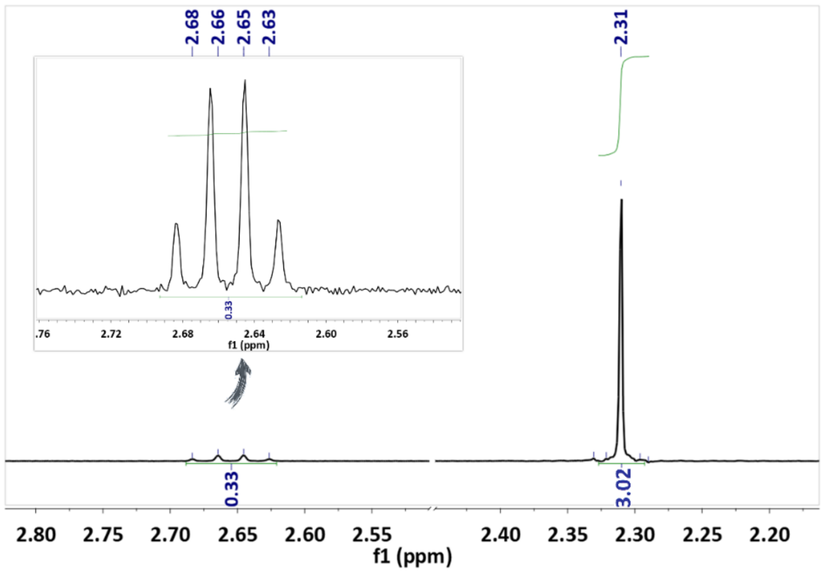

Figure S47. Relative uptake of xylene isomers by **TPBD-I** after being exposed to a binary mixture of PX: EB (1: 19 mol:mol) for 4 days, determined using ^{1}H NMR

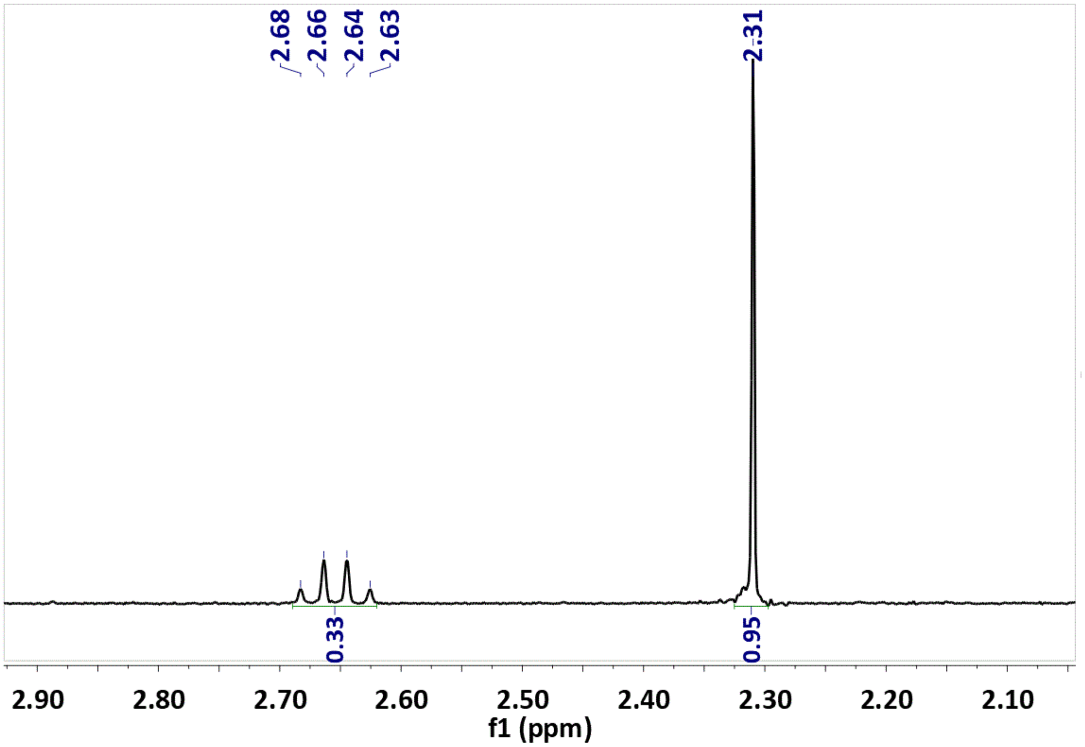

Figure S48. Relative uptake of xylene isomers by **TPBD-I** after being exposed to a binary mixture of PX: EB (1: 49 mol:mol) for 4 days, determined using ^{1}H NMR.

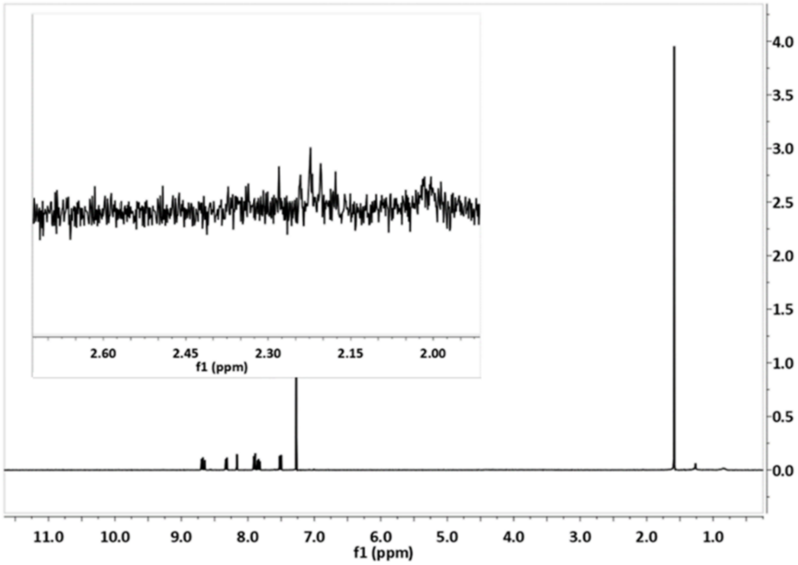

Figure S49. Relative uptake of xylene isomers by **TPBD-I** after being exposed to a binary mixture of PX: EB (1: 99 mol:mol) for 4 days, determined using ^{1}H NMR.

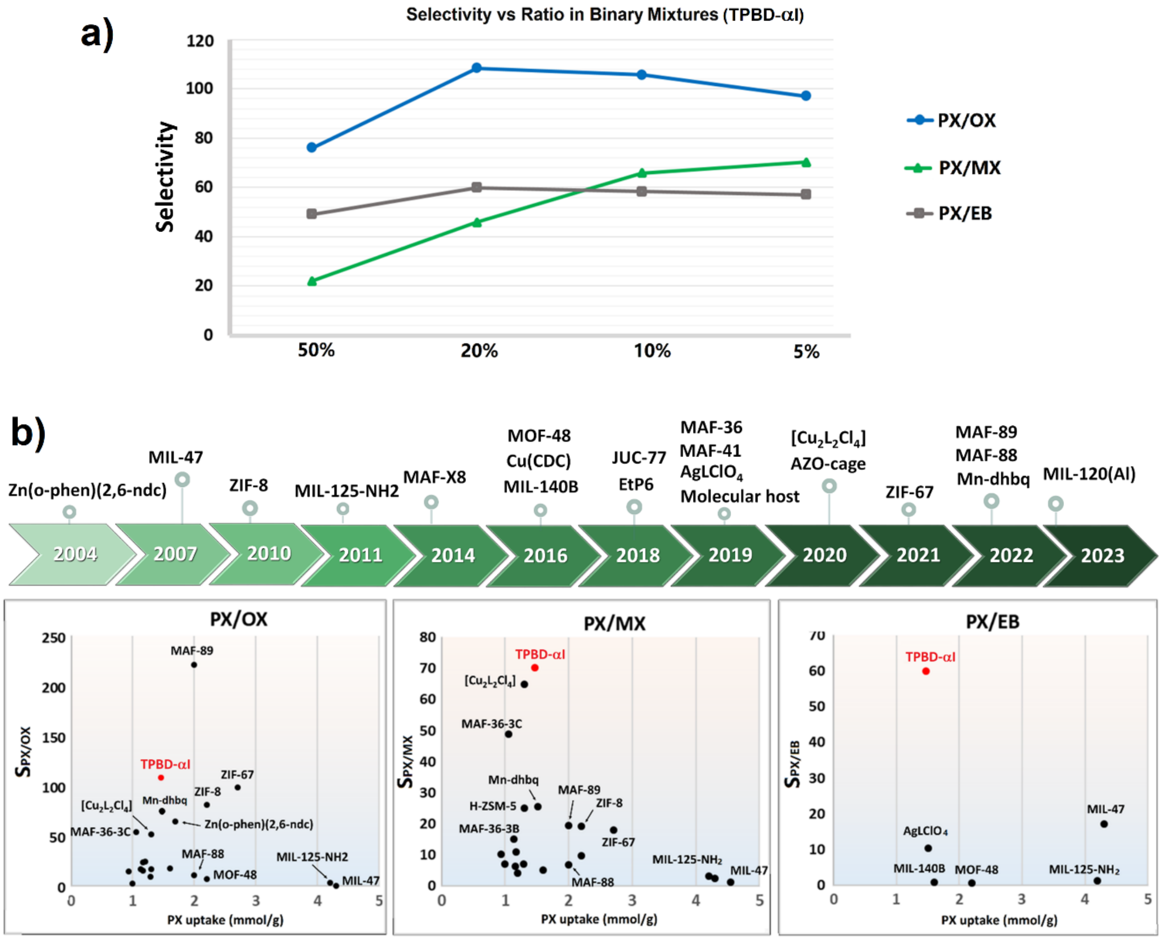

Figure S50. a) Performance of **TPBD-αI** in separation of PX in various molar ratios in binary mixtures and b) comparison of different absorbents for PX separation from binary mixtures (The experimental conditions may vary, like temperature, composition and characterization approach, which mentioned in Table S4). The highest and/or best selectivity values are given for each adsorbent.

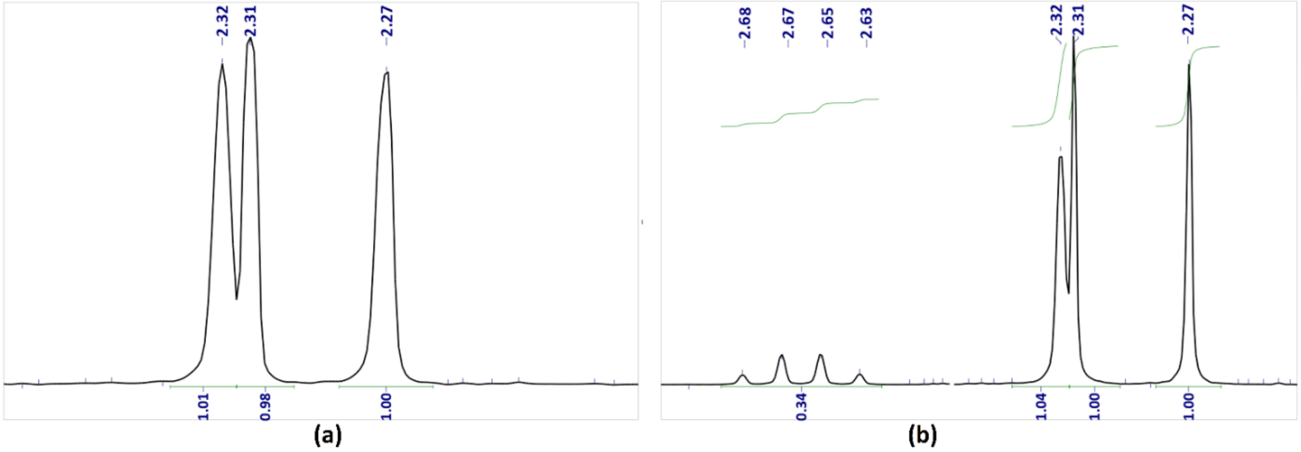

Figure S51: Methyl group region from the ^{1}H NMR spectrum of ternary (a) and quaternary (b) mixture of C8 aromatic isomers (PX: OX: MX: = 1.01: 0.98: 1.00 and PX: OX: MX: EB = 1.00: 1.00: 1.04: 1.02).

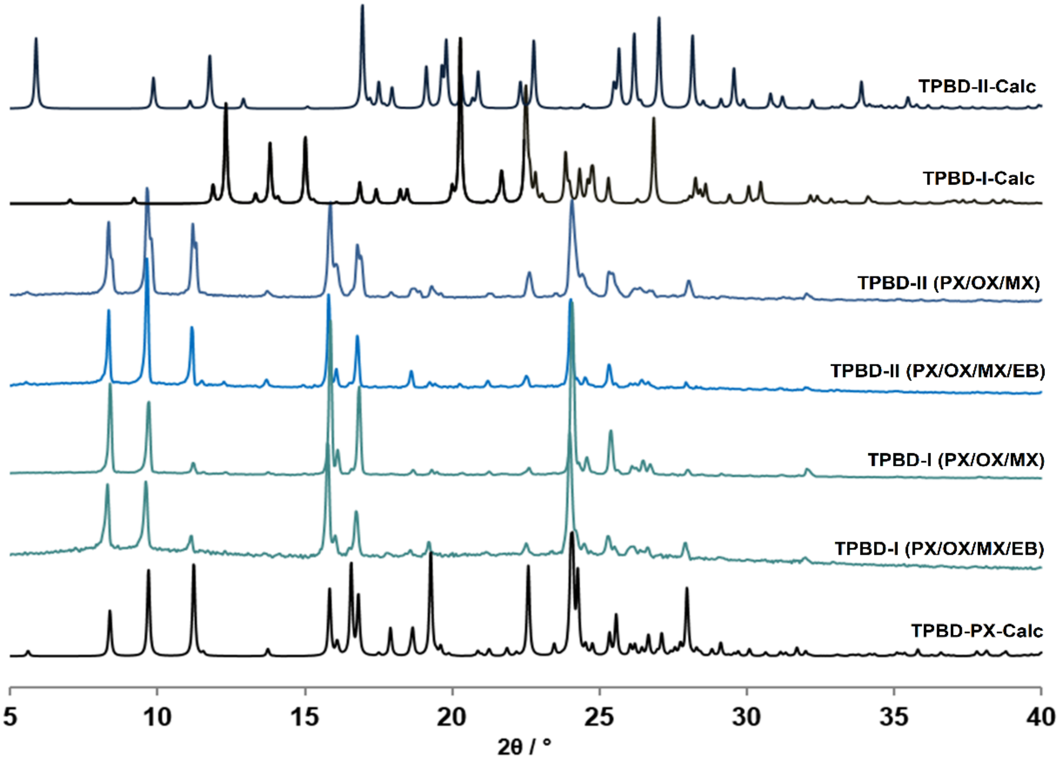

Figure S52. Experimental PXRD patterns for the liquid-exposed TPBD-I and TPBD-II phases: PX/MX/OX (ternary mixture of 1:1:1 mol:mol PX, MX and OX), and PX/MX/OX/EB (mixture of C8 aromatic isomers 1:1:1:1 mol:mol PX, MX, OX and EB), comparing with the calculated PXRD patterns of TPBD-I, TPBD-II and TPBD-PX

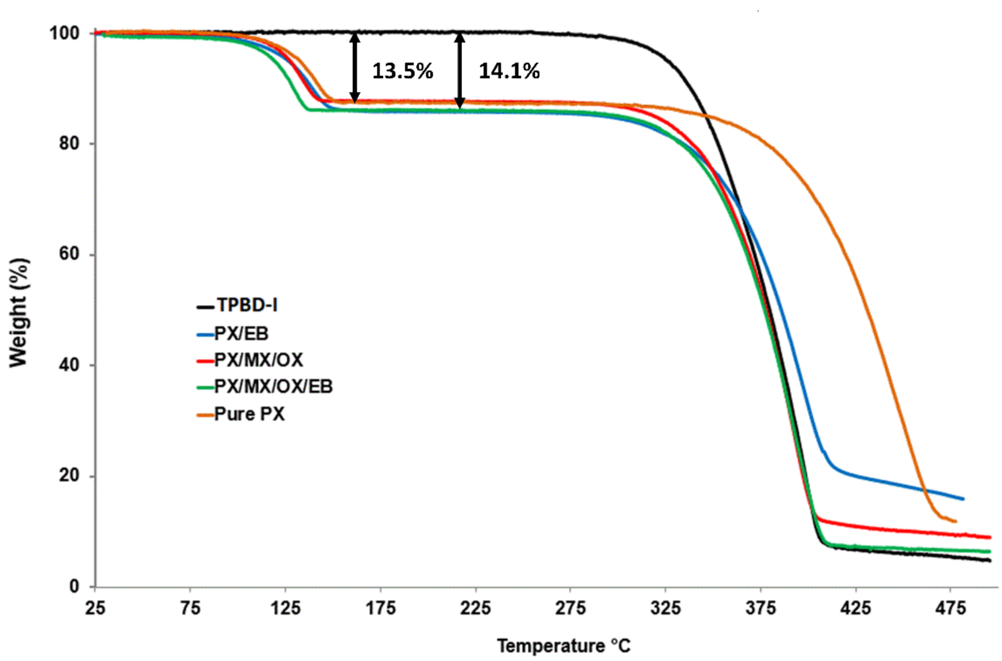

Figure S53. TGA plots from samples of TPBD-I immersed in pure PX, and equimolar binary, ternary and quaternary mixture of C8 aromatic isomers at 293K for 4 days.

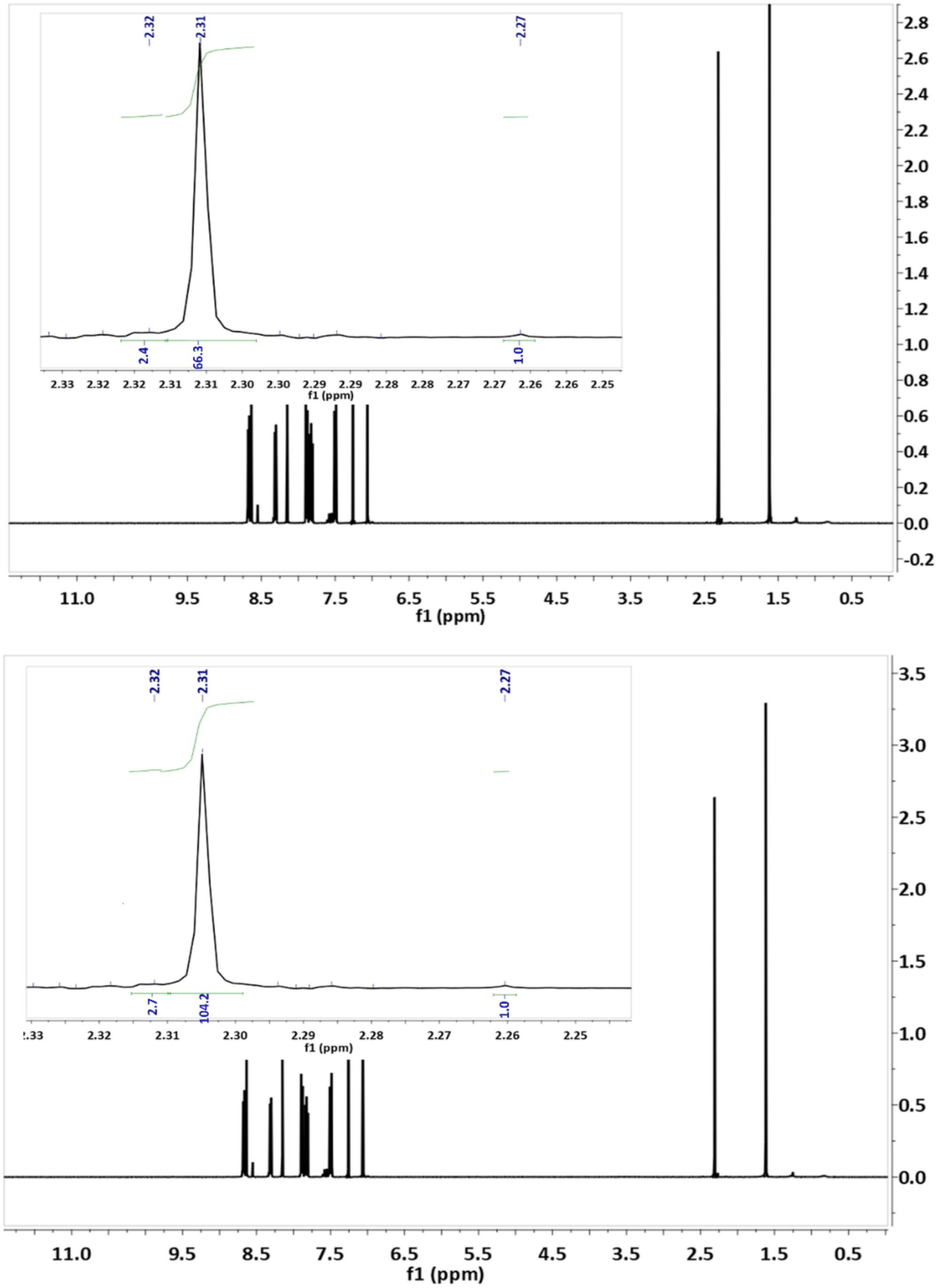

Figure S54. Relative uptake of xylene isomers by **TPBD-I** after being exposed to a mixture of three xylene isomers (PX: OX: MX= 1: 1: 1, mol:mol) for (Top) 1 hour and (Bottom) 4 days, determined using ^{1}H NMR. The sample was heated at 40°C for 30 minutes to eliminate any remaining xylene isomers on the surface.

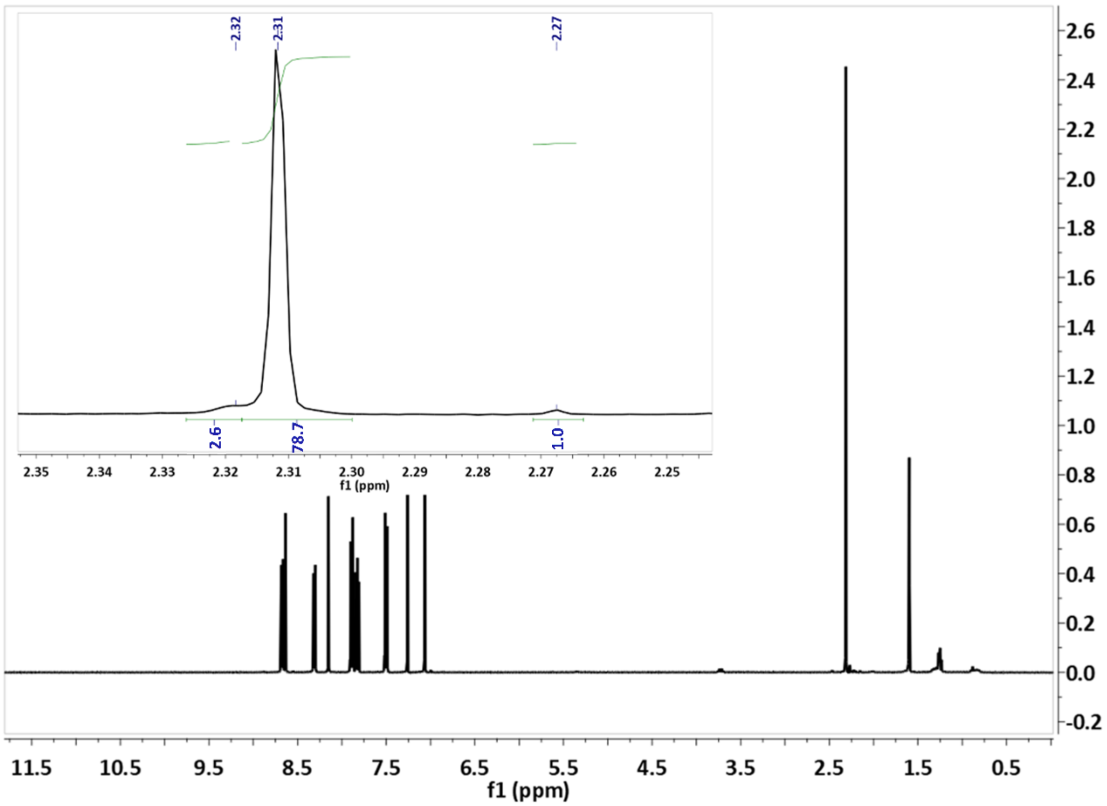

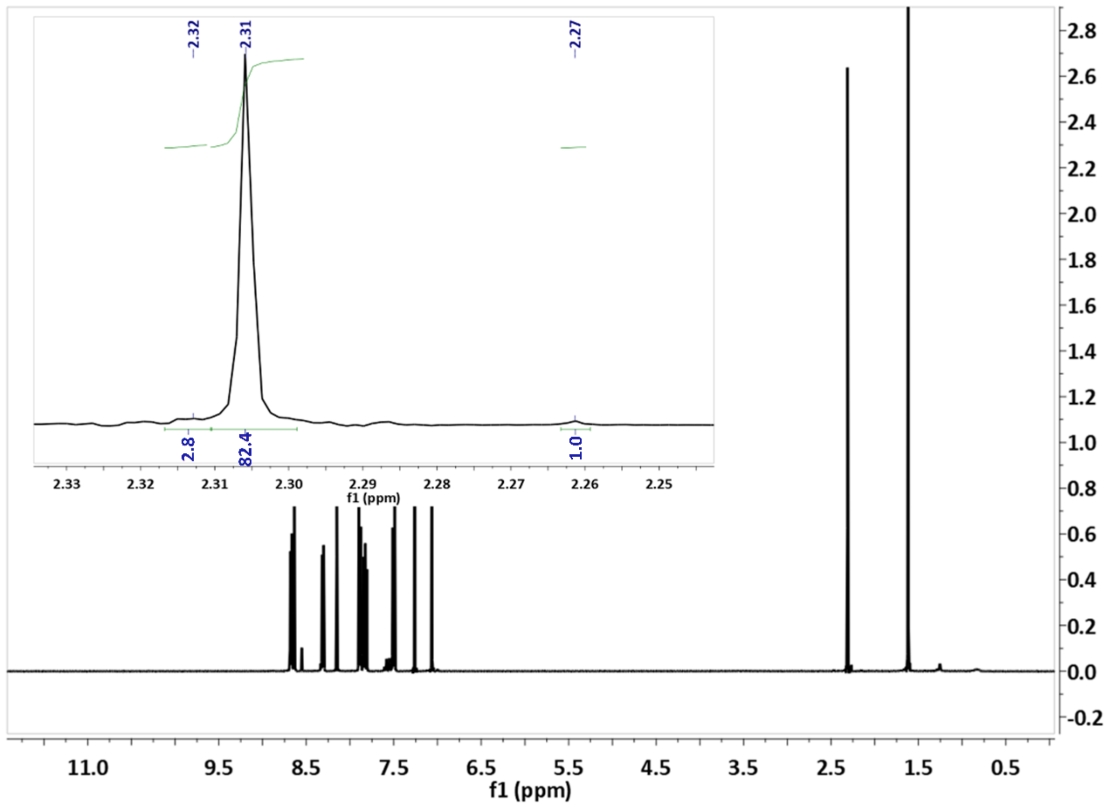

Figure S55. Relative uptake of xylene isomers by **TPBD-II** after being exposed to a mixture of three xylene isomers (PX: OX: MX= 1: 1: 1, mol:mol) for (Top) 1 hour and (Bottom) 4 days, determined using ^{1}H NMR. The sample was heated at 40°C for 30 minutes to eliminate any remaining xylene isomers on the surface.

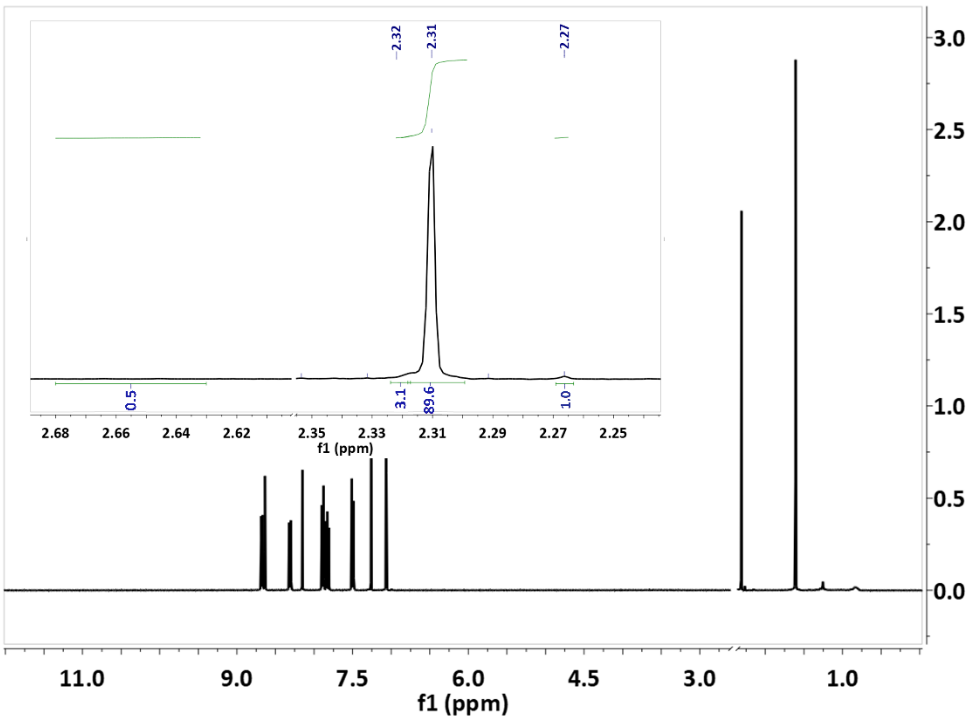

Figure S56. Relative uptake of xylene isomers by **TPBD-I** after being exposed to a mixture of C8 aromatic isomers (PX: OX: MX: EB = 1: 1: 1: 1, mol:mol) for 4 days, determined using ^{1}H NMR.

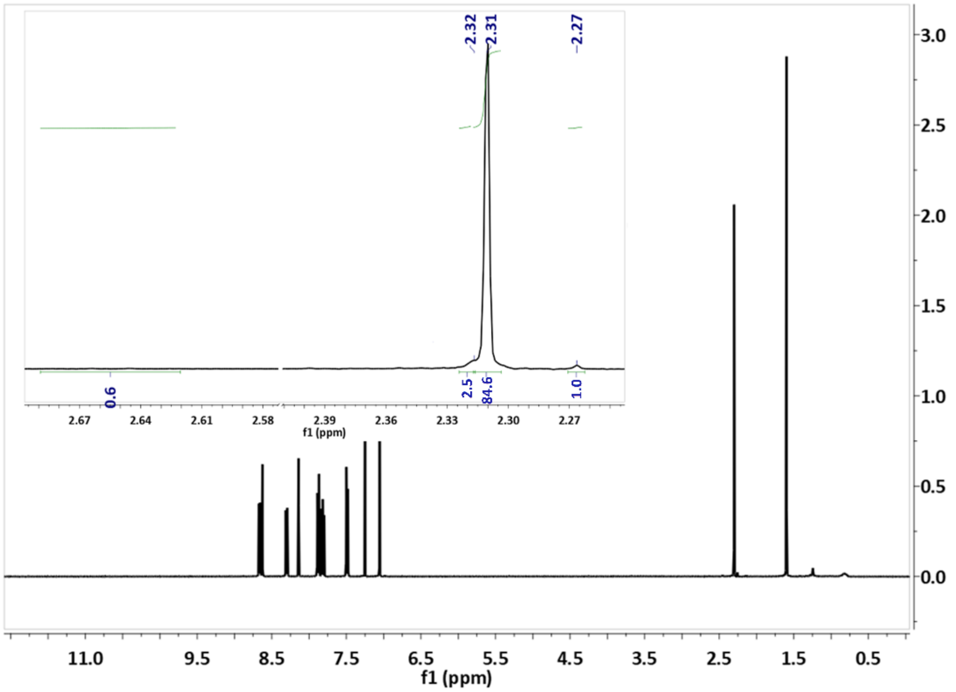

Figure S57. Relative uptake of xylene isomers by **TPBD-II** after being exposed to a mixture of C8 aromatic isomers (PX: OX: MX: EB = 1: 1: 1: 1, mol:mol) for 4 days, determined using ^{1}H NMR.

Table S9. The uptake capacity and PX/EB, MX/EB and OX/EB selectivity for various adsorbents in different mixture compositions and temperatures.

Compound	Category	Porosity/ Dimensionality	Capacity (mmol/g)	Temp (K)	$S_{PX/MX}$	$S_{PX/OX}$	$S_{PX/EB}$	$S_{PX/OX/MX}$	$S_{PX/OX/MX/EB}$	ref
TPBD-αI	Organic	NP/0D	1.51	298	22.1$^{L/N}$, 85.7$^{L/G}$ (1:1)<br>45.9$^{L/N}$ (1:4)<br>65.8$^{L/N}$ (1:9)<br>70.3$^{L/N}$ (1:19)	76.1$^{L/N}$, 85.7$^{L/G}$ (1:1)<br>108.4$^{L/N}$ (1:4)<br>105.6$^{L/N}$ (1:9)<br>97.1$^{L/N}$ (1:19)	49.2$^{L/N}$, 85.7$^{L/G}$ (1:1)<br>59.9$^{L/N}$ (1:4)<br>58.4$^{L/N}$ (1:9)<br>57.1$^{L/N}$ (1:19)	56.3$^{L/N}$, 60.4$^{L/G}$ (1:1:1)	48.5$^{L/N}$, 85.7$^{L/G}$ (1:1:1:1)	This work
$AgLClO_4$	Hybrid	NP/0D	NA	383	6.20^{L}(1:1)	24.00^{L}(1:1)	10.40^{L}(1:1)	NA	NA	14
Cu-metallocycle*	Hybrid	P/0D	1.32	295	65.7$^{L/G}$(1:1)	51.6$^{L/G}$(1:1)	NA	NA	NA	15
$[Cu_2Cl_4L_2]$ (MC)	Hybrid	P/0D	1.32	295	5.12$^{L/G}$(1:1)	11.40$^{L/G}$(1:1)	NA	7.1$^{L/G}$(1:1:1.2)	NA	16
AZO-cage	Organic	P/0D	1.17	298	7.30^{L}(1:1)	12.10^{L}(1:1)	NA	NA	NA	17
EtP6	Organic	P/0D	0.93	298	10.20^{V}(1:1)	14.28^{V}(1:1)	NA	NA	NA	18
Mn-dhbq*	Inorganic	P/1D	1.51	303<br>333<br>363<br>393	18.2$^{L/G}$(1:1)<br>18.0$^{L/G}$(1:1)<br>16.2$^{L/G}$(1:1)<br>25.5$^{L/G}$(1:1)	16.9$^{L/G}$(1:1)<br>74.5$^{L/G}$(1:1)<br>65.0$^{L/G}$(1:1)<br>66.8$^{L/G}$(1:1)	NA<br>NA<br>NA<br>NA	39.4$^{L/G}$(1:1:1)<br>35.7$^{L/G}$(1:1:1)<br>32.7$^{L/G}$(1:1:1)<br>48.3$^{L/G}$(1:1:1)	NA<br>NA<br>14.9$^{L/G}$(1:1:2:0.33)<br>25.1$^{L/G}$(1:1:2:0.33)	19
MAF-89*	Inorganic	P/3D	2.00	308	19.4$^{L/G}$(1:1)	221$^{L/G}$(1:1)	NA	46.4$^{L/G}$(1:1:1)	NA	20
MAF-88	Inorganic	P/3D	2.00	308	6.7$^{L/G}$(1:1)	10.7$^{L/G}$(1:1)	NA	1.6$^{L/G}$(1:1:1)	NA	20
ZIF-67	Inorganic	P/3D	2.7	298	18.1$^{L/G}$(1:1)	98.9$^{L/G}$(1:1)	NA	NA	NA	21
ZIF-8	Inorganic	P/3D	2.2	298	19.1$^{L/G}$(1:1)	81.2$^{L/G}$(1:1)	NA	NA	NA	22, 23
Zn(o-phen)(2,6-ndc)	Inorganic	P/3D	1.69		64.6^{L}(1:1)	NA	NA	NA	NA	24
Cu(CDC)	Inorganic	P/3D	1.29	298	7.0(1:1)	9.0(1:1)	NA	NA	NA	25
MIL-140B	Inorganic	P/3D	1.6	323	1.6(1:1)	1.8(1:1)	2.10(1:1)	NA	1.7$^{L/G}$(1:1:2:0.33)	26
MIL-125-NH2	Inorganic	P/3D	4.2	323	3.2(1:1)	3.5(1:1)	1.3(1:1)	NA	NA	27
MIL-47	Inorganic	P/3D	4.3	323	2.9$^{V/G}$ (1:1)	0.7$^{V/G}$ (1:1)	9.7$^{V/G}$ (1:1)	NA	0.6$^{V/G}$(1:1:2:0.33)	28
MOF-48	Inorganic	P/3D	2.2	323	9.8(1:1)	6.9(1:1)	0.7(1:1)	NA	1.7$^{L/G}$(1:1:2:0.33)	26
MAF-36-3C	Inorganic	P/3D	1.05	303	48.9(1:1)	53.9(1:1)	NA	51.3$^{L/G}$(1:1:1)	NA	29
MAF-36-3B	Inorganic	P/3D	1.13	303	15(1:1)	17(1:1)	NA	16$^{L/G}$(1:1:1)	NA	29
MAF-36-1B	Inorganic	P/3D	NA	303	14(1:1)	16(1:1)	NA	15$^{L/G}$(1:1:1)	NA	29
HIAM-201	Inorganic	P/3D	1.2	423	4.17^{V}(1:1)	24.25^{V}(1:1)	NA	NA	NA	30
KX	Inorganic	P/3D	0.95	423	5.36(1:1)	2.43(1:1)	3.22(1:1)	NA	NA	31
BaX	Inorganic	P/3D	0.97	423	7.19$^{V/G}$ (1:1)	2.82$^{V/G}$ (1:1)	3.75$^{V/G}$ (1:1)	NA	NA	32
X	Inorganic	P/3D	1.25	423	5.4(1:1)	4.8(1:1)	1.8(1:1)	NA	NA	33
FAU	Inorganic	P/3D	–	450	3.02(1:1)	2.84(1:1)	2.27(1:1)	NA	NA	34
H/ZSM-5	Inorganic	P/3D	1.34	443	25^{V}(1:1)	16.78^{V}(1:1)	6.76(1:1)	NA	NA	35

Note: NA = refers to not available; V = Vapor; L = Liquid.
NP = Nonporous, P = Porous
G = GC, N = ^{1}H NMR
For each material, only the best/highest values of selectivity were selected for comparison.
The results of vapor sorption were excluded from the comparison of selectivity in main text, and only liquid-phase separations were considered.

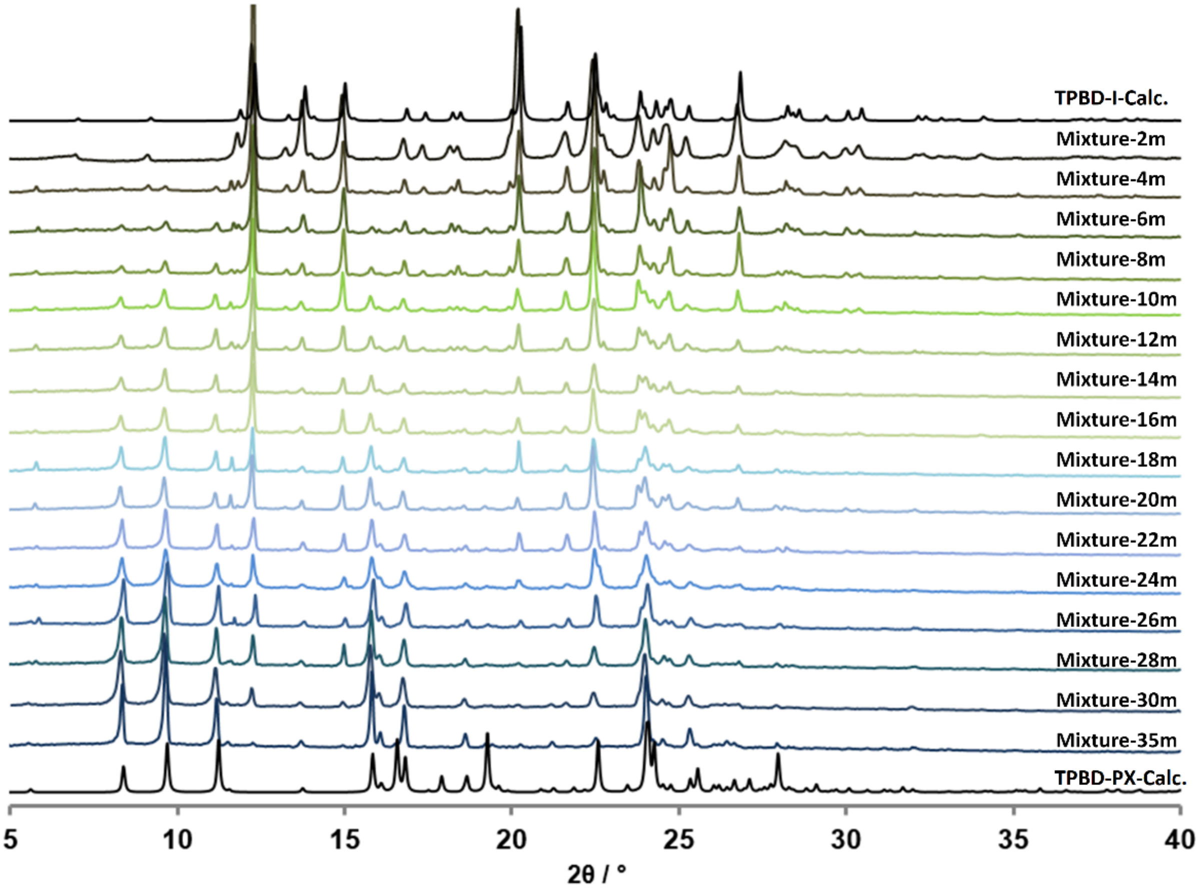

Figure S58. Experimental PXRD traces of **TPBD-I**, and the resulting inclusion compounds following a 20-minute slurry-assisted immersion of a mixture of C8 isomers (PX: OX: MX: EB = 1: 1: 1: 1, mol:mol) under ambient conditions.

Recyclability test

We performed 10 consecutive PX uptake/release cycles to study the recyclability of **TPBD-I**. Approximately 25 mg of **TPBD-I** were immersed in 100 μL of pure PX in a TGA pan for 2 hours at room temperature. The pan containing the sample was purged with N_2 flow (20 mL/min) at room temperature for 2 h to remove PX adhered on the surface, followed by heating from 25 to 150 °C for 20 minutes to fully uptake the PX. These uptake/release steps were repeated ten times in the same sample. Figures S59 and 5c show the relative working capacity of **TPBD-I** for PX sorption as determined by TGA. Figure S60 shows PXRD before and after cycling.

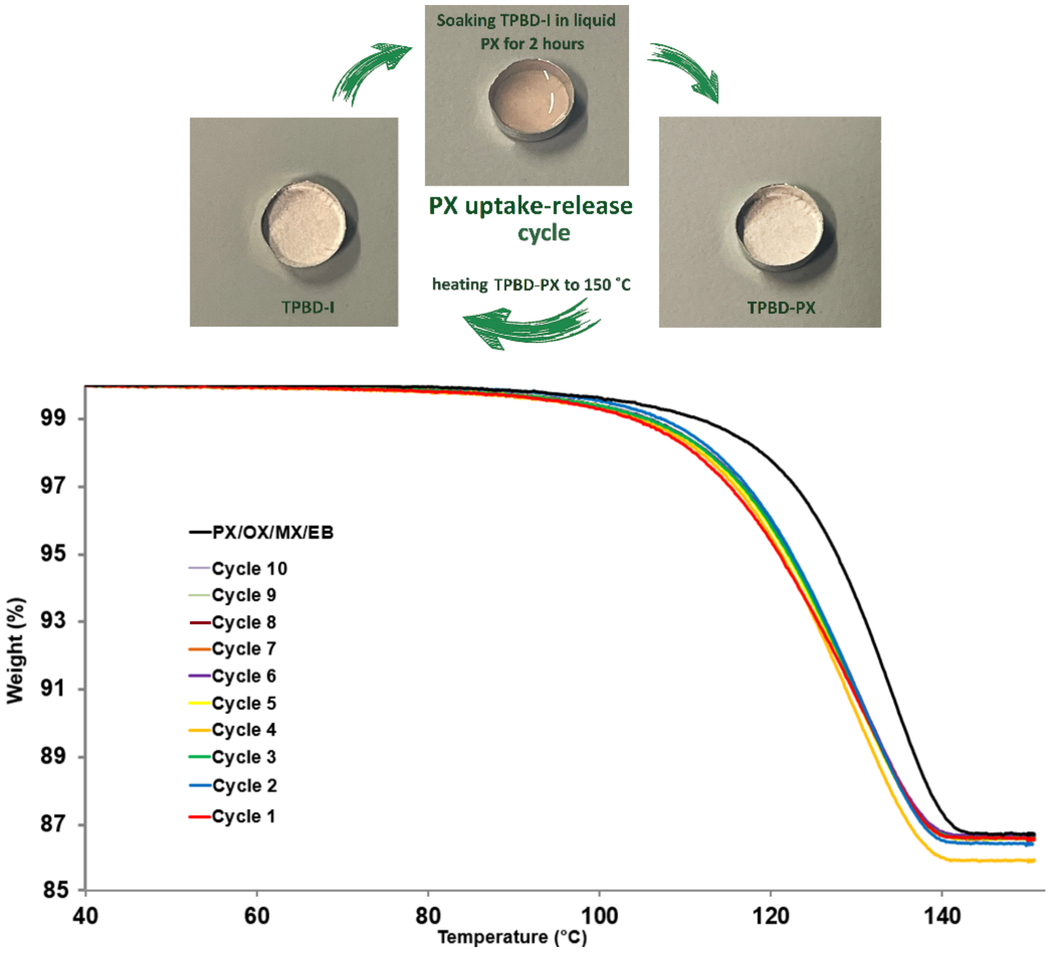

Figure S59. (Top) PX uptake/release recyclability test on TPBD-I. (Bottom) the uptake amount of PX for the 10 uptake/release cycles of TPBD-I.

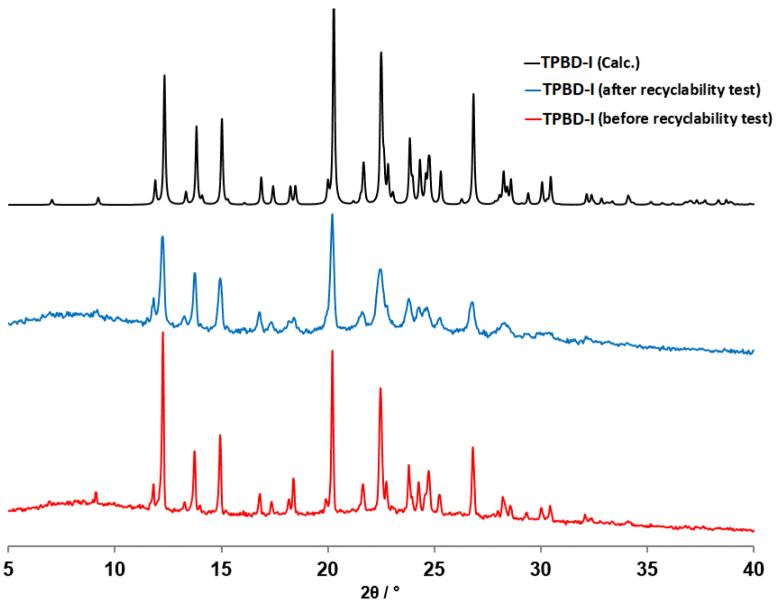

Figure S60. PXRD patterns of TPBD-I before and after recyclability test.

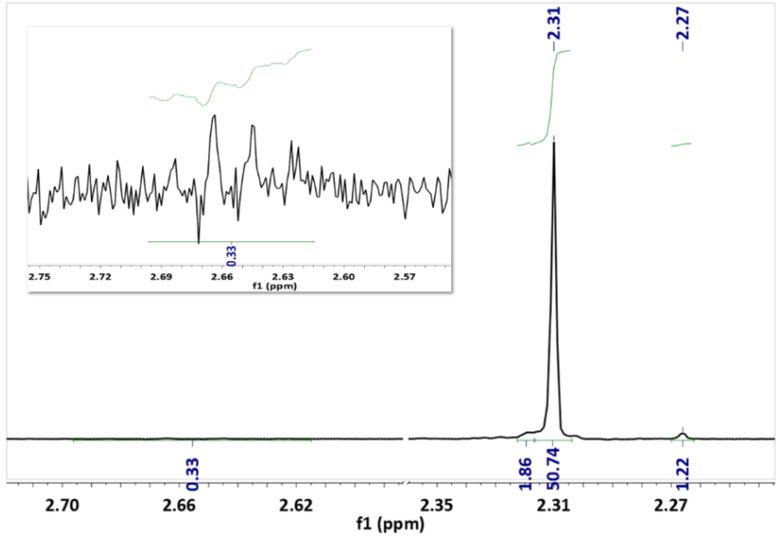

Figure S61. Relative uptake of xylene isomers by TPBD-I after recycling test by being exposed to a mixture of C8 isomers (PX: OX: MX: EB = 1: 1: 1: 1, mol:mol) for 2 hour, determined using ^{1}H NMR.

Dynamic separation of PX

We investigated TPBD's dynamic separation performance through fixed column breakthrough tests at room temperature (25±3 °C) for 2 hours, using an equimolar quaternary liquid mixture. A 500 mg apohost sample was placed in a syringe, mixed with approximately 975 µL of solution (considering three times the compound's 14 wt% working capacity), maintained at approximately 25°C for 2 hours, and filtered. The effectiveness of **TPBD-I** in isolating PX from other isomers was validated through (Figure S62b-S62c) ^{1}H NMR (solvent: DMSO), (Figure S62d) PXRD, and (Figure S62e) TGA analyses ($S_{PX/OX/MX/EB}$ = 27.398).

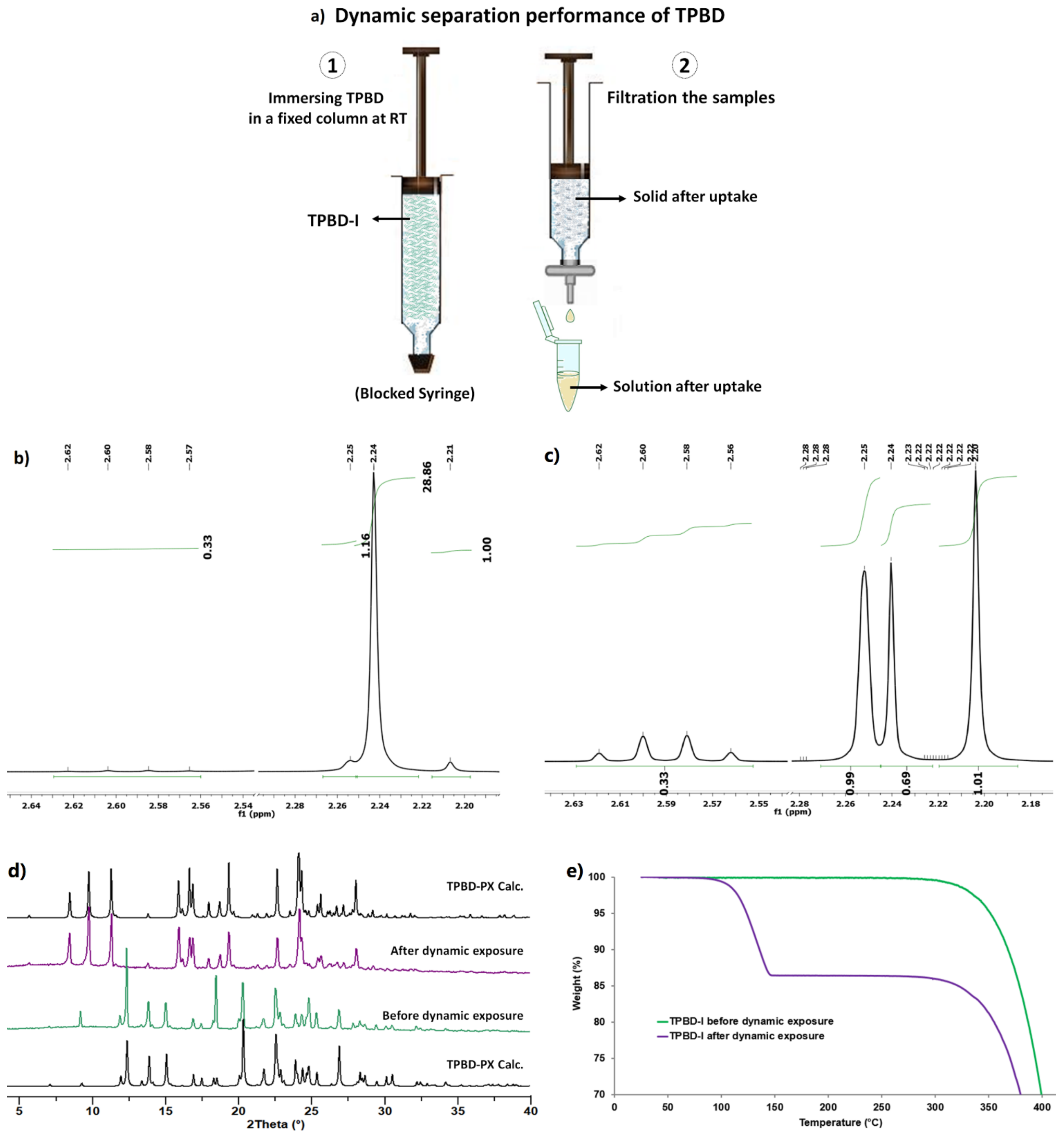

Figure S62. a) TPBD's dynamic separation experiment Relative uptake of xylene isomers in **TPBD-I** and in solution, after being exposed to a mixture of C8 isomers (PX: OX: MX: EB= 1: 1: 1:1, mol:mol) for 2 hour at 23±3 ˚C, determined using (b and c) ^{1}H NMR (solvent: DMSO), (d) PXRD, and (e) TGA.

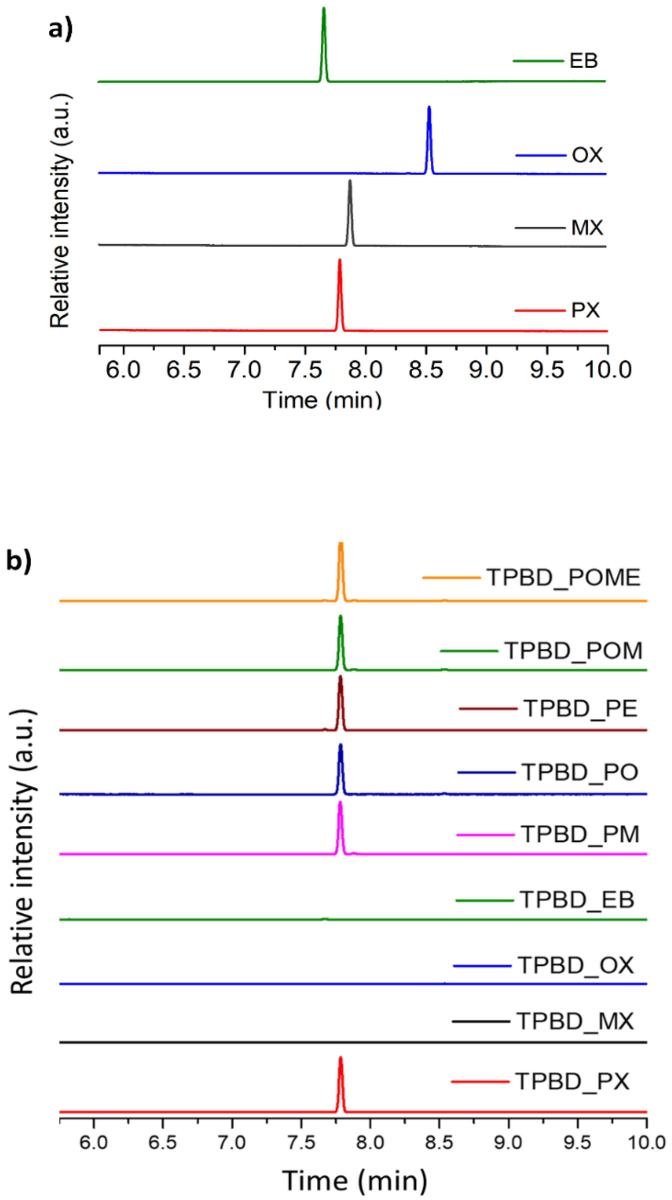

Figure S63. a) GC chromatograms of EB, PX, MX, and OX eluting from the column after 7.671, 7.786, 7.882, and 8.536 minutes, respectively. b) GC chromatograms for identification of the contents extracted from TPBD-I exposed to pure and equimolar mixtures of C8 isomers at 80 °C. Samples were dried on filter paper for 1-2 hours to remove liquid from the surface of the samples.

Table S10. Selectivity coefficients for liquid binary, ternary and quaternary mixtures of C8 isomers at 25 and 80 °C, measured by GC.

	**RT**	**80 °C**
**PX/MX**	50.6	32.6
**PX/OX**	54.1	36.0
**PX/EB**	47.3	25.4
**PX/OX/MX**	60.4	36.7
**PX/OX/MX/EB**	85.7	33.3

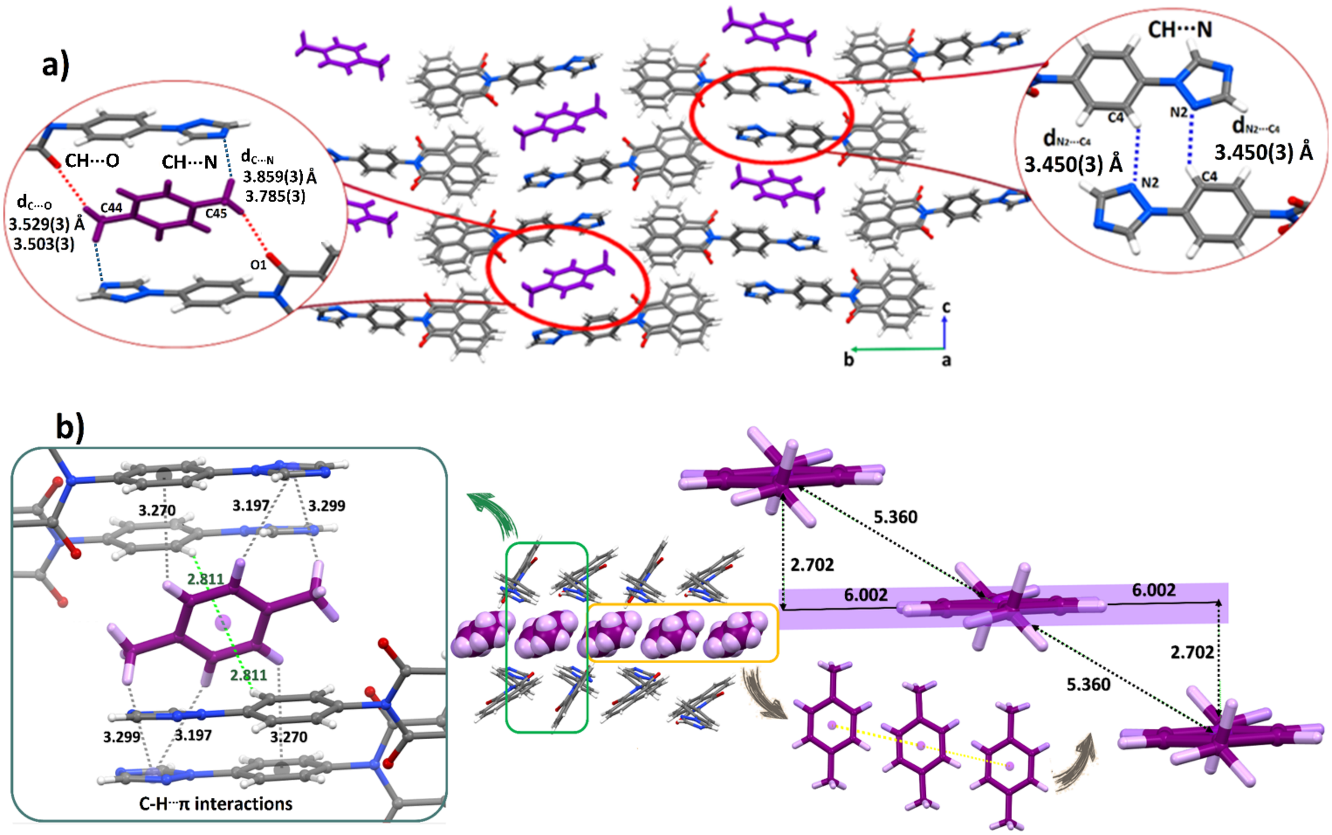

Figure S64. Perspective view showing the crystal packing of inclusion compound TPBD-PX projected along an axis (the PX guest molecule is shown in purple)

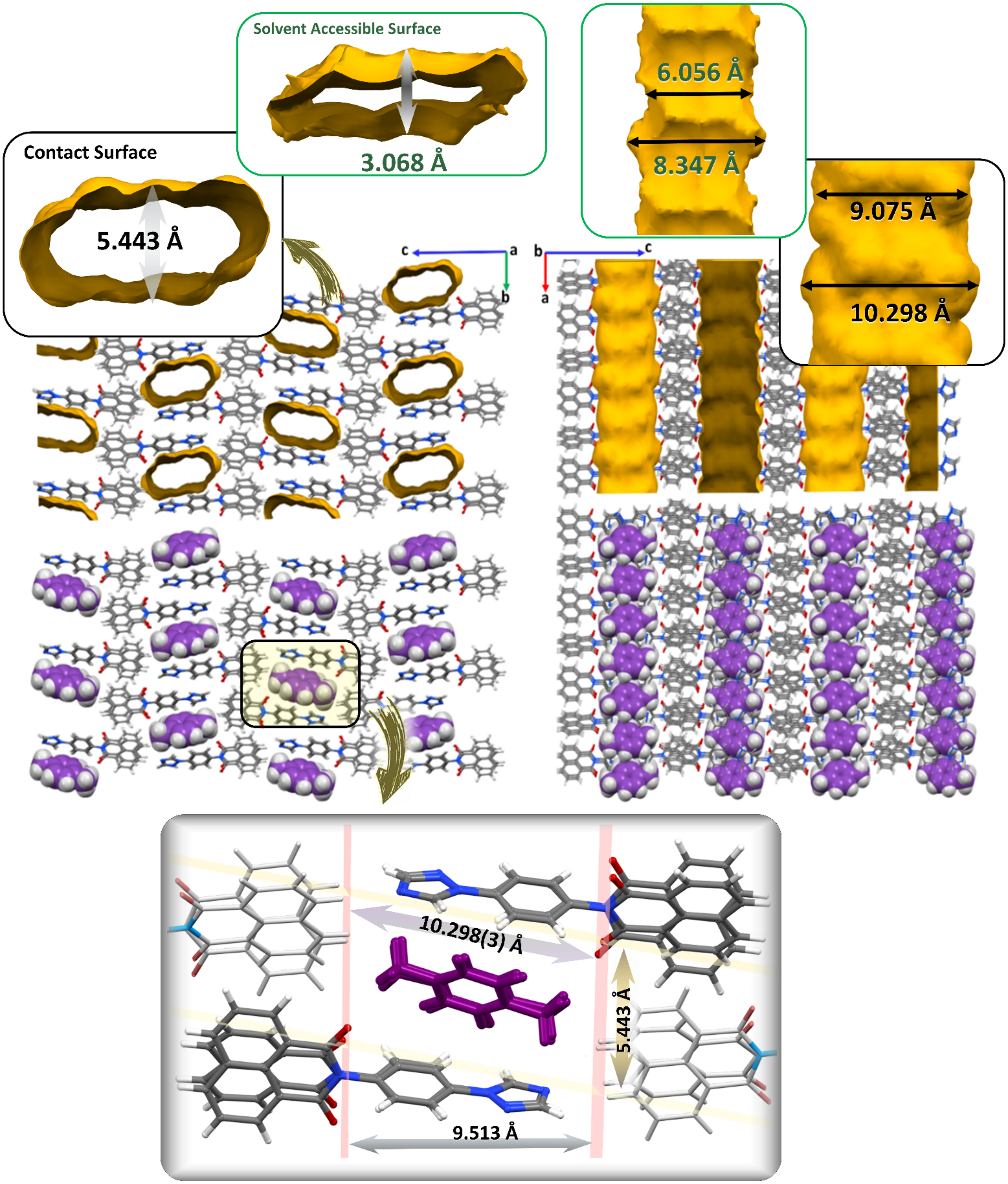

Figure S65. Detailed pore structures and guest-accessible volume per unit cell of TPBD-PX, was calculated in Mercury using a 1.2 Å probe radius and 0.2 Å grid spacing.

Modelling section

Table S11. Recrystallization energies, and Gibbs free energies starting from TPBD-I and gas phase C8 (p_{C8} = 1 bar) at 25, 50, 100 and 150 °C (in kJ/mol$_{C8}$).

	Structures optimized with experimental cell-parameters					Structures with optimized cell-parameters				
	ΔE	ΔG_{298}	ΔG_{323}	ΔG_{373}	ΔG_{423}	ΔE	ΔG_{298}	ΔG_{323}	ΔG_{373}	ΔG_{423}
$TPBD_8(PX)_4$	-77.4	-13.2	-8.6	0.4	9.3	-78.8	-14.8	-10.2	-1.1	7.8
$TPBD_8(MX)_4$	-60.9	-0.5	3.7	12.0	20.3	-67.8	-1.4	3.4	12.9	22.2
$TPBD_8(OX)_4$	-50.7	7.0	11.1	19.2	27.2	-61.9	-4.2	0.0	8.2	16.2
$TPBD_8(EB)_4$	-60.0	-4.7	-0.8	6.9	14.4	-65.6	-4.8	-0.4	8.2	16.7

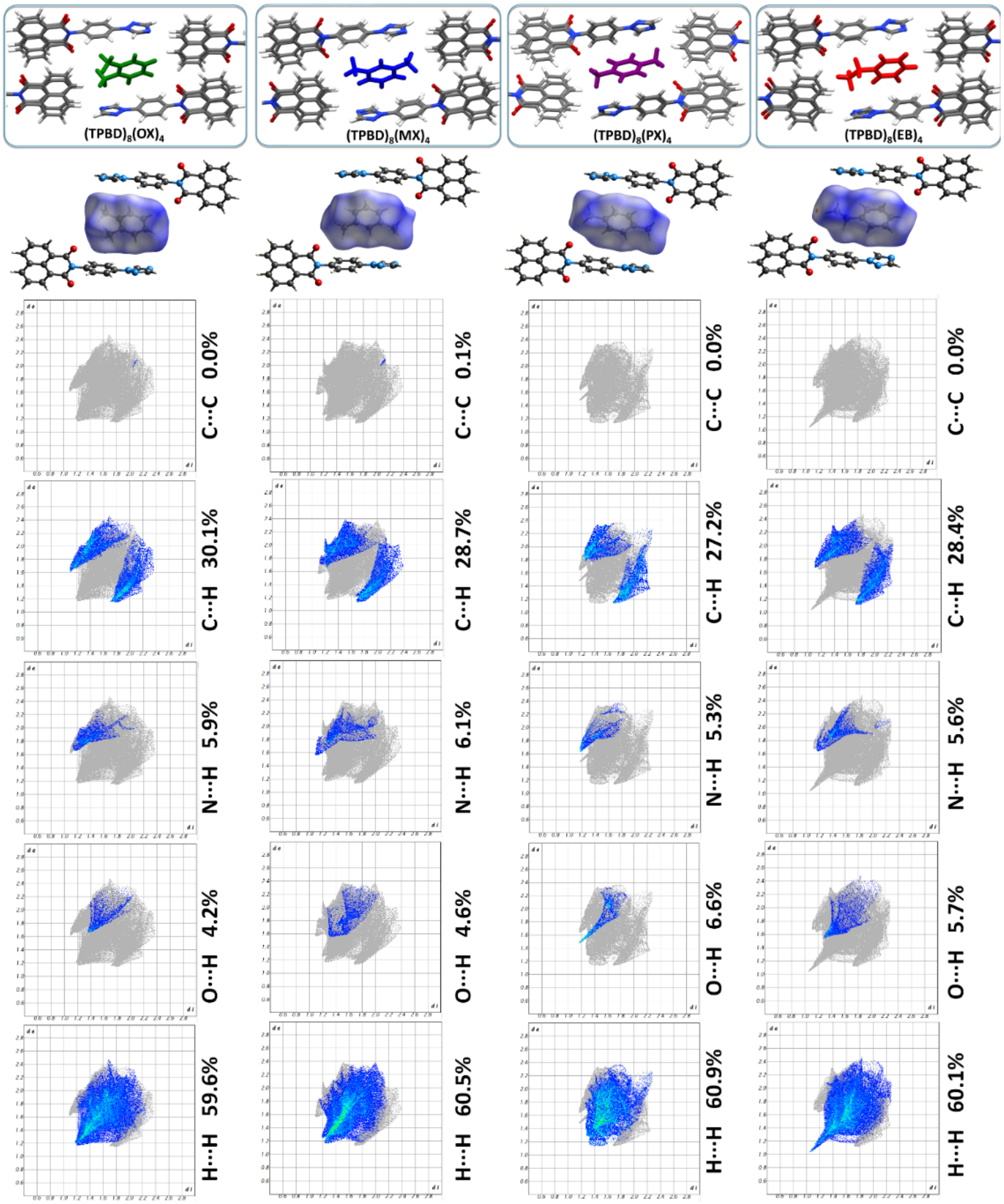

Figure S66. Relative contributions of various interactions to the Hirshfeld surface of the cell-optimized structures for TPBD hosting C8 guest molecules.

References

1. Bruker, A., SAINT V7. 36A. *Bruker AXS Inc., Madison, Wis., USA* **2012**.
2. Bruker, A., SAINT software reference manual. *Madison, WI* **1998,** *5465*.
3. Dolomanov, O. V.; Bourhis, L. J.; Gildea, R. J.; Howard, J. A. K.; Puschmann, H., OLEX2: a complete structure solution, refinement and analysis program. *Journal of Applied Crystallography* **2009,** *42* (2), 339-341.
4. Turner, M.; McKinnon, J.; Wolff, S.; Grimwood, D.; Spackman, P.; Jayatilaka, D.; Spackman, M., CrystalExplorer17. The University of Western Australia Australia: 2017.
5. Brouwers, J.; Brewster, M. E.; Augustijns, P., Supersaturating drug delivery systems: the answer to solubility-limited oral bioavailability? *Journal of pharmaceutical sciences* **2009,** *98* (8), 2549-2572.
6. Blöchl, P. E., Projector augmented-wave method. *Physical review B* **1994,** *50* (24), 17953.
7. Kresse, G.; Furthmüller, J., J Comp Mat Sci 6: 15–50 Blöchl PE (1994) Phys Rev B 50: 17953–17979 Kresse G. *Joubert D (1999) Phys Rev B* **1996,** *59*, 1758-1775.
8. Kresse, G.; Furthmüller, J., Phys. Rev. B: Condens. Matter Mater. Phys. **1996**.
9. Wellendorff, J.; Lundgaard, K. T.; Møgelhøj, A.; Petzold, V.; Landis, D. D.; Nørskov, J. K.; Bligaard, T.; Jacobsen, K. W., Density functionals for surface science: Exchange-correlation model development with Bayesian error estimation. *Physical Review B* **2012,** *85* (23), 235149.
10. Monkhorst, H. J.; Pack, J. D., Special points for Brillouin-zone integrations. *Physical review B* **1976,** *13* (12), 5188.
11. Ghysels, A.; Verstraelen, T.; Hemelsoet, K.; Waroquier, M.; Van Speybroeck, V., TAMkin: a versatile package for vibrational analysis and chemical kinetics. ACS Publications: 2010.
12. Perman, J. A.; Cairns, A. J.; Wojtas, Ł.; Eddaoudi, M.; Zaworotko, M. J., Cocrystal controlled solid-state synthesis of a rigid tetracarboxylate ligand that pillars both square grid and Kagomé lattice layers. *CrystEngComm* **2011,** *13* (9), 3130-3133.
13. Pivovar, A. M.; Holman, K. T.; Ward, M. D., Shape-Selective Separation of Molecular Isomers with Tunable Hydrogen-Bonded Host Frameworks. *Chemistry of Materials* **2001,** *13* (9), 3018-3031.
14. Sun, N.; Wang, S.-Q.; Zou, R.; Cui, W.-G.; Zhang, A.; Zhang, T.; Li, Q.; Zhuang, Z.-Z.; Zhang, Y.-H.; Xu, J.; Zaworotko, M. J.; Bu, X.-H., Benchmark selectivity p-xylene separation by a non-porous molecular solid through liquid or vapor extraction. *Chemical Science* **2019,** *10* (38), 8850-8854.
15. du Plessis, M.; Nikolayenko, V. I.; Barbour, L. J., Record-Setting Selectivity for p-Xylene by an Intrinsically Porous Zero-Dimensional Metallocycle. *Journal of the American Chemical Society* **2020,** *142* (10), 4529-4533.
16. Ye, J.; du Plessis, M.; Loots, L.; van Wyk, L. M.; Barbour, L. J., Solid–Liquid Separation of Xylene Isomers Using a Cu-Based Metallocycle. *Crystal Growth & Design* **2022,** *22* (4), 2654-2661.
17. Moosa, B.; Alimi, L. O.; Shkurenko, A.; Fakim, A.; Bhatt, P. M.; Zhang, G.; Eddaoudi, M.; Khashab, N. M., A Polymorphic Azobenzene Cage for Energy-Efficient and Highly Selective p-Xylene Separation. *Angewandte Chemie International Edition* **2020,** *59* (48), 21367-21371.
18. Jie, K.; Liu, M.; Zhou, Y.; Little, M. A.; Pulido, A.; Chong, S. Y.; Stephenson, A.; Hughes, A. R.; Sakakibara, F.; Ogoshi, T.; Blanc, F.; Day, G. M.; Huang, F.; Cooper, A. I., Near-Ideal Xylene Selectivity in Adaptive Molecular Pillar[n]arene Crystals. *Journal of the American Chemical Society* **2018,** *140* (22), 6921-6930.
19. Li, L.; Guo, L.; Olson, D. H.; Xian, S.; Zhang, Z.; Yang, Q.; Wu, K.; Yang, Y.; Bao, Z.; Ren, Q.; Li, J., Discrimination of xylene isomers in a stacked coordination polymer. *Science* **2022,** *377* (6603), 335-339.
20. Ye, Z.-M.; Zhang, X.-F.; Liu, D.-X.; Xu, Y.-T.; Wang, C.; Zheng, K.; Zhou, D.-D.; He, C.-T.; Zhang, J.-P., A gating ultramicroporous metal-organic framework showing high adsorption selectivity, capacity and rate for xylene separation. *Science China Chemistry* **2022,** *65* (8), 1552-1558.
21. Polyukhov, D. M.; Poryvaev, A. S.; Sukhikh, A. S.; Gromilov, S. A.; Fedin, M. V., Fine-Tuning Window Apertures in ZIF-8/67 Frameworks by Metal Ions and Temperature for High-Efficiency Molecular Sieving of Xylenes. *ACS Applied Materials & Interfaces* **2021,** *13* (34), 40830-40836.
22. Bux, H.; Chmelik, C.; van Baten, J. M.; Krishna, R.; Caro, J., Novel MOF-Membrane for Molecular Sieving Predicted by IR-Diffusion Studies and Molecular Modeling. *Advanced Materials* **2010,** *22* (42), 4741-4743.
23. Polyukhov, D. M.; Poryvaev, A. S.; Gromilov, S. A.; Fedin, M. V., Precise Measurement and Controlled Tuning of Effective Window Sizes in ZIF-8 Framework for Efficient Separation of Xylenes. *Nano Letters* **2019,** *19* (9), 6506-6510.
24. Laha, S.; Haldar, R.; Dwarkanath, N.; Bonakala, S.; Sharma, A.; Hazra, A.; Balasubramanian, S.; Maji, T. K., A dynamic chemical clip in supramolecular framework for sorting alkylaromatic isomers using thermodynamic and kinetic preferences. *Angewandte Chemie* **2021,** *133* (36), 20074-20080.
25. Lannoeye, J.; Van de Voorde, B.; Bozbiyik, B.; Reinsch, H.; Denayer, J.; De Vos, D., An aliphatic copper metal-organic framework as versatile shape selective adsorbent in liquid phase separations. *Microporous and Mesoporous Materials* **2016,** *226*, 292-298.
26. Gee, J. A.; Zhang, K.; Bhattacharyya, S.; Bentley, J.; Rungta, M.; Abichandani, J. S.; Sholl, D. S.; Nair, S., Computational Identification and Experimental Evaluation of Metal–Organic Frameworks for Xylene Enrichment. *The Journal of Physical Chemistry C* **2016,** *120* (22), 12075-12082.
27. Vermoortele, F.; Maes, M.; Moghadam, P. Z.; Lennox, M. J.; Ragon, F.; Boulhout, M.; Biswas, S.; Laurier, K. G. M.; Beurroies, I.; Denoyel, R.; Roeffaers, M.; Stock, N.; Düren, T.; Serre, C.; De Vos, D. E., p-Xylene-Selective Metal–Organic Frameworks: A Case of Topology-Directed Selectivity. *Journal of the American Chemical Society* **2011,** *133* (46), 18526-18529.
28. Alaerts, L.; Kirschhock, C. E. A.; Maes, M.; van der Veen, M. A.; Finsy, V.; Depla, A.; Martens, J. A.; Baron, G. V.; Jacobs, P. A.; Denayer, J. F. M.; De Vos, D. E., Selective Adsorption and Separation of Xylene Isomers and Ethylbenzene with the Microporous Vanadium(IV) Terephthalate MIL-47. *Angewandte Chemie International Edition* **2007,** *46* (23), 4293-4297.
29. Yang, X.; Zhou, H.-L.; He, C.-T.; Mo, Z.-W.; Ye, J.-W.; Chen, X.-M.; Zhang, J.-P., Flexibility of Metal-Organic Framework Tunable by Crystal Size at the Micrometer to Submillimeter Scale for Efficient Xylene Isomer Separation. *Research* **2019,** *2019*.

30. Lin, Y.; Zhang, J.; Pandey, H.; Dong, X.; Gong, Q.; Wang, H.; Yu, L.; Zhou, K.; Yu, W.; Huang, X.; Thonhauser, T.; Han, Y.; Li, J., Efficient separation of xylene isomers by using a robust calcium-based metal–organic framework through a synergetic thermodynamically and kinetically controlled mechanism. *Journal of Materials Chemistry A* **2021,** *9* (46), 26202-26207.
31. Rasouli, M.; Yaghobi, N.; Gilani, S. Z. M.; Atashi, H.; Rasouli, M., Influence of monovalent alkaline metal cations on binder-free nano-zeolite X in para-xylene separation. *Chinese Journal of Chemical Engineering* **2015,** *23* (1), 64-70.
32. Rasouli, M.; Yaghobi, N.; Allahgholipour, F.; Atashi, H., Para-xylene adsorption separation process using nano-zeolite Ba-X. *Chemical Engineering Research and Design* **2014,** *92* (6), 1192-1199.
33. Hurst, J. E.; Cheng, L. S.; Broach, R. W., Para-xylene-separation with aluminosilicate X-type zeolite compositions with low LTA-type zeolite. Google Patents: 2013.
34. Silva, M. S.; Moreira, M. A.; Ferreira, A. F.; Santos, J. C.; Silva, V. M.; Sá Gomes, P.; Minceva, M.; Mota, J. P.; Rodrigues, A. E., Adsorbent evaluation based on experimental breakthrough curves: separation of p-xylene from C8 isomers. *Chemical engineering & technology* **2012,** *35* (10), 1777-1785.
35. Rasouli, M.; Yaghobi, N.; Chitsazan, S.; Sayyar, M. H., Influence of monovalent cations ion-exchange on zeolite ZSM-5 in separation of para-xylene from xylene mixture. *Microporous and mesoporous materials* **2012,** *150*, 47-54.
